# Global transmission of extended-spectrum cephalosporin resistance in *Escherichia coli* driven by epidemic plasmids

**DOI:** 10.1016/j.ebiom.2024.105097

**Published:** 2024-04-11

**Authors:** Roxana Zamudio, Patrick Boerlin, Michael R. Mulvey, Marisa Haenni, Racha Beyrouthy, Jean-Yves Madec, Stefan Schwarz, Ashley Cormier, Gabhan Chalmers, Richard Bonnet, George G. Zhanel, Heike Kaspar, Alison E. Mather

**Affiliations:** a Quadram Institute Bioscience, Norwich Research Park, Norwich NR4 7UQ, United Kingdom; b Department of Pathobiology, University of Guelph, Guelph N1G 2W1, Canada; c National Microbiology Laboratory, Public Health Agency of Canada, Winnipeg, Manitoba R3E 3R2, Canada; d Unité Antibiorésistance et Virulence Bactériennes, Anses - Université de Lyon, Lyon 69007, France; e Microbes Intestin Inflammation et Susceptibilité de l'Hôte (M2ISH), Faculté de Médecine, Université Clermont Auvergne, Clermont-Ferrand 63001, France; f Centre National de Référence de la Résistance Aux Antibiotiques, Centre Hospitalier Universitaire de Clermont-Ferrand, Clermont-Ferrand 63000, France; g Institute of Microbiology and Epizootics, School of Veterinary Medicine, Freie Universität Berlin, Berlin 14163, Germany; h Veterinary Centre for Resistance Research (TZR), School of Veterinary Medicine, Freie Universität Berlin, Berlin 14163, Germany; i Department of Medical Microbiology and Infectious Diseases, Max Rady College of Medicine, Rady Faculty of Health Sciences, University of Manitoba, Winnipeg, Manitoba R3E 0J9, Canada; j Department Method Standardisation, Resistance to Antibiotics Unit Monitoring of Resistance to Antibiotics, Federal Office of Consumer Protection and Food Safety, Berlin 12277, Germany; k University of East Anglia, Norwich NR4 7TJ, United Kingdom

**Keywords:** Epidemic plasmid, *Escherichia coli*, AMR, ESBL, AmpC, Whole genome sequencing

## Abstract

**Background:**

Extended-spectrum cephalosporins (ESCs) are third and fourth generation cephalosporin antimicrobials used in humans and animals to treat infections due to multidrug-resistant (MDR) bacteria. Resistance to ESCs (ESC-R) in Enterobacterales is predominantly due to the production of extended-spectrum β-lactamases (ESBLs) and plasmid-mediated AmpC β-lactamases (AmpCs). The dynamics of ESBLs and AmpCs are changing across countries and host species, the result of global transmission of ESC-R genes. Plasmids are known to play a key role in this dissemination, but the relative importance of different types of plasmids is not fully understood.

**Methods:**

In this study, *Escherichia coli* with the major ESC-R genes *bla*_CTX-M-1_, *bla*_CTX-M-15_, *bla*_CTX-M-14_ (ESBLs) and *bla*_CMY-2_ (AmpC), were selected from diverse host species and other sources across Canada, France and Germany, collected between 2003 and 2017. To examine in detail the vehicles of transmission of the ESC-R genes, long- and short-read sequences were generated to obtain complete contiguous chromosome and plasmid sequences (n = 192 ESC-R *E. coli*). The types, gene composition and genetic relatedness of these plasmids were investigated, along with association with isolate year, source and geographical origin, and put in context with publicly available plasmid sequences.

**Findings:**

We identified five epidemic resistance plasmid subtypes with distinct genetic properties that are associated with the global dissemination of ESC-R genes across multiple *E. coli* lineages and host species. The IncI1 pST3 *bla*_CTX-M-1_ plasmid subtype was found in more diverse sources than the other main plasmid subtypes, whereas IncI1 pST12 *bla*_CMY-2_ was more frequent in Canadian and German human and chicken isolates. Clonal expansion also contributed to the dissemination of the IncI1 pST12 *bla*_CMY-2_ plasmid in ST131 and ST117 *E. coli* harbouring this plasmid. The IncI1 pST2 *bla*_CMY-2_ subtype was predominant in isolates from humans in France, while the IncF F31:A4:B1 *bla*_CTX-M-15_ and F2:A-:B- *bla*_CTX-M-14_ plasmid subtypes were frequent in human and cattle isolates across multiple countries. Beyond their epidemic nature with respect to ESC-R genes, in our collection almost all IncI1 pST3 *bla*_CTX-M-1_ and IncF F31:A4:B1 *bla*_CTX-M-15_ epidemic plasmids also carried multiple antimicrobial resistance (AMR) genes conferring resistance to other antimicrobial classes. Finally, we found genetic signatures in the regions surrounding specific ESC-R genes, identifying the predominant mechanisms of ESC-R gene movement, and using publicly available databases, we identified these epidemic plasmids from widespread bacterial species, host species, countries and continents.

**Interpretation:**

We provide evidence that epidemic resistance plasmid subtypes contribute to the global dissemination of ESC-R genes, and in addition, some of these epidemic plasmids confer resistance to multiple other antimicrobial classes. The success of these plasmids suggests that they may have a fitness advantage over other plasmid types and subtypes. Identification and understanding of the vehicles of AMR transmission are crucial to develop and target strategies and interventions to reduce the spread of AMR.

**Funding:**

This project was supported by the 10.13039/100013281Joint Programming Initiative on Antimicrobial Resistance (JPIAMR), through the 10.13039/501100000265Medical Research Council (MRC, MR/R000948/1), the 10.13039/501100000024Canadian Institutes of Health Research (CFC-150770), and the Genomics Research and Development Initiative (10.13039/501100000023Government of Canada), the 10.13039/501100002347German Federal Ministry of Education and Research (BMBF) grant no. 01KI1709, the French Agency for food environmental and occupational health & safety (Anses), and the French National Reference Center (CNR) for antimicrobial resistance. Support was also provided by the 10.13039/501100000268Biotechnology and Biological Sciences Research Council (BBSRC) through the BBSRC Institute Strategic Programme Microbes in the Food ChainBB/R012504/1 and its constituent project BBS/E/F/000PR10348 (Theme 1, Epidemiology and Evolution of Pathogens in the Food Chain).

**Research in context**

**Evidence before this study**

We searched the PubMed database up to 1st November 2022, without date or language restrictions, using the terms ((ESBL) OR (AmpC)) AND (plasmid) AND (*Escherichia coli*) AND (transmission) AND (sequencing). This research strategy identified 180 studies, of which we excluded 14 that were not open access and 11 that examined organisms other than *E. coli*, resulting in 155 open access studies. Of these 155, six were reviews on ESBL and AmpC genes; eight were related to plasmid conjugation assays or algorithms to predict AmpC genes or assays to test the stability of specific plasmids; 62 used polymerase chain reaction amplification (PCR) to detect the ESBL and/or AmpC genes and/or plasmid replicons and/or insertion sequences by detecting specific target sequences; 52 used short-read whole genome sequencing and 27 used short-read and long-read sequences to investigate ESC-R in *E. coli*. Of these latter 27 studies, the vast majority included only a small number of long-read sequenced genomes (between 1 and 45 genomes), with only one study examining a large (n = 149) number of long-read sequenced genomes, but this was limited to ESBL *E. coli* from humans in the United States. Recently, a study describing >2000 *E. coli* long-read genomes from humans in Norway was pre-printed. Overall, these previous studies identified ESC-R genes and mobile genetic elements associated with these genes in *E. coli*, but are generally limited to a specific geographical origin, host species and/or ESC-R gene(s). The few publications (n = 3) that examined the global distribution of ESC-R genes are reviews, although a recent publication demonstrated temporal changes of ESBL and AmpC genes between continents and sources, and the relative importance of plasmids in ESC-R gene transmission. Finally, a recent single review explored IncI1 plasmids in a global context but this was limited to the few genes used in the plasmid multilocus sequence typing (pMLST) scheme. Therefore, while complete plasmid sequences, facilitated by long-read sequences, are required for full resolution of the vehicles of AMR spread, to date there has not been a large-scale comprehensive study of contiguated ESC-R plasmids in a global context.

**Added value of this study**

Our approach using long-read and short-read genome sequences of ESC-R *E. coli* at large scale from multiple countries and host species revealed that epidemic plasmids are key to the global dissemination of ESC-R. Furthermore, some of these epidemic plasmids also contribute to the spread of multidrug-resistant (MDR) bacteria in co-occurrence with the ESC-R genes. Through this large comparative study across multiple countries, sources and different *E. coli* backgrounds, we show that there are differences in the dynamics of these epidemic plasmids, and that the surrounding genes are specific to certain ESC-R genes, which could potentially be targeted to remove epidemic plasmids from the *E. coli* population.

**Implications of all the available evidence**

The epidemic plasmids could be targets for surveillance studies and for developing strategies to reduce the transmission of AMR. Future studies can use a similar One Health approach with the increasingly feasible large-scale long-read sequencing to identify other epidemic resistance and MDR plasmids. Further studies focussed on these plasmids could help support the hypothesis that these plasmids carry determinants for enhanced fitness in isolates harbouring them.

## Introduction

Extended-spectrum cephalosporins (ESCs) are critically important antimicrobial agents for human and animal health to treat infections due to multidrug-resistant (MDR) bacteria.^1^ Resistance to ESCs is predominantly mediated through extended-spectrum β-lactamases (ESBLs) and plasmid-mediated AmpC β-lactamases (AmpCs).^2^ Since the first reports of ESBLs in Europe and AmpCs in North America in Enterobacterales from humans and food-producing animals, respectively,^3^^,^^4^ these ESC resistance (ESC-R) genes have disseminated worldwide.^2^ In Enterobacterales, the transmission and persistence of these ESC-R genes are mediated by a combination of clonal expansion of resistant lineages and horizontal gene transfer of plasmids carrying the ESC-R genes.^5^^,^^6^ For example, *E. coli* sequence type (ST) 131 is a human-associated pathogen carrying the ESBL gene *bla*_CTX-M-15_, and global spread of ESC-R ST131 was due to clonal expansion of the lineage harbouring a plasmid with *bla*_CTX-M-15_.^7^ In contrast, the spread of other common ESBL genes, such as *bla*_CTX-M-1_ and *bla*_CTX-M-14_, is primarily due to plasmids.^6^^,^^8^ Horizontal gene transfer via plasmids is also the main mechanism of transmission for the AmpC gene *bla*_CMY-2_.^9^ In a recent international comprehensive study, we showed changes in the dynamics of different ESC-R genes by compartments (country + source). This previous study also evaluated the relative importance of clonal expansion versus putative plasmids for the most common ESC-R genes, suggesting the main mechanisms of transmission were dominant plasmids for *bla*_CTX-M-1_ and *bla*_CTX-M-14_, diverse plasmids for *bla*_CMY-2_, clonal expansion for *bla*_CTX-M-15_, and a combination of diverse plasmids in common STs for *bla*_SHV-12_.^10^

Previous studies investigating plasmid-mediated ESC-R genes found *bla*_CMY-2_ genes mostly on IncI1 and IncK plasmids,^11^ *bla*_CTX-M-1_ on IncI1^12^ and IncN plasmids,^13^ *bla*_CTX-M-15_ on IncF plasmids^14^ and *bla*_CTX-M-14_ primarily on IncF, IncK and IncI1 plasmids.^6^^,^^15^ The initial association between plasmids and ESC-R genes was obtained using polymerase chain reaction (PCR) amplification,^13^^,^^15^^,^^16^ by recording the concurrent presence of the target AMR genes and specific plasmid replicon types within transconjugants. In addition, associations with specific insertion sequences (IS) has been also determined by PCR.^15^ However, such data are limited to specific genes determined by PCR within a plasmid. With the widespread availability of short-read whole genome sequence (WGS) data and algorithms specifically designed to assemble,^17^ reconstruct^18^ and classify^19^ plasmids, greater resolution on their composition has been possible. However, the limitation of this approach is that plasmid assemblies derived from short-reads are usually fragmented due to repetitive elements, meaning that reliable complete plasmid sequences are often not obtainable.^11^^,^^12^^,^^20^ The presence of multiple plasmids within a single bacterium or the insertion of plasmid sequences within the chromosome present similar challenges to complete plasmid assembly. The use of long-read sequencing overcomes these limitations, through the generation of fully contiguated plasmid sequences. A recent study of turkey-derived *E. coli* isolates in Canada used both short- and long-read data to obtain complete circular plasmids and demonstrated that *bla*_CTX-M-1_ was predominantly carried by IncI1 pST3 plasmids, while *bla*_CTX-M-15_ was predominantly carried by a variety of IncF plasmids.^21^ A different study of diverse animal isolates showed that the IncI1 pST3 plasmids were genetically highly similar, and were contributing to the spread of *bla*_CTX-M-1_ in Canada.^8^ IncI1 pST3 plasmid subtypes have been termed “epidemic plasmids”.^8^^,^^21^ The term “epidemic plasmid” was used previously to define highly prevalent plasmids that were detected over time in diverse countries and in bacteria of different sources (e.g., human, animals and environment), and that have the capability of acquiring AMR genes and rapid dissemination among Enterobacterales.^22^, ^23^, ^24^, ^25^

Despite the recent numbers of genomic studies reporting plasmid sequences harbouring ESC-R genes,^12^^,^^14^^,^^26^ most are based on short-read data^12^^,^^14^^,^^26^ and thus, there is often ambiguity as to the genomic location of the ESC-R genes -- plasmid or chromosome -- and complete plasmid identification. The majority of studies using long-read sequences generally focus on particular geographical regions,^21^^,^^27^, ^28^, ^29^, ^30^, ^31^ certain ESC-R genes^8^^,^^32^ and/or sources.^33^, ^34^, ^35^ A recent global food products study identified a potential epidemic plasmid, but this was limited to a small number of genomes (n = 7),^36^ and a recent review exploring IncI1 ESC-R plasmids in a global context was based on the five genes of the pMLST scheme.^37^ This limits our understanding of the global emergence and dissemination of ESC-R genes and the relative importance of the roles of plasmids and clonal expansion. To gain further insight into the mechanisms underlying the spread of ESC-R genes worldwide, we used both long-read and short-read WGS data to obtain complete or near-complete chromosome and plasmid sequences in a diverse set of 204 *E. coli* isolates carrying the main ESC-R genes (*bla*_CMY-2_, *bla*_CTX-M-1_, *bla*_CTX-M-15_ and *bla*_CTX-M-14_) from multiple countries and host species.^10^ We then put these results in context with the huge number of publicly available plasmid sequences. Here, we identified a limited number of plasmids carrying specific ESC-R genes that are epidemic across multiple ecological compartments and multiple countries.

## Methods

### Study design and bacterial isolate selection

The *E. coli* isolates in this study were resistant to ESCs and were selected from extensive collections of Enterobacterales (n = 1524) obtained mainly between 2008 and 2016 from human clinical cases, healthy and diseased animals, food (meat) and wastewater in Canada, France and Germany. This extensive collection was analysed in our previous study, in which we found 39 different ESC-R genes, with *bla*_CMY-2_, *bla*_CTX-M-1_, *bla*_CTX-M-14_ and *bla*_CTX-M-15_ as the main genes, and different relative importance of clonal expansion and plasmids as the main mechanism of dissemination of these ESC-R genes.^10^ In this study, a subset of 204 isolates was selected from the extensive collection in two stages: 1) *E. coli* isolates (n = 100) from 2003 to 2017 representing a subset of the most common STs (ST131, ST10, ST117, ST167, and ST617) carrying the main ESC-R genes (*bla*_CMY-2_, *bla*_CTX-M-1_, *bla*_CTX-M-14_ and *bla*_CTX-M-15_) and originating from diverse host species across Canada, France and Germany; 2) Based on the preliminary results from stage 1 of the most common plasmids, additional isolates (n = 104) were selected to provide additional representatives for those dominant plasmids, selected on the presence of the plasmid replicon type in the short-read data and representing rare STs carrying the main ESC-R genes. This resulted in a representative subset of isolates containing common and rare STs carrying the dominant ESC-R genes and plasmids across multiple sources and countries.

### Long-read sequencing and complete genome assembly

The Illumina short-read WGS data were available from our previous study as were 20 long-read genome sequences^10^ and the remaining long-read sequences were generated in this study. The isolates selected for this study were cultured in non-selective media and the DNA was extracted from pure culture, see more details in Supplementary appendix 1 p 3. Long-read sequencing was performed using a MinION device (Oxford Nanopore Technologies, Oxford, United Kingdom); details can be found in Supplementary appendix 1 p 3.

Base-calling of the fast5 files and demultiplexing of the Nanopore reads were performed using Guppy v4.5.4 through the Basecaller and Barcoder algorithms (Oxford Nanopore Technologies), respectively. Adapters from long reads were removed with Porechop v0.2.4.^38^ Filtlong v0.2.0 (https://github.com/rrwick/Filtlong) was used to remove reads <2 kbp in length and low quality reads (with min_length 2000 and min_mean_q 7 parameters, respectively); read files with very large size were reduced to a 500 Mbp subset. Hybrid assembly with short and long reads for each isolate was obtained with Unicycler v0.4.8^39^ using the “bold” mode option. Flye v2.7.1^40^ was applied (long-read-only assembly) for cases where fully contiguated plasmid sequences were not obtained with Unicycler. Unicycler and Flye assemblies were polished using the Illumina reads with Pilon v1.22^41^ with up to ten cycles of polishing.

Quality control measures were taken in the hybrid and long-read-only assemblies, which included: 1) assessment of large-scale misassemblies in the complete genomes using Socru v2.2.4^42^; 2) cross-checking for agreement of both ESC-R genes and STs between the assemblies from Illumina reads only and the hybrid and long-read-only assemblies; when discrepancy of the ESC-R gene or ST between the assemblies for the same isolate was identified, the isolate was removed from further analysis; 3) hybrid and long-read-only assemblies with high numbers of scaffolds (more than 50 scaffolds) and/or with very low long-read depth coverage (less than 5X) were removed.

### Identification of AMR and virulence genes, and typing of complete genomes

The identification of AMR genes, virulence genes and plasmid replicons was obtained through Abricate v1.0.1 (https://github.com/tseemann/abricate) using the Resfinder,^43^ VFDB^44^ and Plasmidfinder^45^ databases, respectively. MOB-suite v2.0^18^ was also used for plasmid replicon typing into plasmid incompatibility (Inc) groups; this tool was also used to perform relaxase typing into MOB types and therefore predict the mobility of the plasmid. Classification of plasmids as “conjugative plasmids” was based on the presence of a relaxase and mate-pair formation marker, as determined by MOB-suite. RFPlasmid^19^ was used to predict the chromosome or plasmid origin of the scaffolds. In a few cases where the scaffold (with ESC-R gene) did not carry any plasmid replicon available from the database but was predicted as plasmid, these were classified as non-typeable plasmids. The bacterial sequence type (ST) was assigned to each genome through the multilocus sequence typing (MLST) scheme available for *E. coli* in mlst v2.19.0 tool (https://github.com/tseemann/mlst). The plasmid sequence types (hereafter pSTs) were obtained using the plasmid MLST (pMLST) schemes^45^ available for the main plasmid Inc groups. Due to the multi-replicon (FII, FIA and FIB replicons) nature of the IncF plasmids, a replicon sequence typing scheme (RST) was used through the FAB formulae (FII:FIA:FIB)^46^ to identify the allele type and number for each replicon, thus a formula was assigned to each IncF plasmid. The pMLST and RST databases (version 2021-03-15) were available in the Center for Genomic Epidemiology (http://www.genomicepidemiology.org/) web tool through the pMLST-1.5 service. Hereafter, we use the term plasmid type to refer to the plasmid Inc group and the term subtype to refer to the pST or FAB plasmid-ESC-R gene combination.

### Plasmid replicon and plasmid sequence typing from short-read assembly

In our large collection of short-read data (n = 1818 *E. coli*),^10^ plasmid replicon typing was performed using the Plasmidfinder^45^ database with ARIBA v2.13.5,^47^ while pMLST or RST was identified using Blastn v2.10.1+^48^ and the database pmlst_db (https://bitbucket.org/genomicepidemiology/pmlst_db/src/master/).

### Relatedness and phylogeny of contiguous plasmid sequences

The relatedness of all plasmids carrying the main ESC-R genes was analysed through an alignment-free approach using Mashtree v1.2.0.^49^ Each genome was annotated with Prokka v1.13,^50^ and then Roary v3.12.0^51^ was used in three datasets: 1) the full contiguous plasmid dataset, with parameters defined as core genes in 95% of the samples with 90% identity; 2) the IncI1 plasmid dataset; and 3) the IncF plasmid dataset. The two latter datasets are subsets of the full contiguous plasmid dataset. As the plasmid sequences are more similar within than between plasmid types, more conservative parameters were used in the IncI1 and IncF plasmid datasets, defined as core genes in 95% of the samples with 95% identity. A plasmidome analysis was performed on the full contiguous plasmid dataset, where the matrix of gene presence/absence was used with the PANINI^52^ web tool to generate a gene content network. For the two predominant plasmid types, IncI1 and IncF, separate phylogenetic trees were constructed using different approaches. For the IncI1 plasmids, the resultant Roary core gene alignment was used to construct a maximum likelihood phylogenetic tree with IQ-tree v2.0.5,^53^ using the HKY and discrete GAMMA nucleotide model with 100 bootstraps.^54^ The highly diverse nature of IncF plasmids^55^ would result in a very small core gene alignment. Therefore, the IncF plasmid tree (including all IncF subtypes) was constructed using a binary matrix of gene presence/absence; the ModelFinder option in IQ-tree v2.0.5^53^ was used to determine the best substitution model for the binary data. The pairwise single nucleotide polymorphism (SNP) distances in the IncI1 core gene alignment were obtained using snp-dists v0.7.0 (https://github.com/tseemann/snp-dists). The core and accessory genes of the IncI1 and IncF plasmids were visualised through t-SNE (t-distributed stochastic neighbour embedding) algorithm^56^ with the Rtsne function (perplexity = 20) in Rtsne v0.16^57^ R^58^ package on the matrix of gene presence/absence. The plasmidome network was annotated with the metadata (source, country, and year) and then visualised. The plasmid Mashtree and phylogenetic trees were plotted along with the metadata (source, country, year, *E. coli* ST, number of AMR genes, presence/absence of AMR genes and gene configurations specific for ESC-R genes) using the ggtree v3.2.0^59^ and ggtreeExtra v1.4.0^60^ R^58^ package.

### Short-read mapping and plasmid core genome-based phylogeny

To obtain a wider context and larger sample size for the identified dominant plasmid subtypes in IncI1, short-read data from our previously analysed large collection of genomes^10^ were mapped against a reference plasmid subtype. Here, a reference plasmid subtype refers to a representative contiguated plasmid sequence from each dominant plasmid subtype—IncI1: pST3 *bla*_CTX-M-1_ (pJPI162_S12_N_3; accession number SAMEA112795418, GenBank CATKKQ010000003.1), pST12 *bla*_CMY-2_ (pJ025_N_2; accession number SAMEA112795338, GenBank CATKJD010000002.1) and pST2 *bla*_CMY-2_ (pJPI139_S64_N_3; accession number SAMEA110398700, GenBank CAXHTV010000003.1). Reads were mapped to each reference plasmid subtype with Snippy v4.3.6^61^ and a core genome obtained for those genomes containing reads which aligned with the reference plasmid subtype. Subsequently, the core genome of plasmids was used to generate a phylogenetic tree following the same parameters in IQ-tree v2.0.5^53^ as described above. Estimated pSTs for plasmids were also obtained using the short-read data to confirm plasmid sequences closely linked with the reference plasmid subtype, as they were placed in the same cluster in the phylogenetic tree. Plasmid sequences that were phylogenetically distant from the reference plasmid subtype were excluded from further analysis. Thus, a set of plasmid sequences with high genetic relatedness and belonging to the same pST as the reference plasmid subtype were recovered from the larger collection of genomes. From these related plasmids, the pairwise SNP distances in the core genome of plasmids were obtained using snp-dists v0.7.0 (https://github.com/tseemann/snp-dists). This short-read mapping approach was not utilised with IncF plasmids due to the high variability of this plasmid type. Therefore, further analysis as the one described in the following section was not performed for the epidemic IncF plasmids.

### Bacterial core gene-based phylogeny and comparison with IncI1 plasmid core genome-based phylogeny

In our previous study using short-read data,^10^ we obtained a core gene phylogenetic tree representing the population structure of *E. coli*. This phylogeny was constructed using the variable sites of a concatenated alignment of 2535 core genes present in 95% of 1818 *E. coli* genomes. By its nature, the genetic information in this core gene alignment does not include accessory genes (i.e., plasmid genes, AMR and virulence genes, and mobile genetic elements). Here, the evolutionary history of the *E. coli* chromosome and that of the IncI1 plasmids [IncI1: pST3 *bla*_CTX-M-1_ as well as pST12 and pST2 *bla*_CMY-2_] was compared by contrasting the core gene phylogeny of the chromosome with the core genome phylogeny of the plasmids with the cophylo function of the phytools v0.7.90^62^ R^58^ package; the trees with annotations were plotted with ggtree v3.2.0.^59^

### Genetic environment of the ESC-R genes in main plasmid types

The genetic environment of the ESC-R genes was analysed for the most common plasmid types identified—IncI1 and IncF—using the hybrid or long-read-only assemblies. First, it was verified that all plasmid sequences started with the replicase gene [*repA* gene (GenBank MG825376.1; AWF74794.1) for IncI1 plasmids, *repA1* (GenBank LT985271.1; SPE00793.1) and *repFIB* (GenBank FJ876827.1; ADD63678.1) for IncF plasmids]. Reposition of the replication start gene was performed in a few plasmid sequences where the replicase gene was not placed at the beginning of the sequence. Briefly, the position (coordinates) of the replication start gene was obtained with Blastn v2.10.1+,^48^ then this position was used in a Perl script (fasta_shift.pl from https://github.com/b-brankovics/fasta_tools) to determine the start position of the sequence. After confirming that all plasmid sequences began with the replication start gene, the plasmid sequences were compared and annotated using Blastn v2.10.1+^48^ and a custom database, which was built using plasmid annotations available at NCBI Nucleotide; the accession numbers of the plasmids used for this annotation can be found in Supplementary Table S1. The surrounding genes of the ESC-R genes were identified through this annotation and their synteny was visualised as linear maps of genes with genoPlotR v0.8.11^63^ R^58^ package. In addition, integrons associated with other AMR genes were identified by using IntegronFinder v2.0.1.^64^ Finally, the genoPlotR v0.8.11^63^ R^58^ package was also used to visualise the comparison of complete epidemic plasmid sequences to assess their structural variation and homologies.

### Global relatedness of epidemic plasmids

To place the epidemic plasmid subtypes identified in this study in a global context, one representative circular plasmid (pJPI229_S29_N3 [IncI1 pST2 *bla*_CMY-2_], pJ003_N_3 [IncI1 pST12 *bla*_CMY-2_], pN16-00857_N_3 [IncI1 pST3 *bla*_CTX-M-1_], pJPI110_S35_N_2 [IncF F31:A4:B1 *bla*_CTX-M-15_] and pJPI134_S59_N_3 [IncF F2:A-:B- *bla*_CTX-M-14_]) was selected for each epidemic plasmid subtype. Each of these representative circular plasmids was assessed against all plasmids in the PLSDB database v2021_06_23_v2^65^ (https://ccb-microbe.cs.uni-saarland.de/plsdb/) using the distance “Mash dist” algorithm with maximal p-value threshold set to 0.1 (default setting) and the maximal distance was set to different thresholds. The PLSDB database includes 34,513 de-duplicated complete plasmid sequences, with the most represented species being *E. coli*. To identify similar plasmids to our identified epidemic plasmids IncI1 pST2 *bla*_CMY-2_, IncI1 pST12 *bla*_CMY-2_ and IncI1 pST3 *bla*_CTX-M-1_, a maximal distance of ≤0.01 was used. This value was set as in the Mashtree analysis of this study, the distance between IncI1 epidemic plasmids was ≤0.01. For IncF epidemic plasmids, due to their variability, the threshold of the maximal distance was set to ≤0.02 for IncF F2:A-:B- *bla*_CTX-M-14_ and ≤0.03 for IncF F31:A4:B1 *bla*_CTX-M-15_ plasmids. Briefly, the Mash distance approach compares the sketch of the query plasmid to the sketches of plasmids stored in the PLSDB database to calculate their similarity. The PLSDB database includes an extensive set of complete bacterial plasmids from NCBI from RefSeq, EMBL-EBI and GenBank,^65^ but to note, identical plasmids are removed in the filtering process to construct the database so the frequency of plasmids in the PLSDB database does not reflect the true abundance in real-world settings. Through this analysis, the metadata (e.g., country, source, plasmid length) and taxonomy of the plasmid records were obtained for the hits from the query epidemic plasmid. Furthermore, the five representative epidemic plasmids mentioned above were also used as query sequences in the blastn NCBI webtool (https://blast.ncbi.nlm.nih.gov/Blast.cgi) using the nucleotide database (“nt”), which includes GenBank + EMBL + DDBJ + PDB + RefSeq sequences. A blastn threshold of ≥97% identity was used with ≥90% coverage for the IncI1 plasmid subtypes, ≥80% coverage for IncF F2:A-:B- *bla*_CTX-M-14_ and ≥70% coverage for IncF F31:A4:B1 *bla*_CTX-M-15_ plasmids.

### Statistical analysis

The proportion of pST12 and pST2 *bla*_CMY-2_ between countries and compartments (country + source) was compared through Fisher’s Exact test from the rstatix’s v0.7.0^66^ R^58^ package. The median and interquartile range (IQR) of the plasmid length were obtained for the plasmid type and subtypes; for the calculation of these statistics all data points were considered, including outliers. IQR was calculated by subtracting the 1st quartile (or 25th percentile) value from the 3rd quartile (or 75th percentile) value. The distribution of the plasmid length and number of AMR genes between the main plasmid types and subtypes were compared through Mann–Whitney U tests also available in rstatix’s v0.5.0.^66^ A Bonferroni correction was applied to account for multiple hypothesis testing. To compare the diversity of sources and STs among the main plasmid subtypes, the asymptotic Simpson’s diversity (SD) index was calculated, which was estimated along with the 95% confidence intervals through 200 bootstrap replicates, as described in Chao et al. 2014^67^ and 2016^68^ and implemented in the iNEXT v2.0.20^69^ R^58^ package. The SD was investigated in two datasets: 1) in the contiguous plasmid dataset (n = 121) obtained through combination of long and short-read data, and 2) in the dataset of plasmids recovered from the short-read mapping approach (n = 298).

### Role of funders

Funders did not have any role in study design, data collection, data analysis, interpretation, or writing of this study.

## Results

### Complete genomes for ESBL and AmpC-producing *E. coli*

After quality control, 192 *E. coli* genomes were retained from the 204 isolates selected as per study design. The set of 192 *E. coli* isolates harboured the main ESC-R genes (AmpC: *bla*_CMY-2_; ESBL: *bla*_CTX-M-1_, *bla*_CTX-M-14_ and *bla*_CTX-M-15_) and were collected between 2003 and 2017. Included isolates were from humans (n = 68), cattle (n = 55), chickens (n = 21), pigs (n = 14), dogs (n = 10), wastewater (n = 7), food (n = 6), horses (n = 4), cats (n = 4) and turkeys (n = 3). The geographical origin of the isolates included Canada (n = 79), France (n = 82) and Germany (n = 31) (Supplementary Fig. S1). Hybrid genome assemblies were taken forward for further analysis of 176 isolates while long-read-only assemblies were taken forward for four isolates (details of the genomes and the metadata can be found in Supplementary appendix 2). In total, 84.4% (162/192) of the isolates carried the ESC-R genes on plasmids, 10.9% (21/192) in the chromosome and 4.7% (9/192) in both chromosomal and plasmid sequences. More than one copy of the same ESC-R gene was observed in 19 isolates, while three isolates carried two different ESC-R genes (Supplementary Table S2). Most of the chromosomally encoded genes were *bla*_CTX-M-15_ (12/45, 26.7%) and *bla*_CTX-M-14_ (10/47, 21.3%). The chromosomally encoded ESC-R genes were found in isolates belonging to the dominant ST131 lineage, the common ST117 and ST10 lineages and also observed in rare STs (Supplementary Table S3).

### Plasmid-associated ESC-R genes and plasmidome analysis

**Fig. 1:**
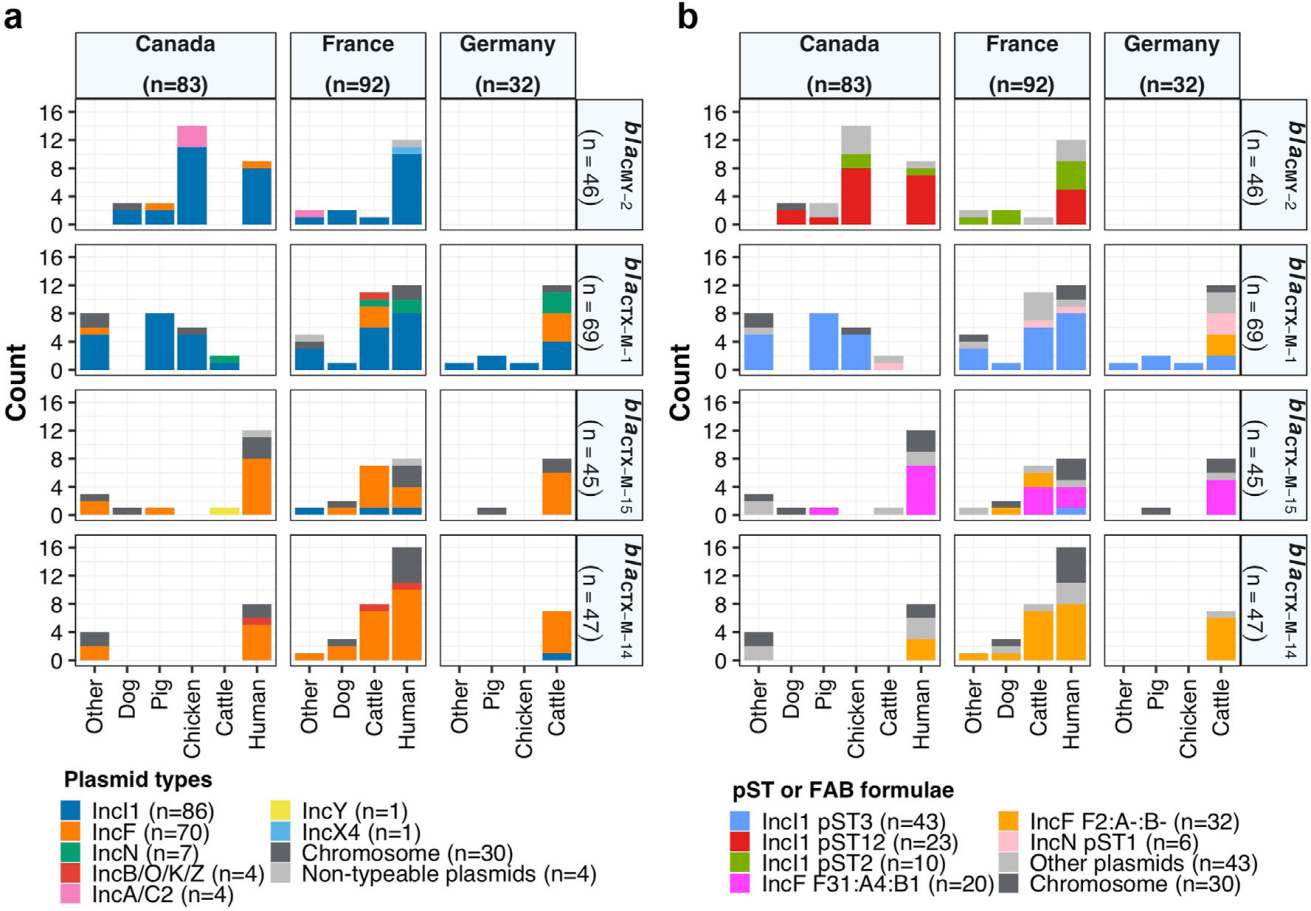
Distribution of plasmid types, pSTs and FAB formula by compartment (country + source) harbouring the main ESC-R genes. **a)** Number of each plasmid type (coloured as per inset legend). **b)** Number of each pST for IncI1 and IncN, and FAB formula for IncF plasmids (coloured as per inset legend). In source, “Other” includes the less frequent sources such as wastewater, food, horse and turkey. In the plasmid types, the “Non-typeable plasmids” category refers to those plasmid sequences that do not carry any replicon sequence included in Plasmidfinder and MOB-suite. In pST or FAB formula, the “Other plasmids” category refers to the less frequent plasmid types, subtypes and non-typeable plasmids. pST: plasmid sequence type, ESC-R: extended-spectrum cephalosporin resistance.

The results from the first stage of isolate selection identified that the most frequent plasmid types associated with ESC-R genes were IncI1 and IncF in the most common STs (ST131 [IncI1 56% and IncF 27%], ST10 [IncI1 24% and IncF 44%], ST117 [IncI1 71% and IncF 29%], ST167 [IncI1 11% and IncF 67%], and ST617 [IncF 100%]). The second stage of the sample selection revealed that other STs also harboured the main plasmid types and subtypes. Altogether, 177 plasmids carrying ESC-R genes were obtained, with plasmid types IncI1 (n = 86) and IncF (n = 70) being the most frequent, followed by IncN (n = 7), IncB/O/K/Z (n = 4), IncA/C2 (n = 4), IncY (n = 1) and IncX4 (n = 1) (Fig. 1a; Supplementary Table S4). Four plasmids were non-typeable through Plasmidfinder and MOB-suite. Most of the plasmid assemblies (165/177) were complete circular plasmids, with only a few (12/177) not circularisable. Regardless of the country and host species origin, *bla*_CMY-2_ and *bla*_CTX-M-1_ were found mostly in IncI1 plasmids, and *bla*_CTX-M-14_ and *bla*_CTX-M-15_ mainly in IncF plasmids (Fig. 1a; Supplementary Fig. S1 and Table S4). When examining pSTs, the most frequent were pST3 (43/86, 50%), pST12 (23/86, 27%) and pST2 (10/86, 12%) for IncI1, and the main FAB formulae were F31:A4:B1 (20/70, 29%) and F2:A-:B- (32/70, 46%) for IncF plasmids (Fig. 1b; Supplementary Table S4).

Variations of plasmid length were observed between plasmid types (Supplementary Fig. S2), and within IncI1 the plasmid length was more homogeneous (median: 105,326 bp; IQR: 10,424 bp) compared to IncF plasmids where length was more heterogeneous (median: 83,854 bp; IQR: 73,986 bp). The IQR for IncF plasmids was 7× that of IncI1 plasmids, with the plasmid length distributions significantly different between IncI1 and IncF plasmids (Mann–Whitney U-test, p-value = 0.003). When comparing between IncI1 subtypes, the plasmid length of IncI1 pST3 (median: 108,661 bp; IQR: 3,768 bp) was larger compared to pST12 (median: 100,323 bp; IQR: 2,662 bp) (Mann–Whitney U-test, adjusted p-value < 0.0001, median difference between the two plasmid types: 8,509 bp (95%CI: 7,160–10,370 bp); Supplementary Fig. S2) and pST2 (median: 93,628 bp; IQR: 9,820 bp) (Mann–Whitney U-test, adjusted p-value = 0.008, median difference between the two plasmid types: 13,962 bp (95%CI: 8,102–16,764 bp); Supplementary Fig. S2). Within IncF, the F31:A4:B1 plasmid length (median: 156,591 bp; IQR: 33,177 bp) was approximately twice as large as that of F2:A-:B- (median: 73,813 bp; IQR: 7,892 bp) (Mann–Whitney U-test, adjusted p-value < 0.0001, median difference between the two plasmid types: 79,567 bp (95%CI: 65,742–93,150 bp); Supplementary Fig. S2). The high resolution ESC-R plasmidome analysis revealed greater differences in the gene content between IncI1 and IncF plasmids than between their plasmid subtypes (Supplementary Fig. S3).

### Genomic relatedness of plasmids harbouring the main ESC-R genes

**Fig. 2:**
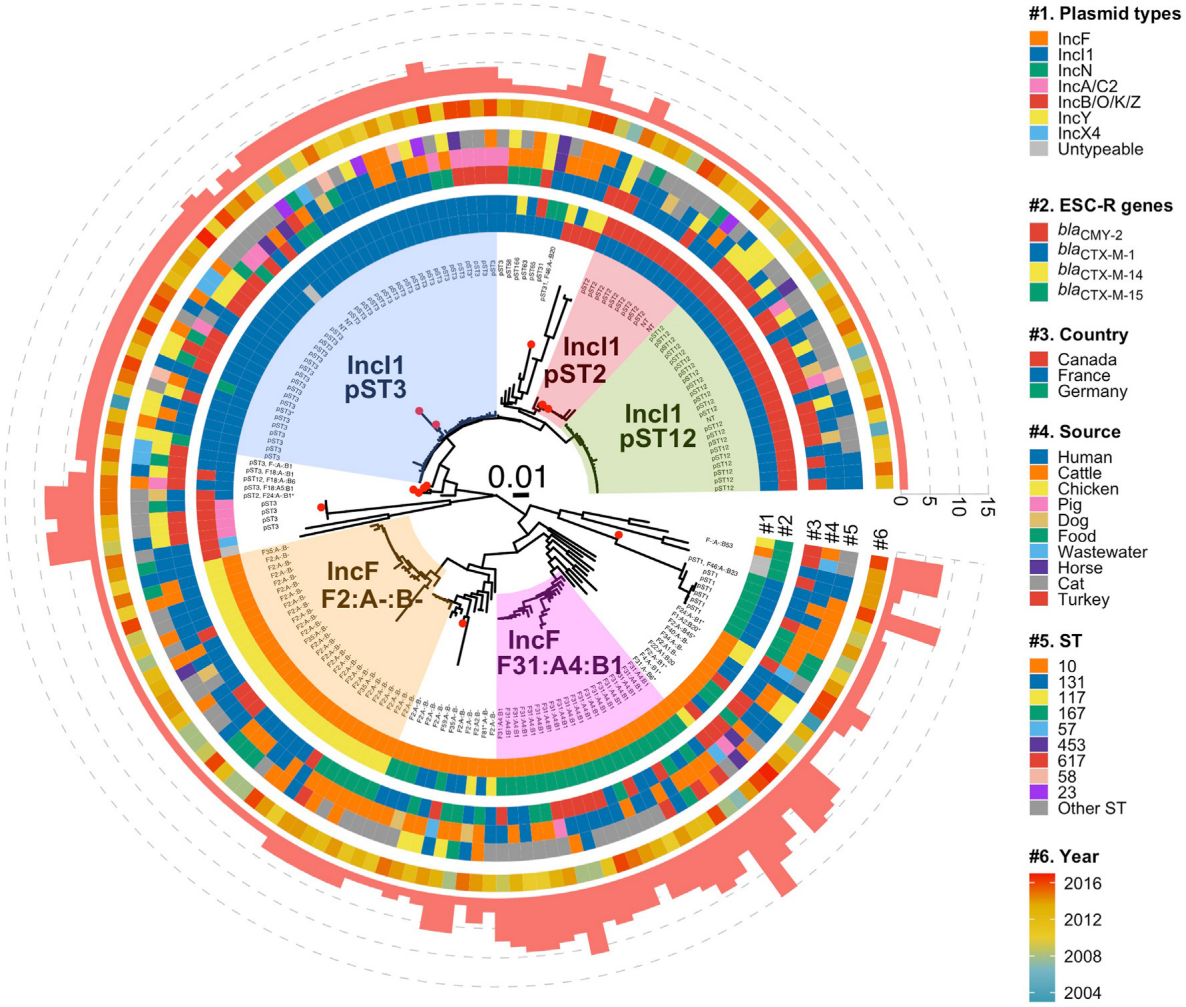
Mashtree for 177 plasmids associated with the major ESC-R genes. A red dot in the external node of the Mashtree denotes no circularisable plasmid assemblies. IncI1 pST and FAB formula for IncF plasmids are labelled next to the tips, where epidemic plasmid subtypes are highlighted. Plasmid types, ESC-R genes, country, source, *E. coli* ST and year are plotted against tips, inside to outside (coloured as per inset legend). The outer red barplot is the number of AMR genes (ESC-R genes + other AMR genes) located on each plasmid. For the plasmid types, the “Non-typeable” category represents plasmids non-typeable by Plasmidfinder and MOB-suite; in *E. coli* ST, the category “Other ST” represents STs with n < 4 genomes. pST: plasmid sequence type; ESC-R: extended-spectrum cephalosporin resistance.

Investigation of the genomic relatedness of all plasmid sequences (n = 177) showed clustering within plasmid types (Fig. 2). A total of eight pSTs were identified within 86 IncI1 plasmids (from 86 *E. coli* isolates), the most common of which were pST3 (n = 42) which was associated with *bla*_CTX-M-1_, and pST12 (n = 23) and pST2 (n = 10) associated with *bla*_CMY-2_ (Fig. 2, Supplementary Table S4). All these three main IncI1 subtypes carried a gene for a relaxase that belongs to the MOB_P_ family and were predicted as conjugative plasmids. In 70 IncF plasmids (from 70 *E. coli* isolates), multiple replicons were observed and by using the RST, 16 FAB formulae were identified. The two main FAB formulae were F31:A4:B1 (n = 20) associated with *bla*_CTX-M-15_ and F2:A-:B- (n = 26) associated with *bla*_CTX-M-14_ (Fig. 2, Supplementary Table S4); almost all (89%) of these main IncF subtypes were predicted as conjugative plasmids with a gene for relaxase belonging to the MOB_F_ family. A single pST each was observed in IncN (pST1 *bla*_CTX-M-1_) and IncA/C2 (pST3 *bla*_CMY-2_) plasmids. The other less frequent plasmid types, such as IncB/O/K/Z, were associated with the mobilisation of *bla*_CTX-M-1_ and *bla*_CTX-M-14_, IncY with that of *bla*_CTX-M-15_, and IncX4 with that of *bla*_CTX-M-1_ (Supplementary Table S4).

**Fig. 3:**
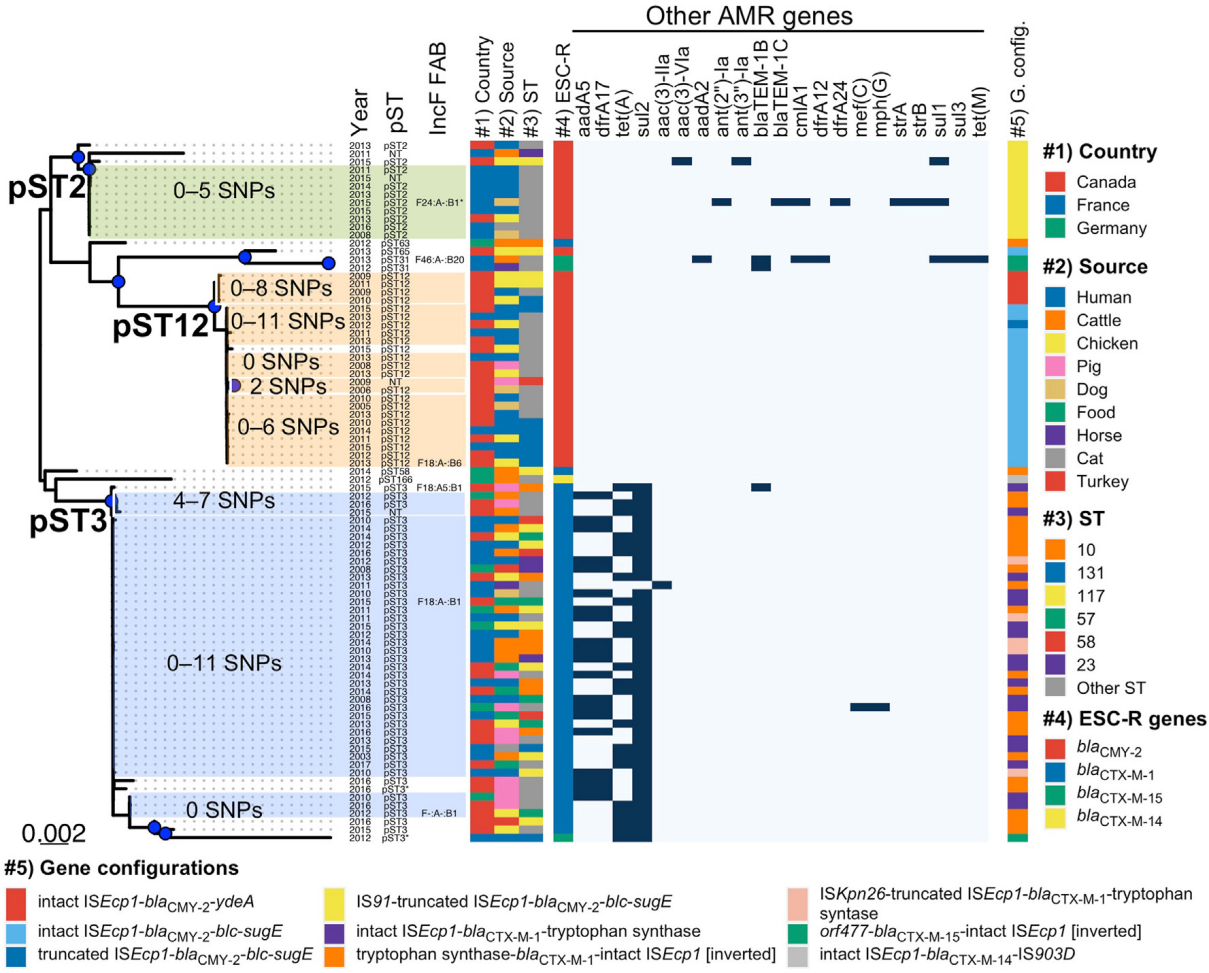
Maximum likelihood core gene tree for 86 IncI1 plasmids. Clusters of plasmids with less than 12 pairwise SNP differences are highlighted. Year, pST, IncF FAB formula, country, source, bacterial ST, ESC-R genes and other AMR genes (dark colour represents presence of the AMR gene), and gene configurations are plotted next to the tree (coloured as per inset legend). In bacterial ST, the category of “other ST” are STs with n < 4 genomes. Blue dots represent bootstrap support values greater than 95%. pST: plasmid sequence type; ESC-R: extended-spectrum cephalosporin resistance.

**Fig. 4:**
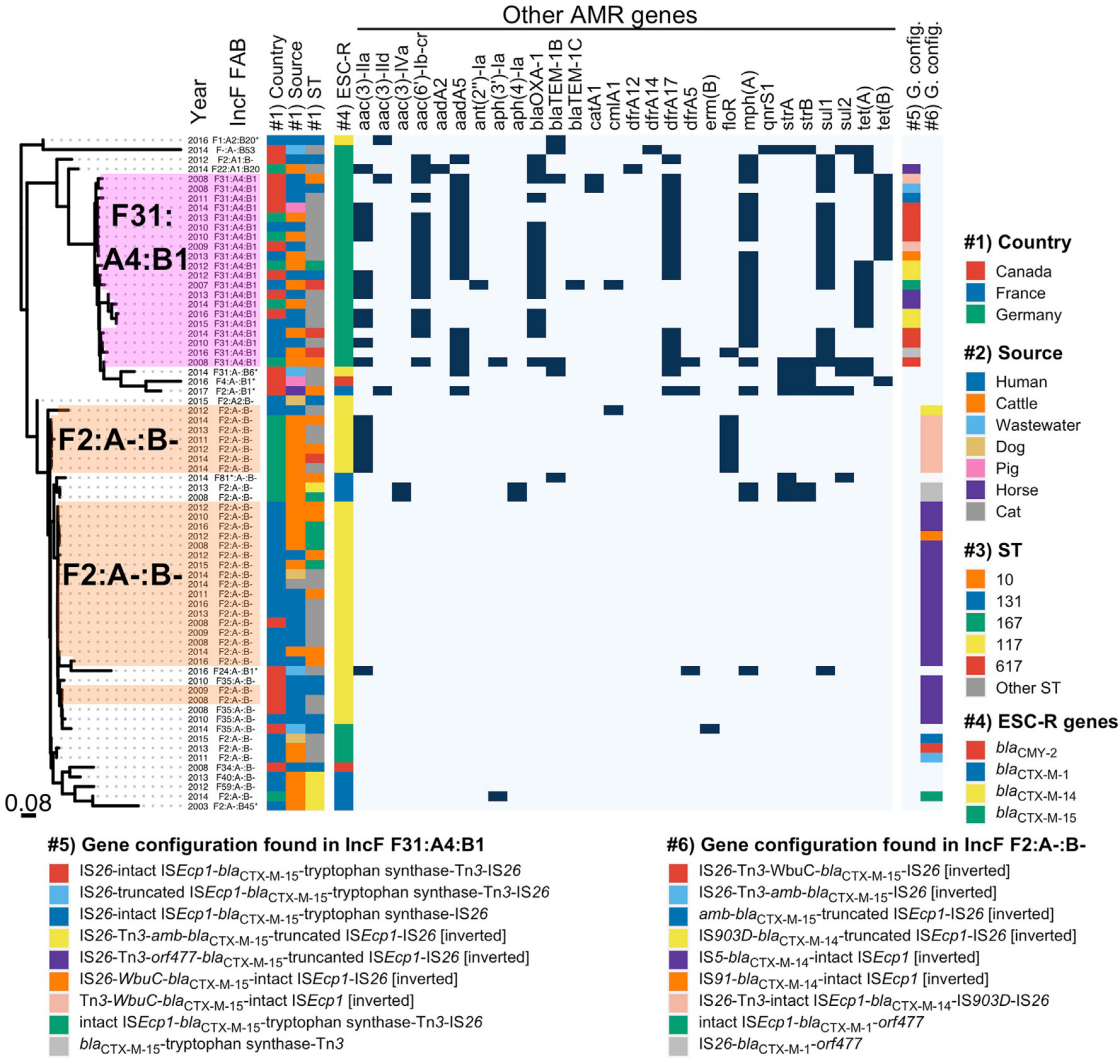
Tree based on gene presence absence matrix for 70 IncF plasmids. Clusters of similar plasmids belonging to epidemic F31:A4:B1 *bla*_CTX-M-15_ and F2:A-:B- *bla*_CTX-M-14_ subtypes are highlighted. Year, IncF FAB formula, country, source, bacterial ST, ESC-R genes, other AMR genes (dark colour represents presence of the AMR gene), and gene configurations of the ESC-R genes in IncF F31:A4:B1 and F2:A-:B- are plotted against the tree (coloured as per inset legend). STs with <4 genomes were categorised as “Other ST”. ESC: extended-spectrum cephalosporin resistance.

The gene content (plasmidome) and plasmid length were highly similar among plasmids within each of the five predominant plasmid subtypes in IncI1 [pST3 *bla*_CTX-M-1_, pST2 *bla*_CMY-2_ and pST12 *bla*_CMY-2_] and IncF [F31:A4:B1 *bla*_CTX-M-15_ and F2:A-:B- *bla*_CTX-M-14_] (Fig. 2; Supplementary Figs. S2 and S3). A longer plasmid length (median: 156,591 bp; IQR: 33,177 bp) was observed for the subtype IncF F31:A4:B1 *bla*_CTX-M-15_ (Supplementary Fig. S2), which also had a high co-occurrence of other multiple AMR genes (median: 6 AMR genes; range: 2–14 AMR genes) when compared with the other main plasmid subtypes (Mann–Whitney U-test, adjusted p-value < 0.0001) (Figs. 2, Fig. 3, Fig. 4). The second largest plasmid subtype length was that of IncI1 pST3 *bla*_CTX-M-1_ (median: 108,650 bp; IQR: 3,829 bp) (Supplementary Fig. S2) which also carried other AMR genes (median: 3 AMR genes; range: 1–5 AMR genes). Despite the gene content being highly similar within each plasmid subtype, in the gene presence/absence-based tree six out of 26 IncF F2:A-:B- *bla*_CTX-M-14_ plasmids were placed in a single cluster and were linked with German cattle origin from different years (2011–2014) (Fig. 4). Overall, the five predominant plasmid subtypes were common across different genetic backgrounds (different *E. coli* STs, both common and rare STs) and found in diverse origins (human, animals and environment) from both North America (Canada) and Europe (France and Germany) (Fig. 2).

### Epidemic IncI1 plasmid subtypes spreading *bla*_CMY-2_ and *bla*_CTX-M-1_

A core gene alignment approach was applied to IncI1 plasmids harbouring ESC-R genes. A total of 52 core genes was obtained, and the length of the concatenated aligned core gene alignment was 44,022 bp, representing 42.7% of the average length (103,063 bp) of IncI1 plasmids in this study. Three main clusters were observed in the core gene phylogenetic tree, representing pST12 *bla*_CMY-2_, pST2 *bla*_CMY-2_ and pST3 *bla*_CTX-M-1_ (Fig. 3). Less than 12 pairwise SNP differences were observed among plasmids within each plasmid subtype (Fig. 3; Supplementary Fig. S4). These highly similar epidemic IncI1 subtypes were observed in diverse lineages from different sources and continents. A high similarity within each plasmid subtype was also observed in the accessory genes (Supplementary Fig. S5), as well as along the plasmid sequences (Supplementary Figs. S6 and S7). However, two main differences were observed between IncI1 major plasmid subtypes: 1) pST3 *bla*_CTX-M-1_ was observed in more diverse sources than pST12 *bla*_CMY-2_ and pST2 *bla*_CMY-2_ (Supplementary Fig. S8); 2) pST3 had frequent co-occurrence of *bla*_CTX-M-1_ with other AMR genes (Supplementary Table S5), with two AMR gene profiles being common—*bla*_CTX-M-1_-*aadA5*-*dfrA17*-*sul2* and *bla*_CTX-M-1_-*sul2*-*tet*(A) (Supplementary Fig. S9 and Table S5)—which were not observed in the other plasmid subtypes. Regardless of the country of origin, these two AMR gene profiles were observed in diverse source origins (Fig. 3). With respect to virulence genes, very few of the major IncI1 plasmid subtypes carried *iroBCDEN* (salmochelin operon) and/or *iucBCD* (aerobactin operon) [IncI1 pST3 *bla*_CTX-M-1_: *iroBCDEN* and/or *iucBCD* (n = 3); IncI1 pST12 *bla*_CMY-2_: *iroBCDEN* and *iucBCD* (n = 1); IncI1 pST2 *bla*_CMY-2_: *iroBCDEN* (n = 1)].

To get a wider context of the global distribution of these epidemic IncI1 plasmid subtypes, core genomes of similar plasmids were recovered by mapping short-read data of other genomes from a previously published collection of 1,818 genomes^10^ to various reference plasmids. These reference plasmids comprised a representative plasmid sequence for each subtype: pJPI162_S12_N_3 (SAMEA112795418) for pST3 *bla*_CTX-M-1_, pJ025_N_2 (SAMEA112795338) for pST12 *bla*_CMY-2_ and pJPI139_S64_N_3 (SAMEA110398700) for pST2 *bla*_CMY-2_. After quality control, plasmid core genomes for a total of 182 pST3 *bla*_CTX-M-1_, 82 pST12 *bla*_CMY-2_ and 34 pST2 *bla*_CMY-2_ plasmids were recovered (Supplementary Figs. S10–S12). pST3 *bla*_CTX-M-1_ plasmids were found in diverse countries (Canada, France, Germany and the UK) and in more diverse (twice more) sources (humans, animals and food) than the other plasmid subtypes (Supplementary Figs. S8 and S13 and Table S6); this result is similar to the findings in the contiguated plasmid dataset described above. However, despite the widespread nature of the pST3 *bla*_CTX-M-1_ plasmid, this plasmid subtype was not found in human isolates from Canada (Supplementary Fig. S13). pST3 *bla*_CTX-M-1_ and pST2 *bla*_CMY-2_ plasmids were found in more diverse *E. coli* STs than pST12 *bla*_CMY-2_ plasmids (Supplementary Fig. S8 and Tables S6 and S7); for example, the number of STs found in pST3 *bla*_CTX-M-1_ and pST2 *bla*_CMY-2_ plasmids represented 4× as many of the number of STs found in pST12 *bla*_CMY-2_ plasmids. In the latter plasmid subtype, two STs were more common: ST131 (n = 30; 36%) and ST117 (n = 14; 17%) (Supplementary Table S7), resulting in a low ST diversity compared to the other IncI1 main subtypes (Supplementary Fig. S8). The two IncI1 epidemic plasmids (pST12 and pST2) carrying *bla*_CMY-2_ were found in different sources (Supplementary Fig. S14), but when comparing the proportion of these plasmids between countries, the pST12 *bla*_CMY-2_ plasmids were 3× more frequent in Canada (93%) and 2× more frequent in Germany (69%) compared to France (31%), where pST2 *bla*_CMY-2_ was more frequent in that latter country (69%) (Fisher’s Exact test, adjusted p-value < 0.01) (Supplementary Fig. S14), and when comparing by compartments (country + source) differences in the plasmid proportions were also observed (Supplementary Table S8). When analysing the distribution of the three IncI1 epidemic plasmids by main compartments (country + source) over time, the emergence of pST3 *bla*_CTX-M-1_ in chickens from Canada is evident, while both pST12 and pST2 *bla*_CMY-2_ expanded in human isolates from France (Supplementary Fig. S15). Through a comparison of plasmid core genome-based phylogeny with chromosomal core gene-based phylogeny (Supplementary Figs. S16–S18), the two ways of ESC-R dissemination are clear. First, the occurrence of plasmid subtypes in diverse genetic backgrounds demonstrates the expansion of the epidemic plasmids mediated by horizontal gene transfer. Second, dominant ST131 and common ST117 carrying one of the epidemic plasmid variants (pST12 *bla*_CMY-2_) indicates the spread of these plasmids by the clonal expansion of these two lineages (Supplementary Figs. S16–S18).

### Epidemic IncF plasmid subtypes disseminating *bla*_CTX-M-15_ and *bla*_CTX-M-14_

No core genes were identified for the IncF plasmids (including all subtypes with different FAB formulae), thus the relatedness among these plasmids was assessed through a tree based on a gene presence/absence matrix. Sixteen IncF subtypes were identified based on the FAB formula, with F31:A4:B1 *bla*_CTX-M-15_ and F2:A-:B- *bla*_CTX-M-14_ being the most dominant ones (Fig. 4). The gene content of the pangenome was similar within each main IncF subtype, but different between the subtypes (Supplementary Figs. S19 and S20). When analysing the plasmid sequences, conserved regions were observed for the epidemic plasmid F31:A4:B1 *bla*_CTX-M-15_ (Supplementary Fig. S21) and F2:A-:B- *bla*_CTX-M-14_ (Supplementary Fig. S22). The F31:A4:B1 *bla*_CTX-M-15_ subtype was observed in Canada, France and Germany, mainly in human and cattle isolates across different *E. coli* genetic backgrounds (Fig. 4; Supplementary Fig. S13). Two different clusters were identified in the IncF2:A-:B- *bla*_CTX-M-14_ subtype; one of the clusters consisted of plasmids exclusively from German cattle isolates from different years, while the other cluster consisted of plasmids from human and cattle isolates from Canada and France. The gene content between these two clusters were different (Fig. 4; Supplementary Figs. S19 and S20); for example, in the cluster linked with the German cattle isolates, all plasmids carried two additional AMR genes (*aac(3)-IIa* [aminoglycoside resistance] and *floR* [phenicol resistance]), which were absent in the other cluster of the same plasmid subtype (Fig. 4). Co-occurrence of other multiple AMR genes was very frequent in the F31:A4:B1 *bla*_CTX-M-15_ plasmids, as they also carried *mph*(A) (macrolide resistance), *aac(6’)-Ib-cr* (aminoglycoside-fluoroquinolone resistance), *aadA5* and *aac(3)-IIa* (aminoglycoside resistance), *dfrA17* (diaminopyrimidine resistance), *sul1* (sulphonamide resistance), *bla*_OXA-1_ (β-lactam resistance), *tet*(B) and *tet*(A) (tetracycline resistance); 17 AMR gene profiles were found (Supplementary Table S9). The different AMR gene profiles were associated with IS elements, class 1 integrons, CALIN cassettes and transposons (Supplementary Fig. S23). Moreover, 90% of the F31:A4:B1 *bla*_CTX-M-15_ plasmids harboured the aerobactin operon *iucBCD*, which is involved in iron acquisition. In contrast, none of the F2:A-:B- *bla*_CTX-M-14_ subtype plasmids carried other AMR genes, with the exception of the German cattle isolates mentioned above. None of the F2:A-:B- *bla*_CTX-M-14_ harboured virulence genes.

### Global dissemination of epidemic plasmids

The epidemic plasmids identified in this study were queried against an extensive public database PLSDB^65^ (which contains 34,513 de-duplicated complete plasmid sequences) and the nucleotide database from NCBI (containing complete and draft genomes). The searches using the NCBI database showed 347 hits to IncI1 pST2 *bla*_CMY-2_, 209 for pST12 *bla*_CMY-2_, 160 to IncI1 pST3 *bla*_CTX-M-1_, 104 to IncF F31:A4:B1 *bla*_CTX-M-15_ and 435 hits to IncF F2:A-:B- *bla*_CTX-M-14_ epidemic plasmid. However, the reliability of these hits is difficult to assess as the majority of the genomes are draft genomes, for which confirmation of complete plasmid sequences is very difficult. To obtain a more accurate search for similar plasmids, we searched for plasmids similar to our identified epidemic plasmids within the PLSDB^65^ database, which contains complete, de-duplicated plasmid sequences. We found that 24 publicly available plasmids were similar to IncI1 pST2 *bla*_CMY-2_, 34 to IncI1 pST12 *bla*_CMY-2_, 87 to IncI1 pST3 *bla*_CTX-M-1_, 80 to IncF F31:A4:B1 *bla*_CTX-M-15_ and 73 to IncF F2:A-:B- *bla*_CTX-M-14_ epidemic plasmids. These publicly available plasmids were present in a range of bacterial species, predominantly in *E. coli*, and a few cases in *Salmonella enterica*, *Klebsiella pneumoniae*, *Shigella sonnei*, *Shigella flexneri* and *Citrobacter freundii* (Supplementary appendix 3). According to the metadata available, the publicly available plasmids that were similar to the identified epidemic plasmids originated from a wide range of countries (IncI1 pST2 *bla*_CMY-2_: Australia, Canada, China, Italy, Russia, UK and USA; IncI1 pST12 *bla*_CMY-2_: Brazil, Canada, China, Denmark, Japan, the Netherlands, UK, Uruguay and USA; IncI1 pST3 *bla*_CTX-M-1_: Australia, Canada, China, France, Norway, Portugal, Switzerland, Taiwan, the Netherlands, UK and USA; IncF F31:A4:B1 *bla*_CTX-M-15_: Australia, Brazil, Canada, China, Colombia, Ghana, Italy, Japan, Myanmar, Nigeria, Norway, Portugal, Sweden, Switzerland, Thailand, the Netherlands, UK and USA; IncF F2:A-:B- *bla*_CTX-M-14_: Australia, Canada, China, Denmark, Finland, Italy, Japan, Mexico, Norway, Pakistan, South Korea, Sweden, Switzerland, United Arab Emirates, UK, USA, and Vietnam). Similarly, there was a wide range of sources for these public plasmids including animal origin, meat, environment and human-related samples (Supplementary appendix 3).

### Genetic environment of the plasmid-associated ESC-R genes

In this study, the genetic context of ESC-R genes in the epidemic plasmids was examined by investigating five genes upstream and downstream of the ESC-R genes. This was used to construct a unique gene profile, represented by the identity and order of five genes upstream, the ESC-R gene and the identity and order of the five genes downstream, through which unique gene configurations were identified. The gene configurations represent the closest genes surrounding the ESC-R gene. Through this approach, 27 profiles were identified for the *bla*_CTX-M-1_ and *bla*_CMY-2_ genes in IncI1 plasmids (Supplementary Table S10). Despite the gene variability observed in the profiles, only three gene configurations were observed in the pST12 *bla*_CMY-2_ plasmid subtype (Supplementary Fig. S24), of which the most common was: i) intact IS*Ecp1*-*bla*_CMY-2_-*blc* (gene for an outer membrane lipoprotein)-*sugE* (gene for resistance to quaternary ammonium compounds) (n = 18/23), found in diverse sources across Canada and France (Fig. 3; Supplementary Fig. S28), followed by ii) an intact IS*Ecp1*-*bla*_CMY-2_-*ydeA* (n = 4/23), observed only in Canada among human and animal isolates, and a single case of iii) truncated IS*Ecp1*-*bla*_CMY-2_-*blc*-*sugE* (n = 1/23). pST2 *bla*_CMY-2_ consisted mostly of a single gene configuration (Supplementary Fig. S24), an IS*91*-truncated IS*Ecp1*-*blc-sugE* (n = 10/10), identified in human and animal isolates from Canada and France (Fig. 3; Supplementary Fig. S28). The genetic context for pST3 *bla*_CTX-M-1_ predominantly consisted of three gene configurations (Supplementary Fig. S25): i): intact IS*Ecp1*-*bla*_CTX-M-1_-tryptophan synthase gene (n = 16/42); ii) an inversion of the latter gene configuration (with the gene orientation of each gene having changed) (n = 21/42), and iii) IS*Kpn26*-truncated IS*Ecp1*-*bla*_CTX-M-1_-tryptophan synthase gene (n = 5/42), which was exclusive to human and animal isolates from France (Supplementary Fig. S28). Overall, the gene configurations were specific to each IncI1 ESC-R epidemic plasmid and were found in variable countries (with the exceptions described above) and host species. The genetic context of the *bla*_CTX-M-1_ in IncI1 pST3 plasmids showed its insertion between the *rci* (shufflon-specific DNA recombinase) and *pilVA* (pilus adhesin type IV) genes, while for pST12 *bla*_CMY-2_, it was integrated upstream of the *yafB-yagA* genes and for pST2 *bla*_CMY-2_, it was integrated downstream of the *yafB-yagA* genes (Supplementary Figs. S24 and S25).

The genetic context of ESC-R genes was more variable in IncF plasmids compared to IncI1 plasmids, with 44 profiles identified (Supplementary Table S11). Nine gene configurations were observed in F31:A4:B1 *bla*_CTX-M-15_ (Fig. 4; Supplementary Fig. S26 and Table S11), and these configurations were widely distributed across multiple countries in human and cattle isolates (Supplementary Fig. S28). Two predominant gene configurations were found in F2:A-B- *bla*_CTX-M-14_ plasmids (Fig. 4; Supplementary Fig. S27 and Table S11); one was found in multiple countries, while the other was exclusive to the cattle isolates from Germany (Supplementary Fig. S28). Overall, the analyses showed that there is a specific gene configuration for IncF F2:A-:B- *bla*_CTX-M-14_, but variable gene configurations for IncF F31:A4:B1 *bla*_CTX-M-15_ which match with the variable co-occurrence of multiple other AMR genes.

## Discussion

In this study a combination of short- and long-read genome sequence data were used to obtain complete bacterial chromosome and plasmid sequences for ESBL- and AmpC-producing *E. coli* from diverse host species across North America and Europe. This approach allowed definitive determination of the genomic location of ESC-R genes, and a high-resolution investigation of the vehicles of transmission for resistance to these critically important antimicrobials. Our findings are important as we demonstrate that epidemic resistance plasmids are key in the global dissemination of AMR in *E. coli*, often conferring an MDR phenotype and spreading across genetic backgrounds, geographical barriers and across the One Health spectrum.

In addition to our main findings, we observed that *bla*_CTX-M-15_ and *bla*_CTX-M-14_ were frequently found in IncF plasmid types, with *bla*_CTX-M-1_ and *bla*_CMY-2_ mostly mobilised by IncI1 plasmids, findings which have been previously reported.^6^^,^^11^^,^^70^ Plasmid type identification is usually limited to defined replicon sequences,^16^ and to further differentiate plasmids within each plasmid type, pMLST and IncF RST schemes were developed.^45^^,^^46^ Previous studies using only short-read data reported ESC-R plasmid subtypes,^9^^,^^14^^,^^20^ and recent studies using long- and short-read data have investigated the degree to which these plasmid subtypes were conserved.^8^^,^^21^^,^^27^^,^^31^ However, the majority of these previous studies were limited to a single ESC-R gene or were specific to a particular country and/or source.^8^^,^^21^^,^^27^, ^28^, ^29^, ^30^, ^31^, ^32^, ^33^, ^34^, ^35^ Therefore, here we investigated the main ESC-R plasmid types and subtypes at high resolution and at a larger scale, using ESC-R complete genomes from diverse sources and multiple countries. Our approach allowed identification of five dominant and promiscuous IncI1 and IncF plasmid subtypes. Three were IncI1 subtypes: pST3 associated with the mobilisation of *bla*_CTX-M-1_, and pST12 and pST2 with *bla*_CMY-2_; and two were IncF subtypes: F31:A4:B1 associated with *bla*_CTX-M-15_ and F2:A-:B- with *bla*_CTX-M-14_. These five plasmid subtypes can be considered epidemic as they are major contributors to the global dissemination of these ESC-R genes, identified in isolates from humans, animals and food (meat) in different *E. coli* genetic backgrounds. These features match with the definition of epidemic plasmids that was previously proposed.^22^, ^23^, ^24^ This is further supported by the identification of similar plasmids to these epidemic plasmids in the PLSDB database, showing their presence in multiple bacterial species, host species, countries and continents.

Previous studies have implicated highly similar IncI1 pST3 plasmids as the main mechanism of spread for *bla*_CTX-M-1_ in isolates from turkeys^21^ and major production animals from Canada,^8^ veal calves and humans from France,^26^^,^^71^ food isolates from Germany,^12^ wild animals from Portugal,^71^ and amongst human, animal and food products in Denmark,^29^ Reunion Island^30^ and Japan.^28^ Similarly, pST12 and pST2 *bla*_CMY-2_ subtypes were previously reported in isolates from humans, dogs and poultry in Denmark,^11^^,^^32^ humans and chickens in the Netherlands,^27^ humans, poultry and food isolates from Germany,^9^ and in dogs in France.^72^^,^^73^ Here we also found these two *bla*_CMY-2_ epidemic plasmids in multiple sources and countries. Despite previous studies identifying the IncI1 plasmids as epidemic, very few explored them at the nucleotide level (SNP differences); those that did reported few SNPs in plasmids within the same country.^27^^,^^71^ Here, we demonstrated that the IncI1 epidemic plasmids have <12 SNPs in their core gene alignment, confirming their high genetic similarity in a wide variety of bacterial backgrounds, countries and host species. With respect to the IncF plasmids, F31:A4:B1 *bla*_CTX-M-15_ has been observed previously in ST131 human isolates in the USA^74^ but also in non-ST131 *E. coli* in food product isolates from Germany.^14^ We also found F31:A4:B1 *bla*_CTX-M-15_ in cattle isolates and in multiple countries. F2:A-:B was described before as a predominant IncF plasmid subtype,^22^ and was associated with variable ESC-R genes.^75^^,^^76^ In particular, F2:A-:B- *bla*_CTX-M-14_ has been detected in veal calf isolates from France,^26^ and in isolates from healthy and diseased food-producing animals from China.^15^ We also found this epidemic plasmid not only in cattle isolates, but also in isolates from humans and companion animals. A recent study on human isolates from the United States reported the high frequency of *bla*_CTX-M-15_ and *bla*_CTX-M-14_ in IncF plasmids,^34^ although they did not investigate this down to IncF subtyping.

Our study supports the previous detection of epidemic plasmid subtypes harbouring ESC-R genes,^8^^,^^20^^,^^21^ but through our large scale genome comparison, we have also found important differences between the epidemic plasmid subtypes by countries and sources. Through the short-read mapping approach, we were able to recover plasmid sequences for the IncI1 epidemic plasmids from a larger collection of >1,800 genomes.^10^ This approach was not applied to the IncF plasmids due to their high variability^33^ with multiple copies of insertion sequences.^76^ Using the plasmids recovered from the larger collection allowed us to have a wider context for the IncI1 epidemic plasmids. All three IncI1 plasmid subtypes were found in at least three countries and in at least six different sources. The result of the comparison between pST12 *bla*_CMY-2_ and pST2 *bla*_CMY-2_ suggests that there is a geographical localisation of plasmids but this may change given the promiscuous nature of the epidemic plasmids. We also noted that despite the few observations of pST12 *bla*_CMY-2_ compared to pST2 *bla*_CMY-2_ in French human isolates, both epidemic plasmids emerged and disseminated over time in this population. This is in agreement with the increasing trend over time of *bla*_CMY-2_ in human isolates from France that we previously reported.^10^ In this study, we provide evidence that the *bla*_CMY-2_ epidemic plasmids are one the main drivers for the emergence and dissemination of *bla*_CMY-2_ across Europe and North America. The IncI1 pST3 *bla*_CTX-M-1_ was found in more diverse sources than the other plasmid subtypes. It is well known that *bla*_CTX-M-1_ is the most common ESBL gene in *E. coli* from animals,^12^ but it has also been observed in those from humans.^10^^,^^28^ In our study, highly similar IncI1 pST3 *bla*_CTX-M-1_ (n = 182) plasmids were widespread and most common in animal isolates (n = 118; 64.8%) with fewer cases in human isolates (n = 25; 13.7%) (mostly from France) and food isolates (n = 39; 21.4%). None of the human Canadian isolates carried the IncI1 pST3 *bla*_CTX-M-1_ plasmid, and this is because no *bla*_CTX-M-1_ was found in human isolates from Canada in our collection. We do not know if the absence of *bla*_CTX-M-1_ in *E. coli* isolates from Canadian humans is exclusive to our collection, but another study in the United States focused on human clinical samples also did not detect this ESBL gene.^34^ The overall results are similar between our contiguous long-read plasmid dataset and the plasmids recovered from the larger collection by the short-read mapping approach, demonstrating the representativeness of our genome selection strategy and targeted long-read sequencing.^10^ Regarding the IncF plasmids, F31:A4:B1 *bla*_CTX-M-15_ and F2:A-:B- *bla*_CTX-M-14_ were mainly found in human and cattle isolates from Canada, France and Germany. The frequent occurrence of *bla*_CTX-M-15_ and *bla*_CTX-M-14_ in human and cattle isolates has been reported before,^2^ but our results reveal the epidemic nature of these two IncF plasmid subtypes, supporting a previous review study.^70^

The presence of all five epidemic plasmid subtypes in diverse *E. coli* genetic backgrounds highlights the importance of horizontal gene transfer for the transmission of ESC-R genes. However, other mechanisms of spread also facilitate the spread of ESC-R. IncI1 pST12 *bla*_CMY-2_ was also found in the dominant ST131 clade B (*fimH22*), which is considered as an international multidrug-resistant high-risk clone,^24^ and in the common ST117 *E. coli* background. These two STs represented 53.7% of the isolates with this plasmid subtype. This explains the low ST diversity observed for the pST12 compared to pST2 and pST3 subtypes. It also suggests that spread of lineages ST131 clade B and ST117 contributes to the dissemination of pST12 *bla*_CMY-2_ in chicken and human isolates across multiple countries. This result supports previous reports, where highly similar IncI1 pST12 *bla*_CMY-2_ subtypes were identified in ST131 clade B *E. coli* from a Danish patient and imported broiler meat samples,^32^ and in humans and broilers from the Netherlands.^27^

ESBL and AmpC genes are flanked by mobile genetic elements such as IS elements, transposons, or class 1 integrons, which are responsible for capture of the gene and mobilisation into plasmids. Despite recognition of their role in the dissemination of ESC-R genes,^14^^,^^76^ the variability of the surrounding genes across main plasmid subtypes is not well understood. Here, we found IS*Ecp1* upstream of almost all ESC-R genes, with specific signatures of variation associated with specific ESC-R genes and plasmid subtypes. In IncI1, regardless of the source and country, specific gene configurations were associated with IncI1 epidemic plasmids. For example, an intact IS*Ecp1*-*bla*_CMY-2_-*blc*-*sugE*, as reported previously,^11^^,^^20^ was associated with pST12 *bla*_CMY-2_, and the truncation of IS*Ecp1* by IS*91* was associated with the pST2 *bla*_CMY-2_ plasmid. These signatures were found over time across human and animal isolates in Canada and France; the association of IS*91* with pST2 *bla*_CMY-2_ has not been reported before. IncI1 pST3 plasmids were associated with an intact IS*Ecp1*-*bla*_CTX-M-1_-tryptophan synthase gene and with an inversion of this gene configuration, in humans and farm and wild animals across Canada, France and Germany. Nevertheless, we also observed a few cases where the gene configuration was specific to a particular country: pST12 intact IS*Ecp1*-*bla*_CMY-2_-*ydeA* to Canada, and pST3 IS*Kpn26-*truncated IS*Ecp1*-*bla*_CTX-M-1_-tryptophan synthase gene to France. The IS*Kpn26*-truncated IS*Ecp1* was observed previously with *bla*_CMY-2_ in IncK plasmids,^9^ but not with IncI1 *bla*_CTX-M-1_. To confirm this specific geographical localisation for these gene configurations, further investigations using more isolates with the plasmid subtype–gene configuration combination are needed. In the case of the IncI1 pST12 *bla*_CMY-2_ and pST2 *bla*_CMY-2_ plasmids, our study is consistent with a previous study,^11^ therefore, the integration region is mostly conserved in these plasmids. The gene configurations of *bla*_CTX-M-1_ showed the gene was integrated between the shufflon-specific recombinase (*rci*) and pilus adhesin (*pilV*) genes and this is concordant with previous studies in Germany,^12^ Canada,^8^^,^^21^ the Netherlands^20^ and Portugal.^71^ Therefore, we confirmed that the insertion of *bla*_CTX-M-1_ between the *rci* and *pilV* is characteristic of IncI1 pST3. Contrary to the IncI1 plasmids, variable gene configurations with diverse orientations were observed for *bla*_CTX-M-15_ in the IncF F31:A4:B1 plasmids, where the *bla*_CTX-M-15_ was associated with IS*Ecp1* (intact or truncated) and/or IS*26* and/or Tn*3* transposon components, supporting a previous observation.^33^ The diverse mobile genetic elements found in these epidemic plasmids contribute to the variability of the gene configurations and to the variable number of multiple other AMR genes, and consequently to variable plasmid length observed in this IncF plasmid subtype.

Two epidemic plasmid subtypes are MDR plasmids: 1) IncI1 pST3, with the co-occurrence of *bla*_CTX-M-1_ with other AMR genes conferring resistance to sulphonamides and tetracyclines, or aminoglycosides, diaminopyrimidines and sulphonamides, which is concordant with previous reports,^8^^,^^21^^,^^77^ and 2) IncF F31:A4:B1, with the co-occurrence of *bla*_CTX-M-15_ with multiple other AMR genes (up to 14 AMR genes) conferring resistance to different antimicrobial classes, such as aminoglycosides, β-lactams, diaminopyrimidines, fluoroquinolones, macrolides, phenicols, sulphonamides, and tetracyclines. In this study, we provided evidence that MDR epidemic plasmids frequently carry AMR genes encoding resistance to critically important antimicrobial agents. ISs (IS*26* and IS*91*) and class 1 integrons contribute to the integration of other AMR genes in the IncI1 pST3 *bla*_CTX-M-1_ and in IncF F31:A4:B1 *bla*_CTX-M-15_ plasmids. In the latter epidemic plasmid, multiple copies (median: 5 copies; range 4–10 copies) of IS*26* were found in the multiresistance regions and this was associated with the co-occurrence of multiple AMR genes and multiple AMR gene profiles, suggesting that IS*26* is involved in supporting the dissemination of multiple AMR genes. Our result support a previous observation where different AMR gene profiles were detected in the multiresistance regions and it was proposed that this was largely due to deletions, insertions and/or rearrangements mediated by IS*26*.^76^ The high number of IS*26* could also explain why IncF F31:A4:B1 *bla*_CTX-M-15_ had a variable number of AMR gene profiles compared to other epidemic plasmids. Some of the IncF F2:A-B- *bla*_CTX-M-14_ plasmids carried two other AMR genes that conferred resistance to aminoglycosides and phenicols, which were specific to German cattle isolates, and the remaining plasmids of this subtype only carried *bla*_CTX-M-14_. However, the AMR gene content in these epidemic plasmids could change over time due to dynamic selection pressures. We identified that MDR plasmids were notably more widespread and this could be due to their co-resistance to multiple antimicrobials, while most of the non-MDR plasmids (which carry only ESC-R genes) seem to have a more local geographical localisation. These resistance plasmids might have a long-term stability and persistence in bacteria even in the absence of antimicrobial selection pressure.^24^ Recently, it was demonstrated that resistance genes increase the overall community stability in the microbiome,^78^ but further studies are necessary to understand this stability at a species level.

While our study focused on the main plasmid types IncI1 and IncF and their subtypes that contribute on the global dissemination of ESC-R genes, there are other plasmid types that contribute to the spread of ESC-R, such as IncA/C2 and IncB/O/K/Z with *bla*_CMY-2_,^9^ IncN with *bla*_CTX-M-1_, and IncI1 and IncB/O/K/Z with *bla*_CTX-M-14_.^15^ Finally, while most of the ESC-R genes were found in plasmids, these genes were integrated into the chromosome of some isolates, consistent with other studies for *bla*_CTX-M-15_,^7^^,^^14^^,^^33^ and *bla*_CMY-2_.^9^ We found that *bla*_CTX-M-15_ (26.7%) and *bla*_CTX-M-14_ (21.3%) were more frequently located in the chromosome when compared to *bla*_CTX-M-1_ (10.1%) and *bla*_CMY-2_ (2.2%). A recent publication suggested that chromosomal integration of ESBL genes could help secure the stable propagation of the bacteria regardless of the environmental pressure.^79^

While the epidemic plasmids identified in this study have succeeded in the time span over which they were sampled, we do not know when and from which source these plasmids emerged, and if or how what is classified as an epidemic plasmid may change with shifts in selection pressures over time, including changes in antibiotic use. Despite these limitations, our observations are similar to a recent study,^31^ where certain plasmid types were maintained and expanded over time in diverse bacterial genetic backgrounds. In addition, the majority of publicly available data are from high income countries, as are the samples evaluated in this study, therefore, a general limitation is that we do not know what plasmids are epidemic in the majority of lower- and middle-income countries. Better surveillance across the world and in a One Health context is needed.

In conclusion, we identified five epidemic plasmid subtypes—IncI1 pST3 *bla*_CTX-M-1_, pST12 and pST2 *bla*_CMY-2_, IncF F31:A4:B1 *bla*_CTX-M-15_ and F2:A-:B- *bla*_CTX-M-14_—and demonstrated that they play an important role in the emergence, dissemination and persistence of the predominant ESC-R genes at the intercontinental scale across different *E. coli* genetic backgrounds found in humans, animals and food, suggesting that these epidemic plasmids have the ability to adapt to different hosts. The results of this study add to our understanding of the dynamics of these epidemic plasmids in three aspects: 1) IncI1 pST3 *bla*_CTX-M-1_ epidemic plasmid has a more global pattern than IncI1 pST12 *bla*_CMY-2_ and pST2 *bla*_CMY-2_, which were common to specific geographical regions. IncF F31:A4:B1 *bla*_CTX-M-15_ and F2:A-:B- *bla*_CTX-M-14_ were found in different countries but were common in human and cattle isolates. 2) Two epidemic plasmids were MDR plasmids—IncI1 pST3 *bla*_CTX-M-1_, and IncF F31:A4:B1 *bla*_CTX-M-15_—carrying multiple different AMR genes, resulting in larger plasmid sizes than the other epidemic plasmids. This pattern of gaining other AMR genes could be adopted by the other epidemic plasmids in the future, thus, warranting close surveillance. 3) Specific gene configurations were associated with four epidemic plasmids, and this genetic signature could be used as a target for CRISPR-Cas9-directed approaches for plasmid removal.^80^ This study highlights the importance of identifying epidemic plasmids in a One Health and global context that may offer targets for surveillance studies, and potential for interventions to reduce the spread of ESC-R genes.

## Contributors

RZ, PB, MRM, MH, J-YM, SS, RB, HK and AEM conceived of and designed the study. Bacterial culture, Nanopore sequencing and data verification were done by PB, AC and GC for human, animal and environmental isolates from Canada; MRM and GGZ for human, animal and food isolates from Canada; RB and RB for human isolates from France; MH and J-YM for animal and food isolates from France; and HK and SS for animal isolates from Germany. Data analysis and verification for the whole collection was performed by RZ and AEM. RZ and AEM wrote the manuscript, and all authors edited and approved the final draft of the manuscript.

## Data sharing statement

All raw Nanopore long-read fastq data generated in this study have been deposited in the European Nucleotide Archive (ENA) under BioProject PRJEB60752 for 172 genomes and PRJEB54884 for 20 genomes. Hybrid assemblies for 188 isolates and long-read-only assembly for four isolates are also available in the ENA under the same BioProjects mentioned above. Individual accession numbers are available in the Supplementary appendix 2 file, alongside with the metadata generated in this study.

Illumina short-read data were previously reported in our previous publication,^10^ and 17 Canadian genomes are under BioProject PRJEB60750.

## Declaration of interests

AEM is an inventor on International Patent Application No. PCT/GB2023/050906 filed in the name of Quadram Institute Bioscience–Determination and quantification of the microbial communities and antimicrobial resistance genes on food. All other authors declare no competing interests.
