# Supplementary material for: Global transmission of extended-spectrum cephalosporin resistance in Escherichia coli driven by epidemic plasmids
Source: eBioMedicine. 2024 Apr 11;103:105097. doi: 10.1016/j.ebiom.2024.105097 (PMC11024496; doi:10.1016/j.ebiom.2024.105097)
Supplement: Supplementary methods, Figs. S1–S28 and Tables S1–S10 [file mmc1.docx]

**Supplementary appendix 1**

**Global transmission of extended-spectrum cephalosporin resistance in *Escherichia coli* driven by epidemic plasmids**

Roxana Zamudio^1^, Patrick Boerlin^2^, Michael R. Mulvey^3^, Marisa Haenni^4^, Racha Beyrouthy ^5,6^, Jean-Yves Madec^4^, Stefan Schwarz^7,8^, Ashley Cormier^2^, Gabhan Chalmers^2^, Richard Bonnet ^5,6^, George G. Zhanel^9^, Heike Kaspar^10^, Alison E. Mather^1,11*^

^1^Quadram Institute Bioscience, Norwich Research Park, Norwich, NR4 7UQ, United Kingdom ^2^Department of Pathobiology, University of Guelph, Guelph, N1G 2W1, Canada

^3^National Microbiology Laboratory, Public Health Agency of Canada, Winnipeg, Manitoba, R3E 3R2, Canada

^4^Unité Antibiorésistance et Virulence Bactériennes, Anses - Université de Lyon, Lyon, 69007, France

^5^Microbes Intestin Inflammation et Susceptibilité de l'Hôte (M2ISH), Faculté de Médecine, Université Clermont Auvergne, Clermont-Ferrand, 63001, France

^6^Centre National de Référence de la résistance aux antibiotiques, Centre Hospitalier Universitaire de Clermont-Ferrand, Clermont-Ferrand, 63000, France

^7^Institute of Microbiology and Epizootics, School of Veterinary Medicine, Freie Universität Berlin, Berlin, 14163, Germany

^8^Veterinary Centre for Resistance Research (TZR), School of Veterinary Medicine, Freie Universität Berlin, Berlin, 14163, Germany

^9^Department of Medical Microbiology and Infectious Diseases, Max Rady College of Medicine, Rady Faculty of Health Sciences, University of Manitoba, Winnipeg, Manitoba, R3E 0J9, Canada

^10^Department Method Standardisation, Resistance to Antibiotics Unit Monitoring of Resistance to Antibiotics, Federal Office of Consumer Protection and Food Safety, Berlin, 12277, Germany

^11^University of East Anglia, Norwich, NR4 7TJ, United Kingdom

*Corresponding author: alison.mather@quadram.ac.uk

Table of Contents

[SUPPLEMENTARY METHODS 3](#_Toc158858130)

[Section 1. Bacterial DNA extraction 3](#_Toc158858131)

[Section 2. Long-read sequencing for 204 ESC-R *E. coli* 3](#_Toc158858132)

[SUPPLEMENTARY FIGURES 4](#_Toc158858133)

[Supplementary Figure S1. 4](#_Toc158858134)

[Supplementary Figure S2. 5](#_Toc158858135)

[Supplementary Figure S3. 6](#_Toc158858136)

[Supplementary Figure S4. 7](#_Toc158858137)

[Supplementary Figure S5. 8](#_Toc158858138)

[Supplementary Figure S6. 9](#_Toc158858139)

[Supplementary Figure S7. 11](#_Toc158858140)

[Supplementary Figure S8. 13](#_Toc158858141)

[Supplementary Figure S9. 14](#_Toc158858142)

[Supplementary Figure S10. 15](#_Toc158858143)

[Supplementary Figure S11. 16](#_Toc158858144)

[Supplementary Figure S12. 17](#_Toc158858145)

[Supplementary Figure S13. 18](#_Toc158858146)

[Supplementary Figure S14. 19](#_Toc158858147)

[Supplementary Figure S15. 20](#_Toc158858148)

[Supplementary Figure S16. 21](#_Toc158858149)

[Supplementary Figure S17. 22](#_Toc158858150)

[Supplementary Figure S18. 23](#_Toc158858151)

[Supplementary Figure S19. 24](#_Toc158858152)

[Supplementary Figure S20. 25](#_Toc158858153)

[Supplementary Figure S21. 26](#_Toc158858154)

[Supplementary Figure S22. 27](#_Toc158858155)

[Supplementary Figure S23. 28](#_Toc158858156)

[Supplementary Figure S24. 29](#_Toc158858157)

[Supplementary Figure S25. 30](#_Toc158858158)

[Supplementary Figure S26. 31](#_Toc158858159)

[Supplementary Figure S27. 32](#_Toc158858160)

[Supplementary Figure S28. 34](#_Toc158858161)

[SUPPLEMENTARY TABLES 35](#_Toc158858162)

[Supplementary Table S1. 35](#_Toc158858163)

[Supplementary Table S2. 36](#_Toc158858164)

[Supplementary Table S3. 37](#_Toc158858165)

[Supplementary Table S4. 38](#_Toc158858166)

[Supplementary Table S5. 39](#_Toc158858167)

[Supplementary Table S6. 40](#_Toc158858168)

[Supplementary Table S7. 41](#_Toc158858169)

[Supplementary Table S8. 42](#_Toc158858170)

[Supplementary Table S9. 43](#_Toc158858171)

[Supplementary Table S10. 44](#_Toc158858172)

[Supplementary Table S11. 46](#_Toc158858173)

[SUPPLEMENTARY REFERENCES 48](#_Toc158858174)

# **SUPPLEMENTARY METHODS**

## **Section 1. Bacterial DNA extraction**

The isolates (n=204) selected for this study were re-grown on non-selective media sheep blood agar plates (Oxoid, Wesel, Germany and bioMérieux, Marcy l’Étoile, France) or LB agar (BD, Franklin Lakes, USA) at 37°C overnight. DNA for the Canadian, German and French animal isolates were prepared using the MasterPure DNA Purification Kit (Epicentre, Madison, WI, USA) following to the manufacturer’s recommendations. For some human and animal Canadian isolates, DNA was extracted with a Qiagen DNeasy Plate kits (Qiagen, Toronto, Canada), and for the French human isolates was used a Dneasy UltraClean Microbial kit (Qiagen, Hilden, Germany).

## **Section 2. Long-read sequencing for 204 ESC-R *E. coli***

Long-read sequencing was done on an Oxford Nanopore MinION device (Oxford Nanopore Technologies, Oxford, UK) according to the manufacturer's instructions. The sequencing libraries were performed using one-dimensional libraries with the SQK-RBK004 kit for the Canadian and German samples, and loaded onto a R9.4 flow cell. The SQK-LSK109 kit was also used for French and some Canadian samples, and loaded onto either R9.4 or FLO-MIN106 and FLO-MIN111 flow cells.

# **SUPPLEMENTARY FIGURES**


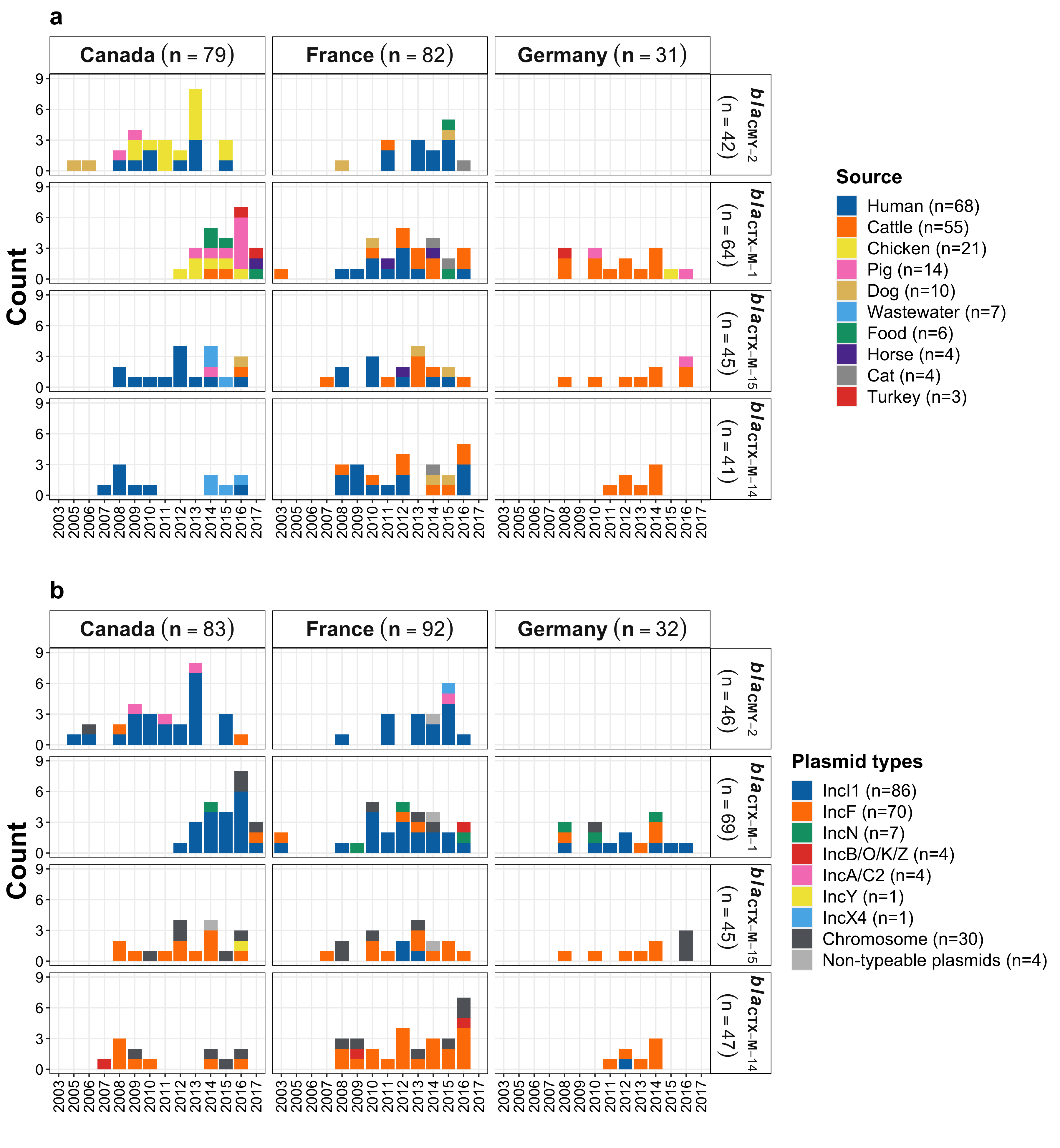


**Supplementary Figure S1. Distribution over time of *E. coli* isolates and plasmid types harbouring the main four ESC-R genes.** **a)** Number of *E. coli* isolates (n=192) over time by country and source for each main ESC-R gene. In this collection, three isolates carry two different ESC-R genes: i) *bla*_CTX-M-1_ – *bla*_CMY-2_ combination (Canada, pig, 2016); ii) *bla*_CMY-2_ – *bla*_CTX-M-14_ combination (France, human, 2013); and iii) *bla*_CMY-2_ – *bla*_CTX-M-2_ combination (France, human, 2013). Colour of the bars are associated with the source. **b)** Number of plasmid types (n=173), non-typeable plasmids (n=4) and chromosomes (n=30) carrying the main ESC-R genes over time. In this latter plot the IncHI2 *bla*_CTX-M-2_ plasmid was not included as this study is focused only on plasmids carrying the main four ESC-R genes (*bla*_CMY-2_, *bla*_CTX-M-1_, *bla*_CTX-M-15_ and *bla*_CTX-M-14_). Bars are coloured as per inset legend. ESC-R: extended-spectrum cephalosporin resistance.


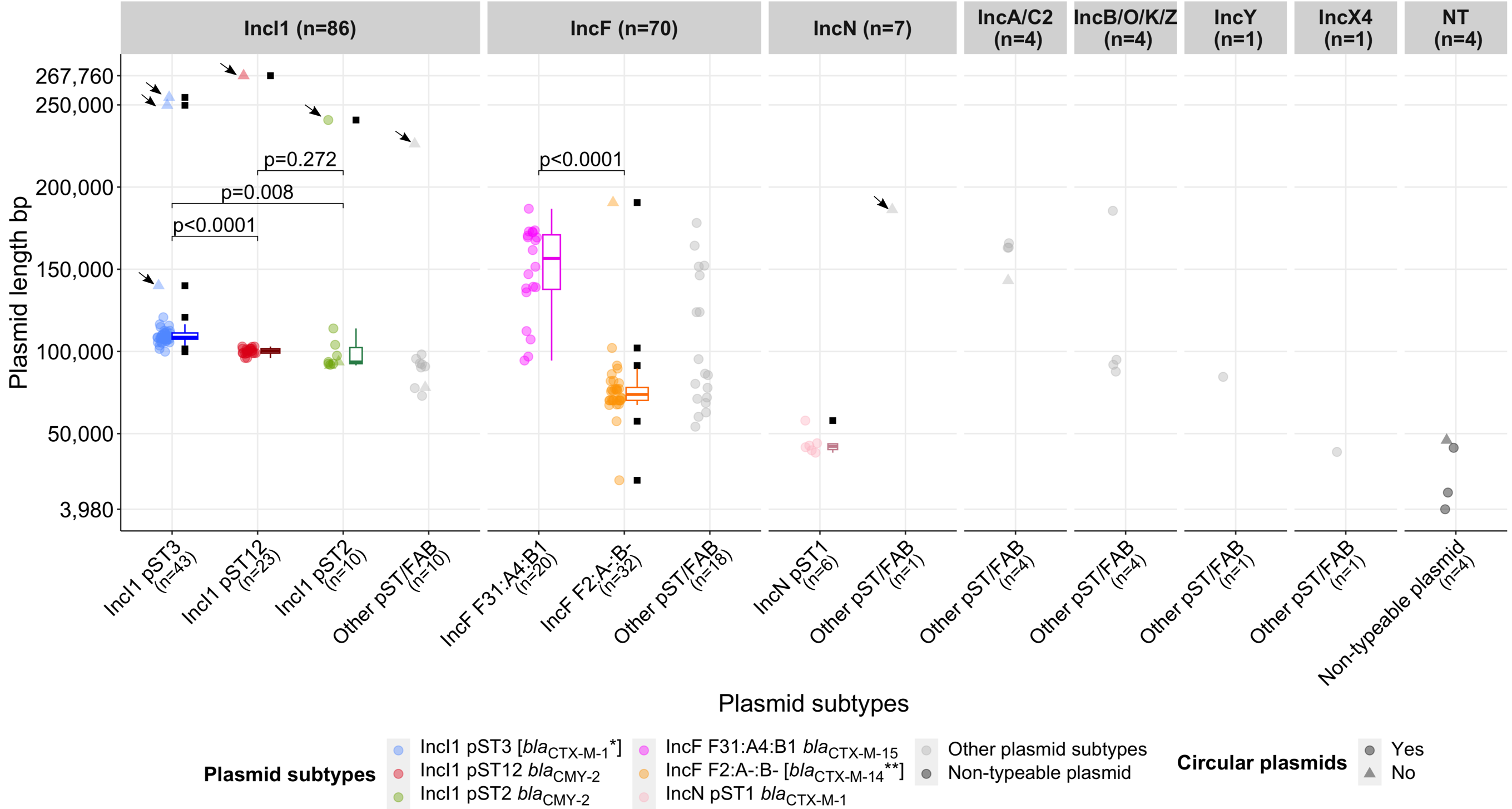


**Supplementary Figure S2. Distribution of the plasmid length amongst plasmid types and subtypes.** Circular and not circularisable plasmids are represented by the circle and triangle dot, respectively. Colour of each dot is linked with the plasmid subtype. Boxplot is displayed for only frequent plasmid subtypes, for which the plasmid length was compared through two-sided Mann-Whitney U tests. For each boxplot, the length of the box corresponds to the interquartile range with the centre line corresponding to the median (50th percentile), the boundaries of the box represent the 25th percentile (lower quartile) and 75th percentile (upper quartile). The whiskers are the two lines outside the box and their boundaries represent the minimum and maximum values; the lower whisker extends from the hinge to the smallest value at most 1.5 times the interquartile range, and the upper whisker extends from the hinge to the largest value no further than 1.5 times the interquartile range from the hinge. Values (black square) outside whiskers represents outliers. p-values (Mann-Whitney U test) are reported in the figure. The arrow next to some data points indicate a plasmid fusion case; six IncI1 and one IncN plasmids also carried IncF replicons. In the legend, [*] is referring to IncI1 pST3 plasmids mostly found with *bla*_CTX-M-1_, but in one case it was found with *bla*_CTX-M-15_. [**] is referring to IncF F2:A-:B- mostly found with *bla*_CTX-M-14_, but in three cases was found with *bla*_CTX-M-15_ and three cases were found with *bla*_CTX-M-1_. NT: non-typeable plasmid; pST: plasmid sequence type; FAB: IncF FAB formula.


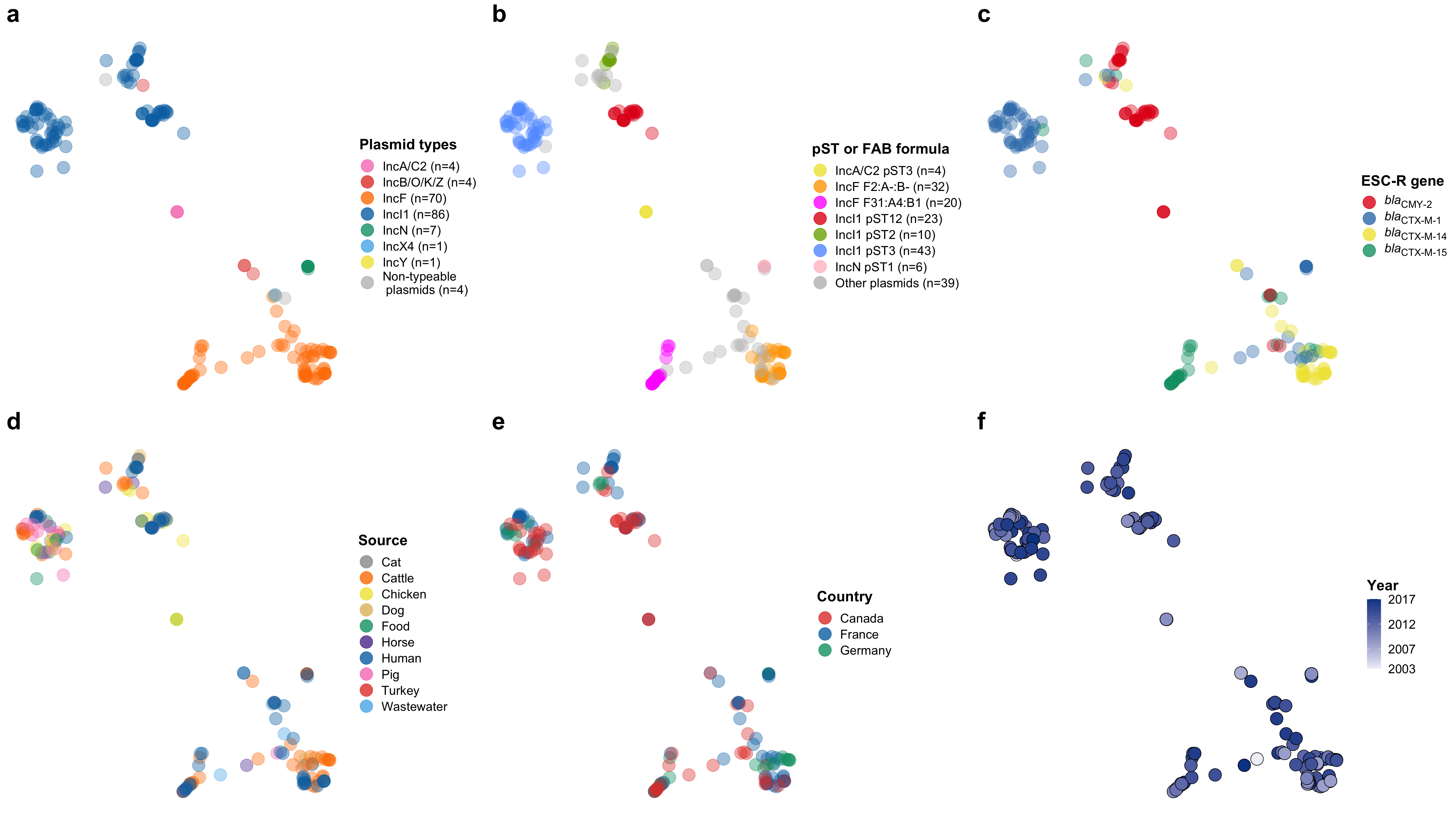


**Supplementary Figure S3. Gene content (plasmidome) network for 177 ESC-R plasmids carrying *bla*_CTX-M-1_ (n=62), *bla*_CTX-M-15_ (n=33), *bla*_CMY-2_ (n=45) and *bla*_CTX-M-14_ (n=37).** Each dot represents a plasmid, each of which is coloured by: **a)** plasmid type, **b)** plasmid sequence type (pST) or FAB formula, **c)** ESC-R gene, **d)** source, **e)** country, and **f)** year. “Other plasmids” category represents the non-typeable plasmids, IncB/O/K/Z, IncY, InX4 and those plasmids that do not belong to a main pST or FAB formula. For panel A to E a colour opacity of 0.4 was used to visualise the overlapping of the data points, while in panel F a gradient colour was used linked with the year of the isolates as shown in the scale bar. ESC: extended-spectrum cephalosporin resistance.


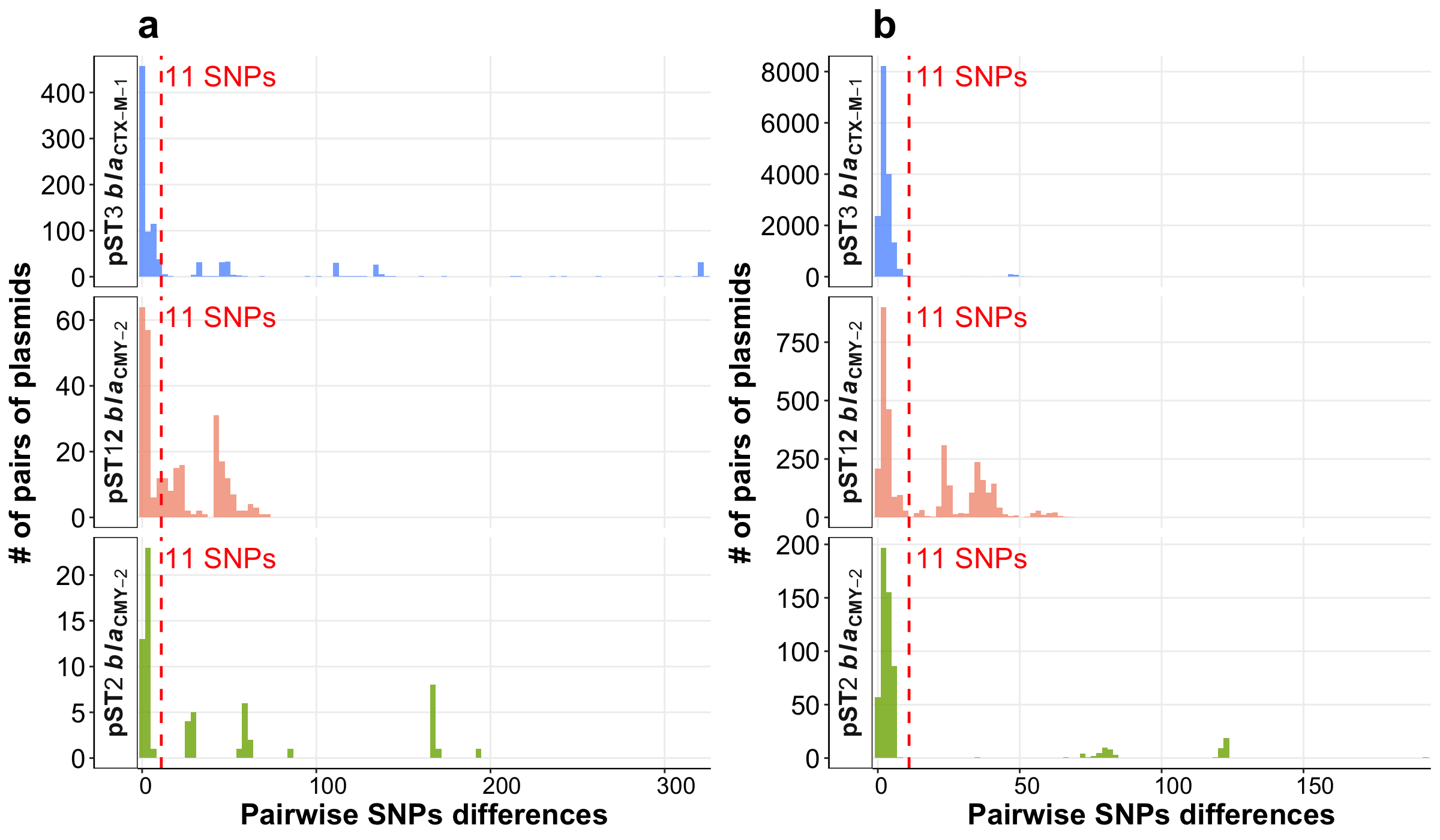


**Supplementary Figure S4. Single nucleotide polymorphism (SNP) divergence within main IncI1 plasmid subtypes. a)** SNP pairwise differences within each main IncI1 plasmid subtype: pST3 *bla*_CTX-M-1_, pST12 *bla*_CMY-2_ and pST12 *bla*_CMY-2_ from the contiguous plasmid dataset, and **b)** from the plasmid sequences recovered from the larger collection by the short-read mapping approach.


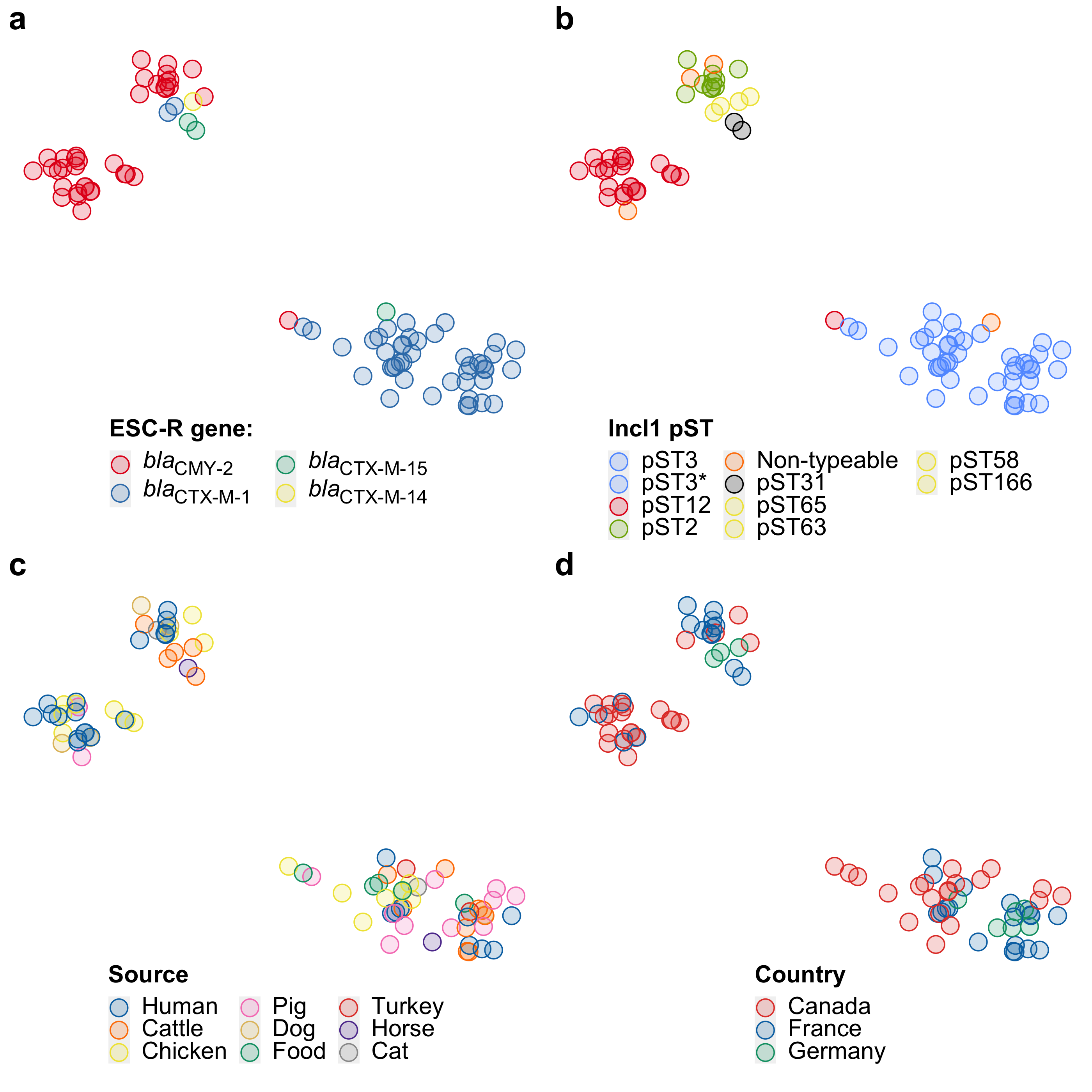


**Supplementary Figure S5. Gene content network estimated by the t-SNE algorithm using the gene presence/absence matrix from IncI1 plasmids (n=86). a)** Colouring according to the ESC-R gene, **b)** plasmid sequence type (pST), **c)** source and **d)** country.


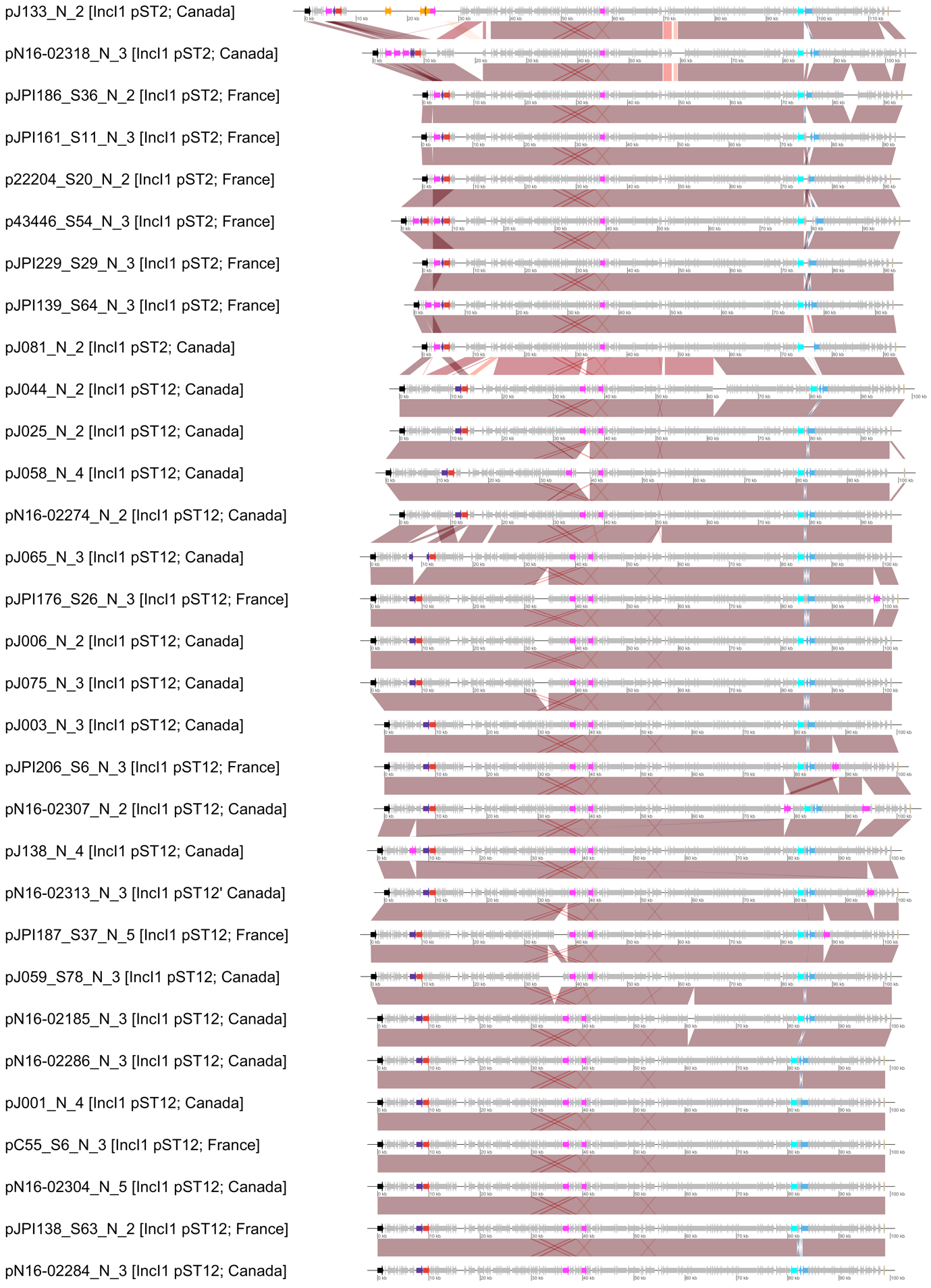


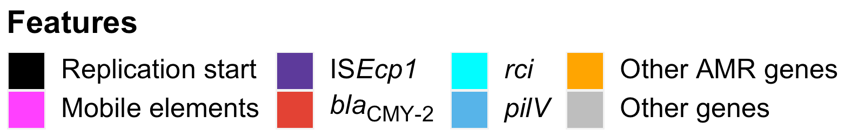


**Supplementary Figure S6.** Comparison of complete epidemic plasmids IncI1 pST2 and IncI1 pST12 harbouring *bla*_CMY-2_. On the left side is the plasmid label and within brackets is the plasmid subtype and country of origin. On the right side is the representation of the complete plasmid sequences, where homologous regions are indicated in dark red (% identity between 82% to 100%). Genes are indicated by a square, with arrowheads showing the direction of transcription; target genes are coloured as indicated in the legend.


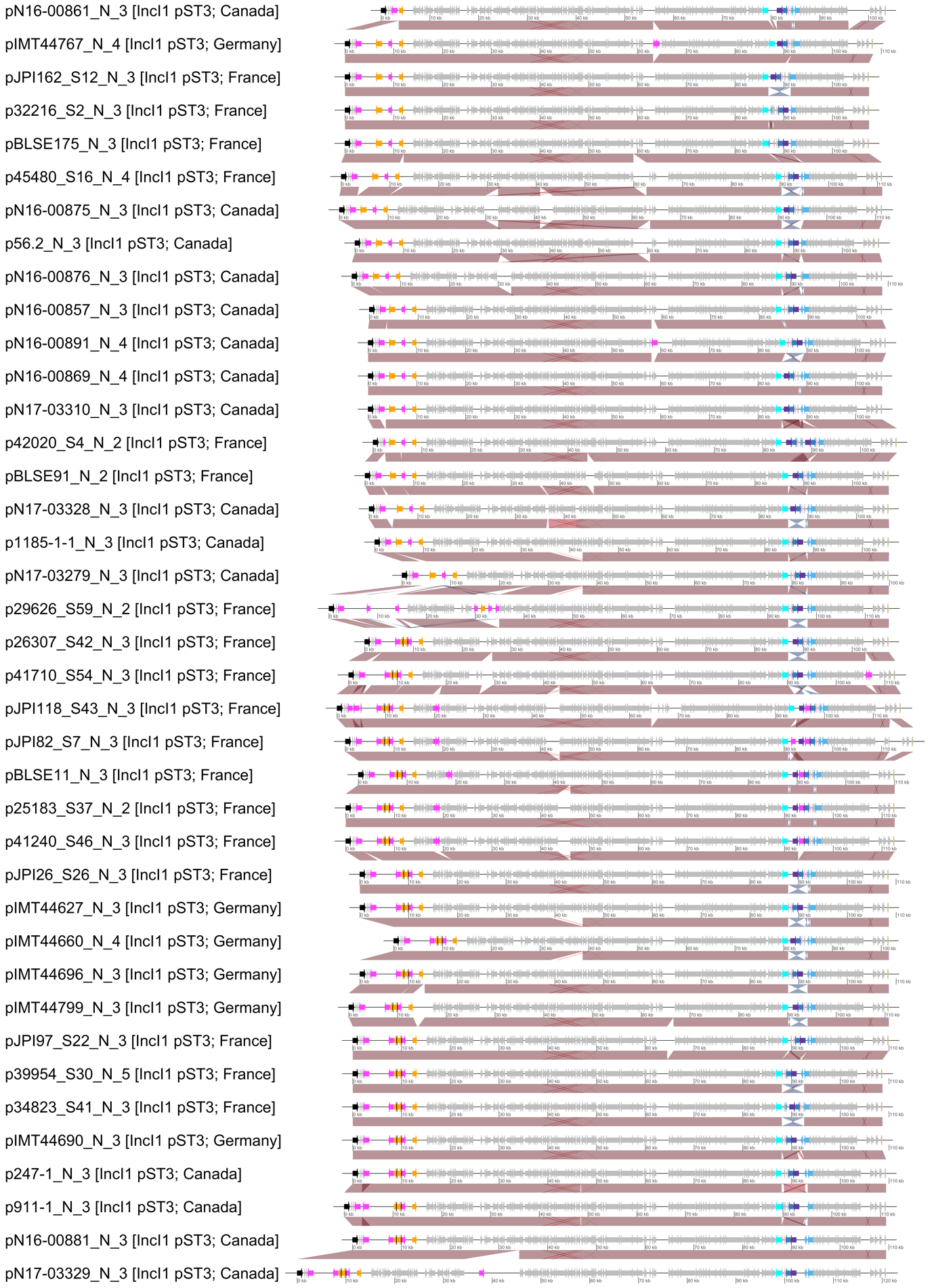


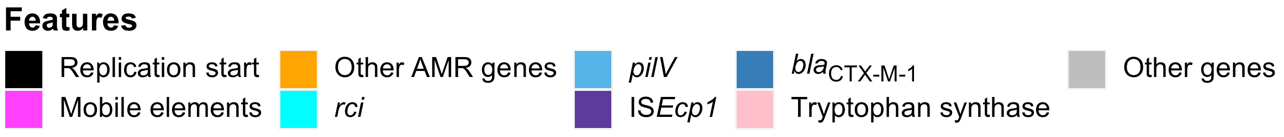


**Supplementary Figure S7.** Comparison of complete epidemic plasmid IncI1 pST3 harbouring *bla*_CTX-M-1_. On the left side is the plasmid label and within brackets is the plasmid subtype and country of origin. On the right side is the representation of the complete plasmid sequences, where homologous regions are indicated in dark red (% identity between 82% to 100%). Genes are indicated by a square, with arrowheads showing the direction of transcription; target genes are coloured as indicated in the legend.


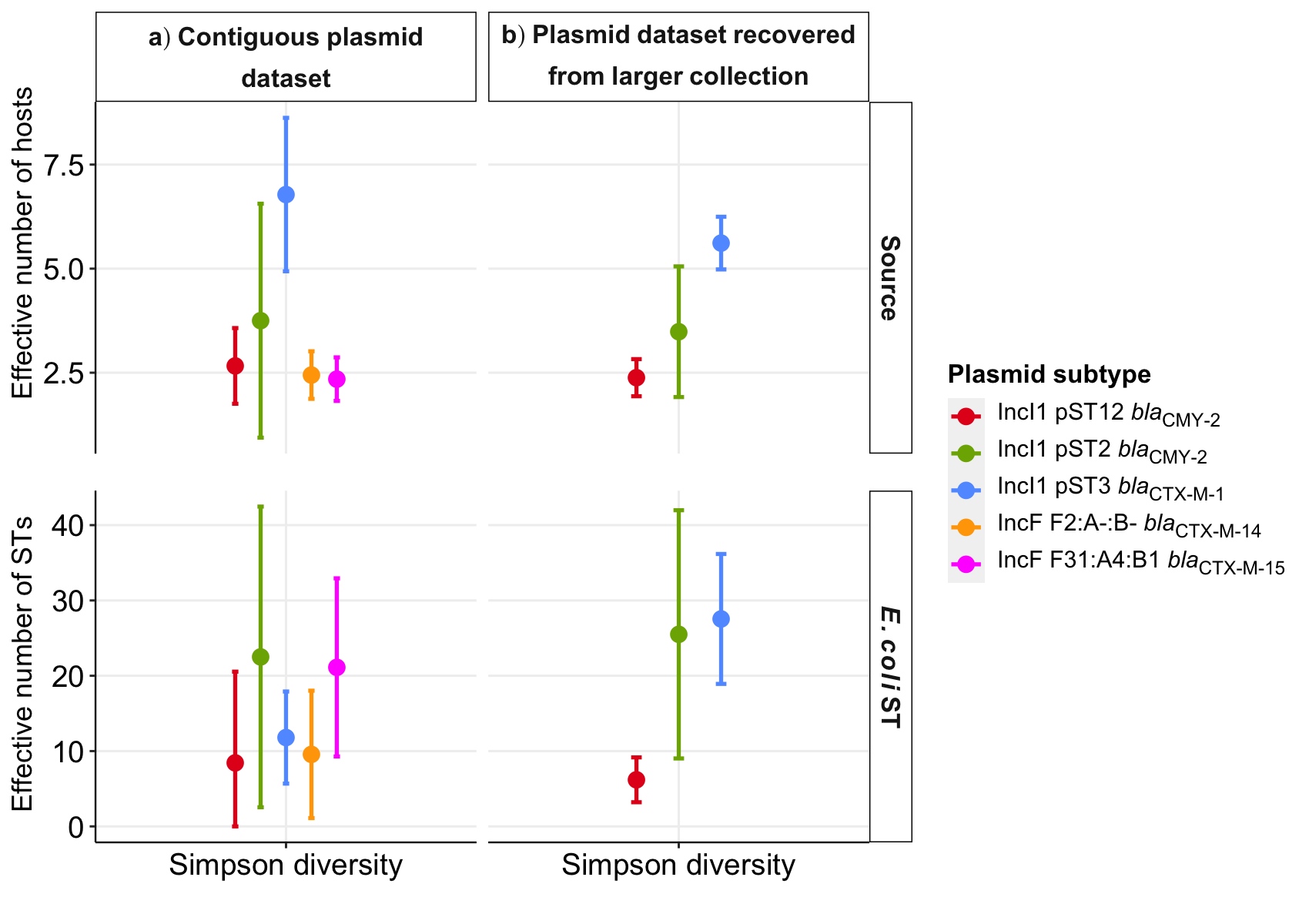


**Supplementary Figure S8. Simpson diversity estimates for source and *E. coli* ST for the main plasmid subtypes from two datasets: a)** contiguous plasmid dataset (n=121) obtained by a combination of long-read and short-read data, and **b)** plasmid sequences (n=298) recovered from the larger collection (short-read mapping approach using a reference plasmid). Effective number of sources and STs are plotted along with 95% confidence intervals. pST: plasmid sequence type.


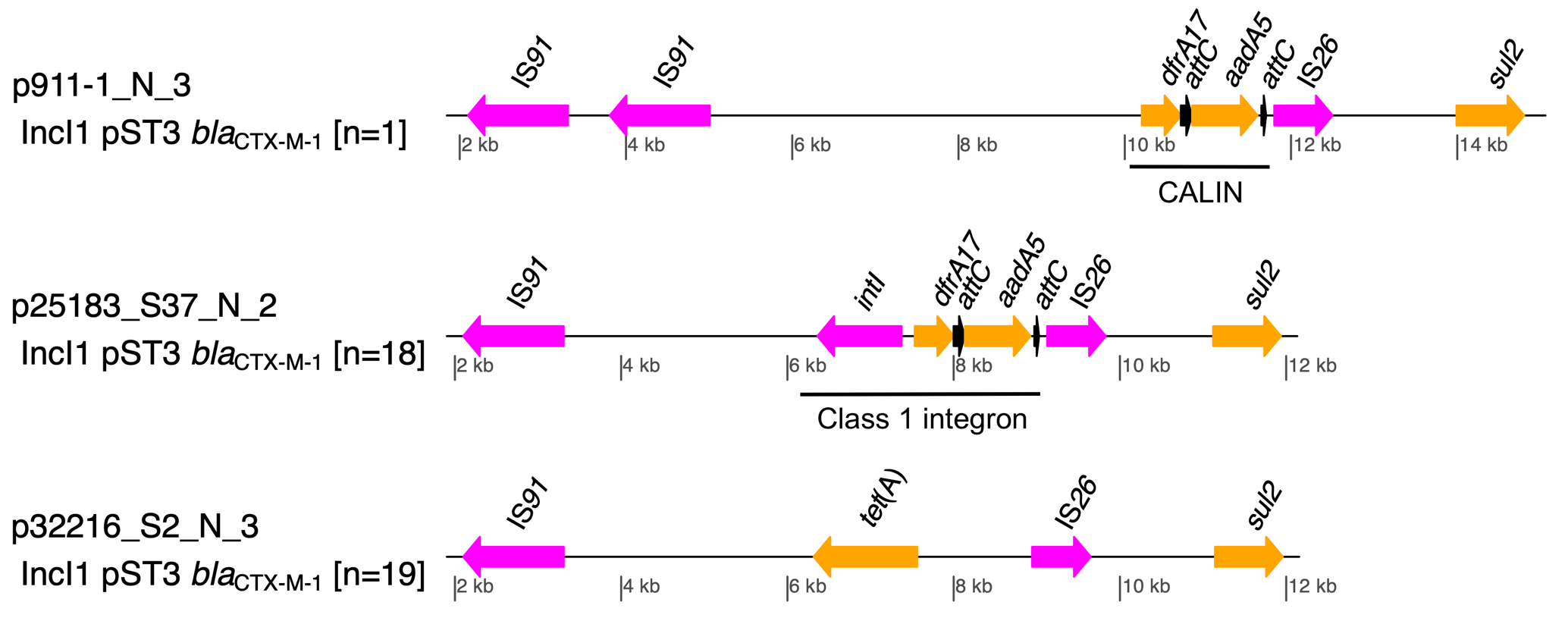


**Supplementary Figure S9. Genetic elements associated with *dfrA17-aadA5* cassette array, *sul2* and *tet*(A) in IncI1 pST3 *bla*_CTX-M-1_ plasmid subtype (n=38).** *dfrA17*-*aadA5* cassette array was found in class 1 integrons and CALIN cassettes, and *sul2* was associated with IS*26* and *tet*(A) with IS*91*. These AMR profiles are those commonly found in co-occurrence with *bla*_CTX-M-1_ in IncI1 pST3 plasmids. pST: plasmid sequence type; CALIN: cluster of *attC* sites lacking integron-integrases.

**
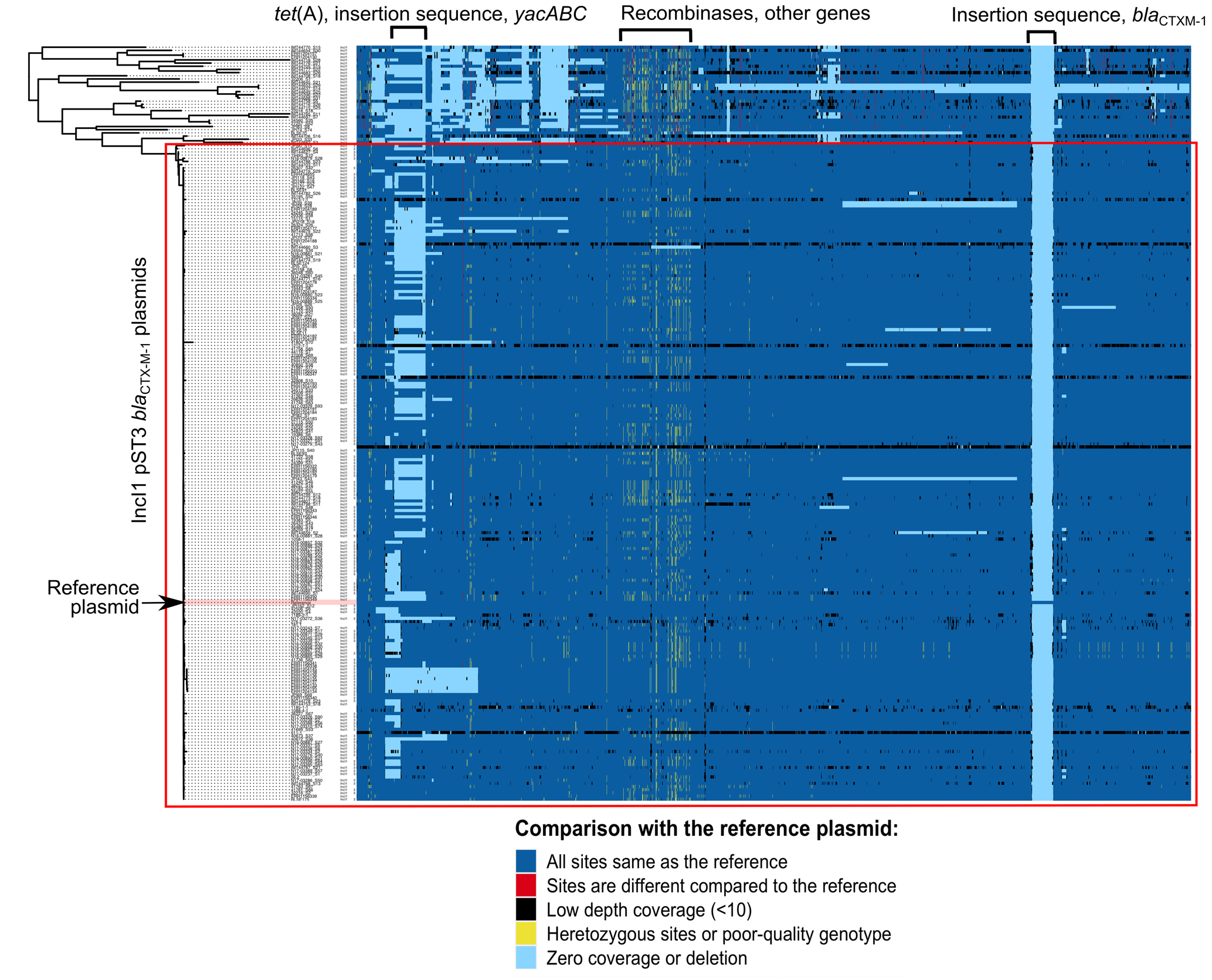
**

**Supplementary Figure S10. Mapping of the short-reads for 237 samples against the reference IncI1 pST3 *bla*_CTX-M-1_.** The red box indicates those isolates (n=182) where the plasmids belong to pST3, thus the plasmid recovery was 77% (182/237). In the top of the heatmap is indicated the regions where indels and heterozygous were observed.


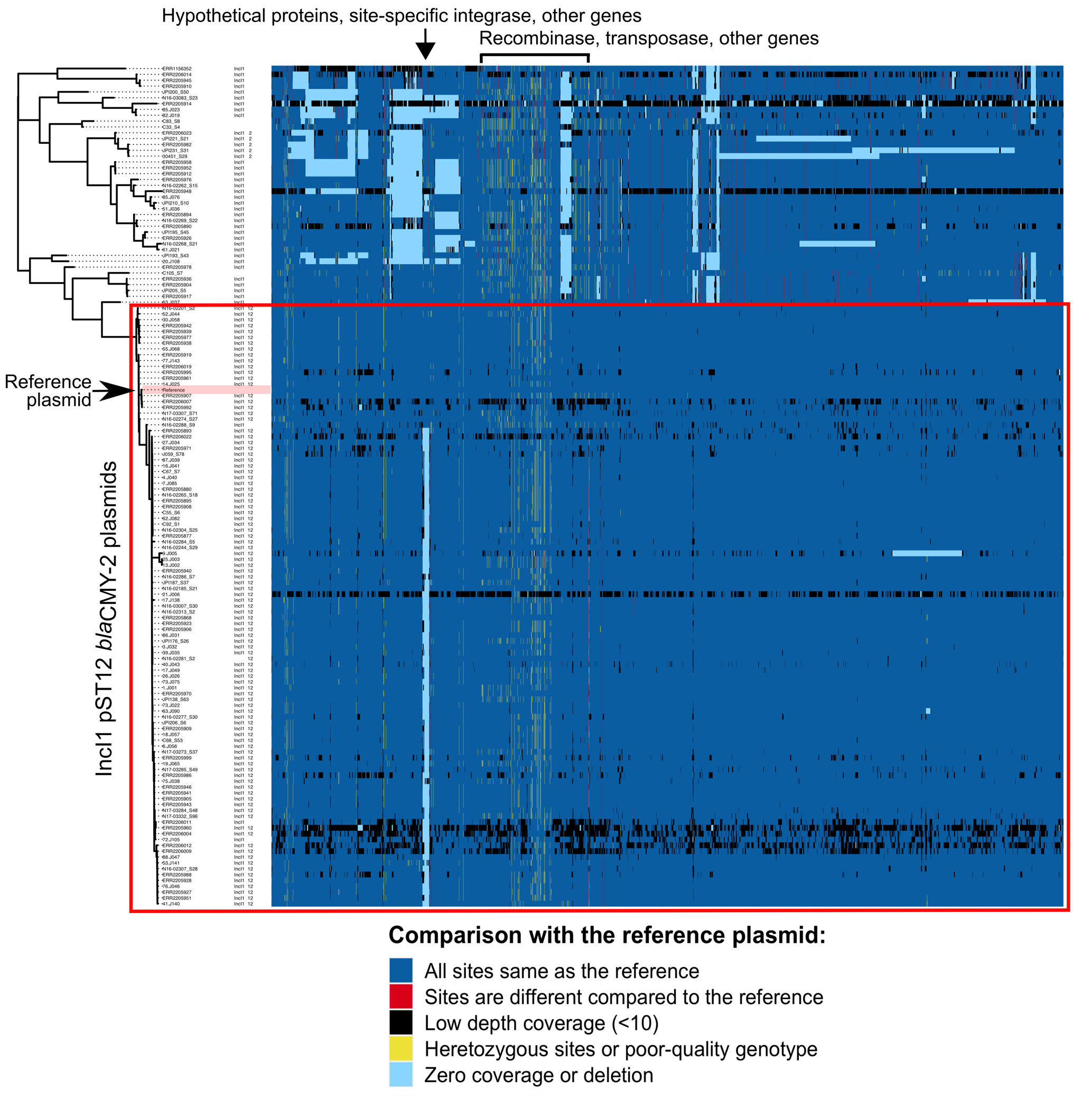


**Supplementary Figure S11. Mapping of the short-reads for 143 samples against the reference IncI1 pST12 *bla*_CMY-2_.** The red box indicates those isolates (n=82) where the plasmids belong to pST12, thus the plasmid recovery was 57% (82/143). In the top of the heatmap is indicated the regions where indels and heterozygous were observed.


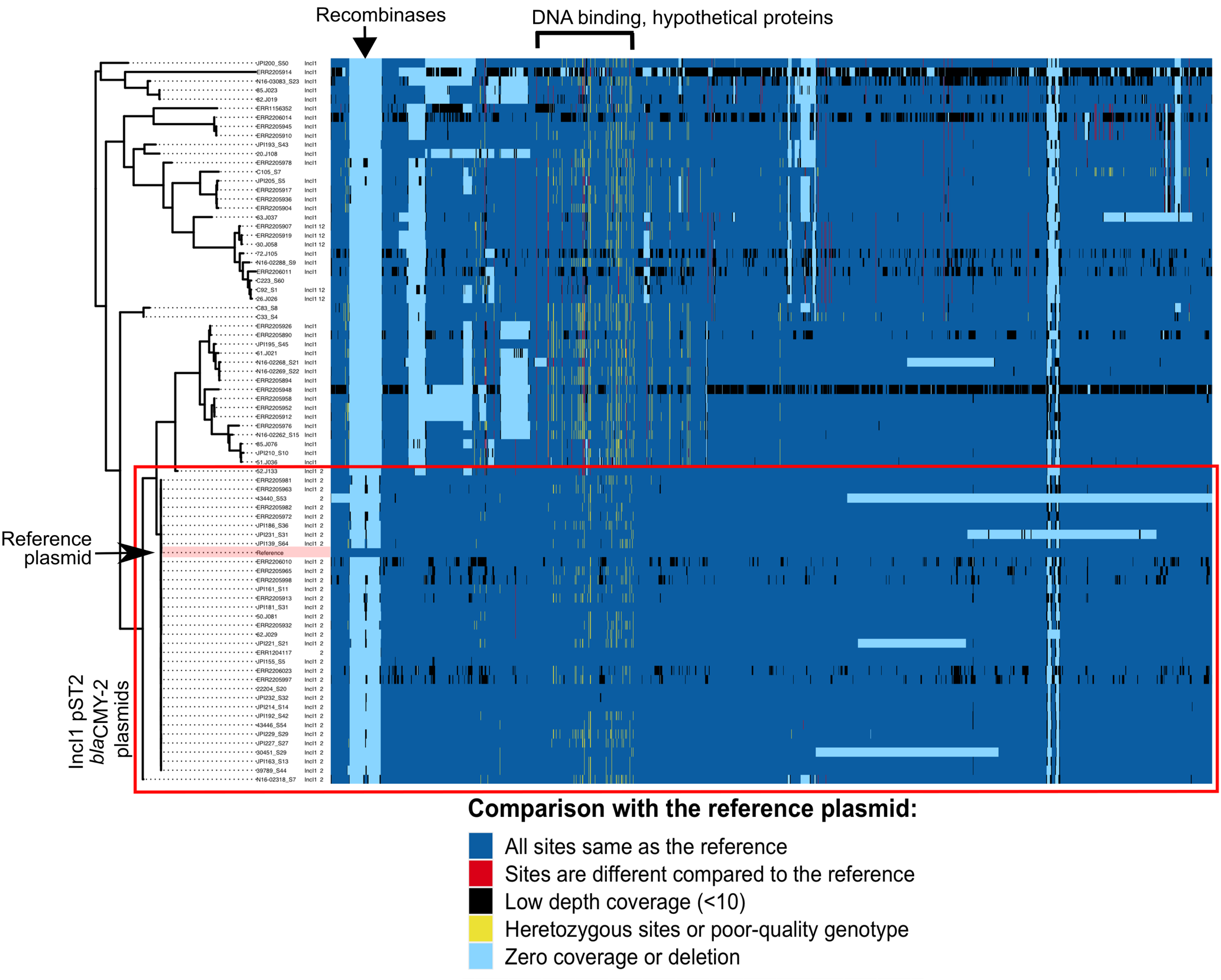


**Supplementary Figure S12. Mapping of the short-reads for 79 samples against the reference IncI1 pST2 *bla*_CMY-2_.** The red box indicates those isolates (n=34) where the plasmids belong to pST2, thus the plasmid recovery was 43% (34/79). In the top of the heatmap is indicated the regions where indels and heterozygous were observed.

**
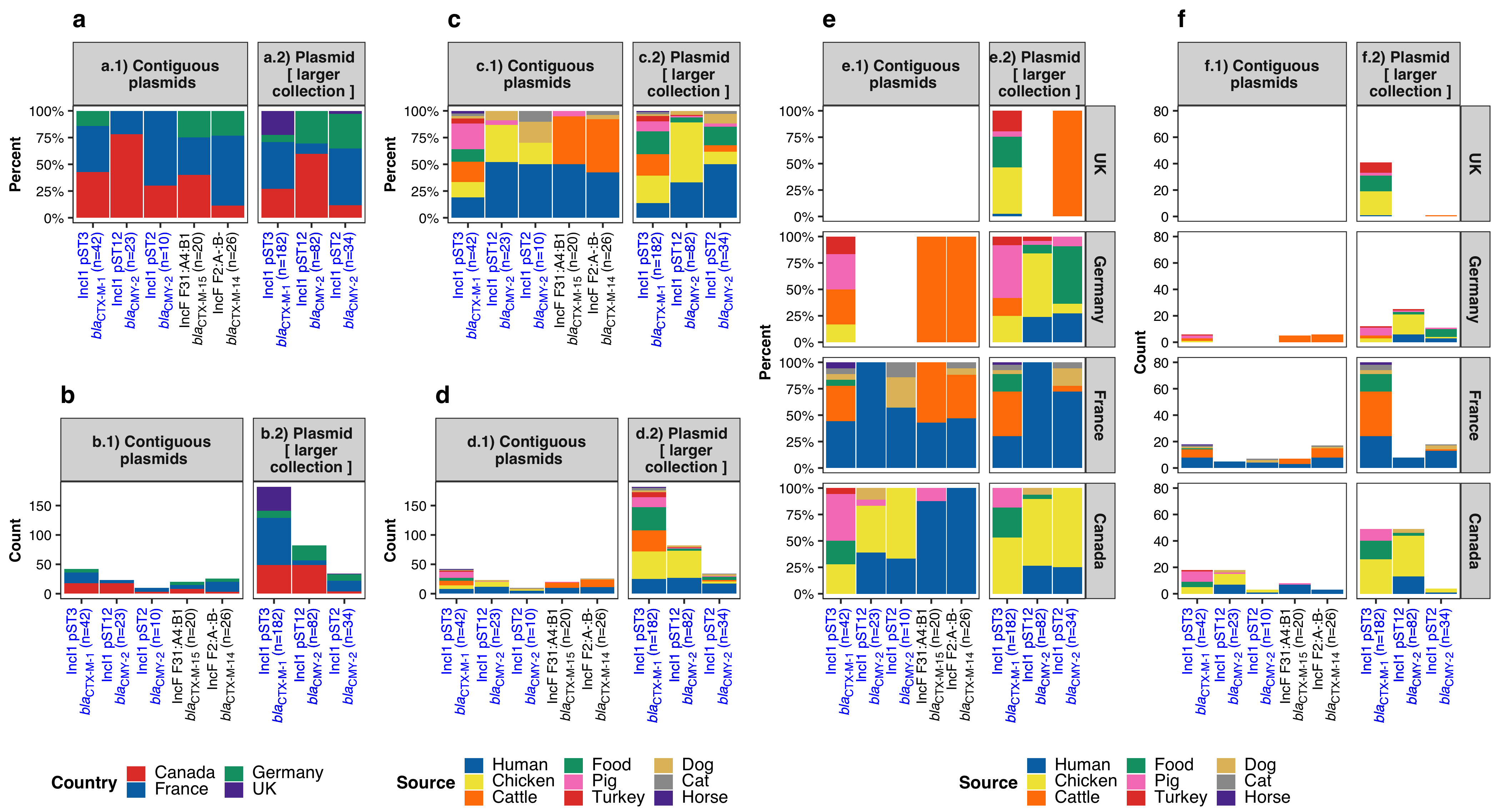
**

**Supplementary Figure S13. Proportion and number of geographical origins and source within each epidemic plasmid subtype. a)** Proportion and **b)** number of the geographical origin for the main plasmid subtypes. Bar colours are linked with the country **c)** Proportion and **d)** number of the source for the main plasmid subtypes. Bar colours are linked with the source. **e)** Proportion and **f)** number of the source by country for the main plasmid subtypes. Bar colours are linked with the source. The numbers represented in these plots were from two datasets: 1) contiguous plasmid dataset (n=121) and 2) plasmids recovered from the larger collection through the short-read mapping approach (n=298). In this latter dataset, only IncI1 epidemic plasmids were recoverable from the larger collection. pST: plasmid sequence type.


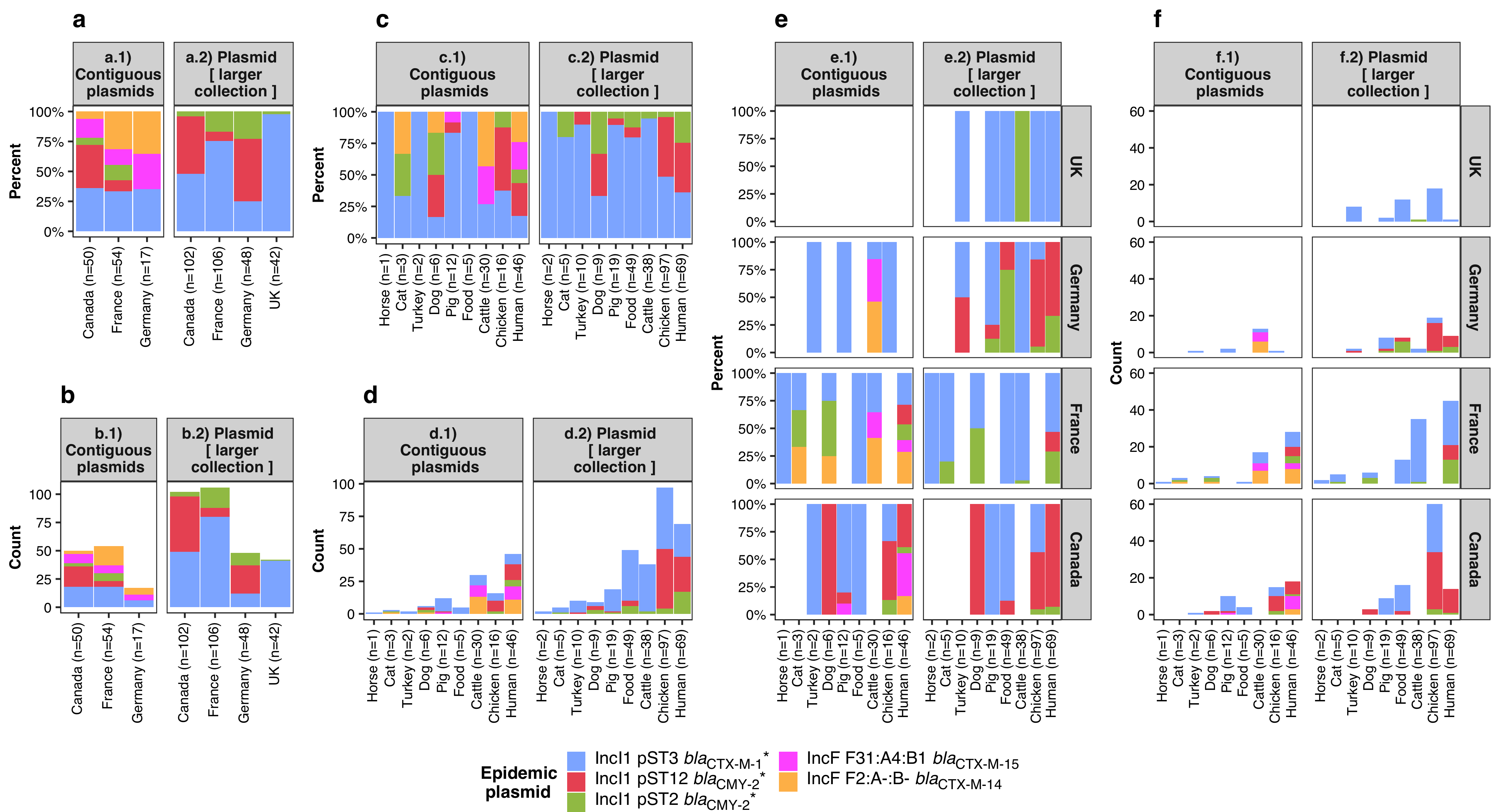


**Supplementary Figure S14. Distribution of the epidemic plasmids by country and source. a)** Proportion and **b)** number of epidemic plasmids by country. **c)** Proportion and **d)** number of epidemic plasmids by source. **e)** Proportion and **f)** number of epidemic plasmids by source and source. Bar colours are linked with the epidemic plasmids. The numbers represented in these plots were from two datasets: 1) contiguous plasmid dataset (n=121); and 2) plasmid recovered from the larger collection through short-read mapping approach (n=298).*Only IncI1 epidemic plasmids were recovered from the larger collection. pST: plasmid sequence type.


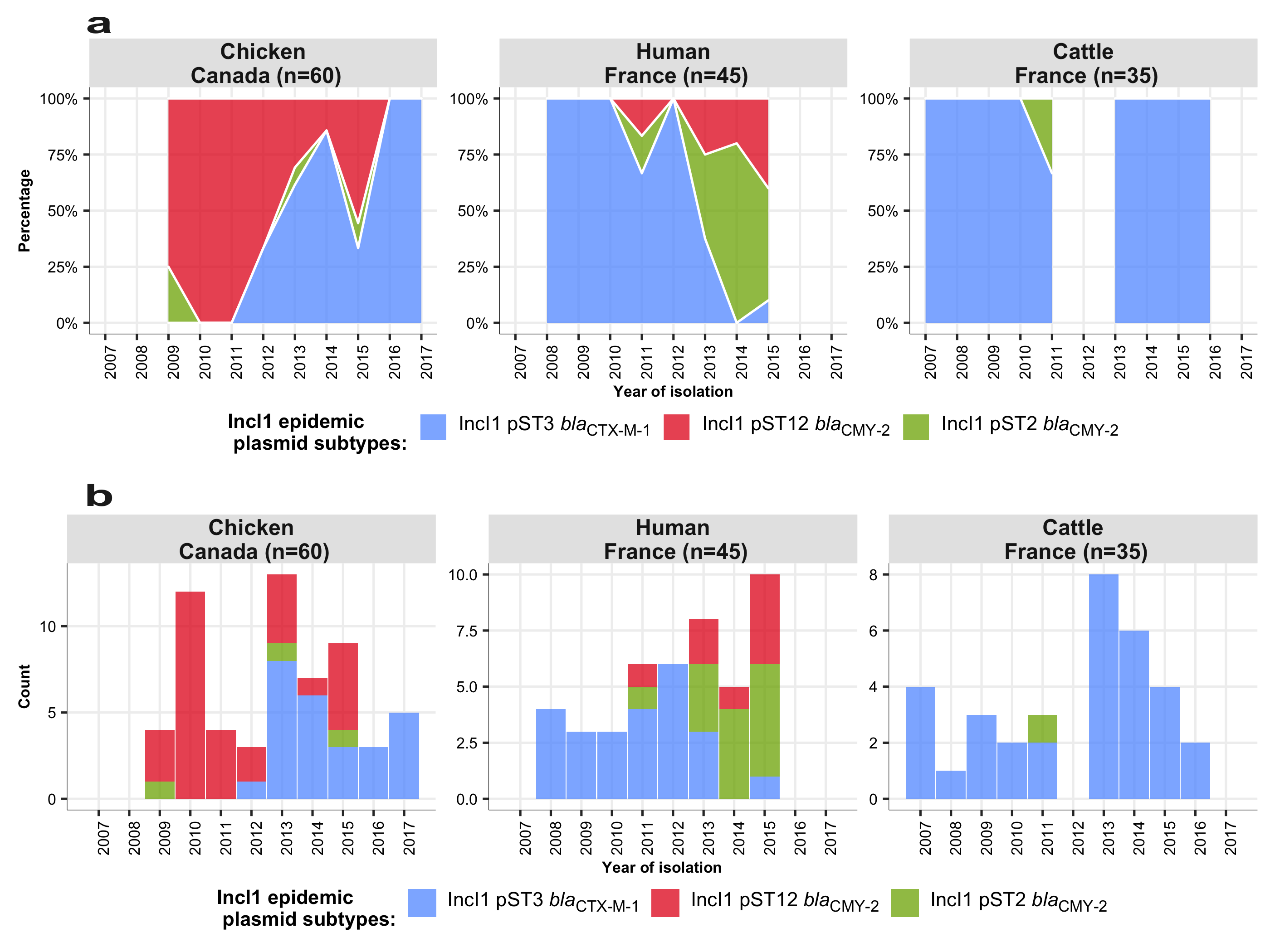


**Supplementary Figure S15. Temporal trends of IncI1 epidemic plasmids recovered from the larger collection (by short-read mapping approach) by compartment (country + source). a)** Trend in percentage and **b)** count for main IncI1 subtypes (pST3 *bla*_CTX-M-1_, pST12 *bla*_CMY-2_ and pST2 *bla*_CMY-2_) recovered from the larger collection through the short-read mapping approach. Only main compartments (source + country) with n>30 are represented. Bars are coloured by main IncI1 plasmid subtypes. pST: plasmid sequence type.


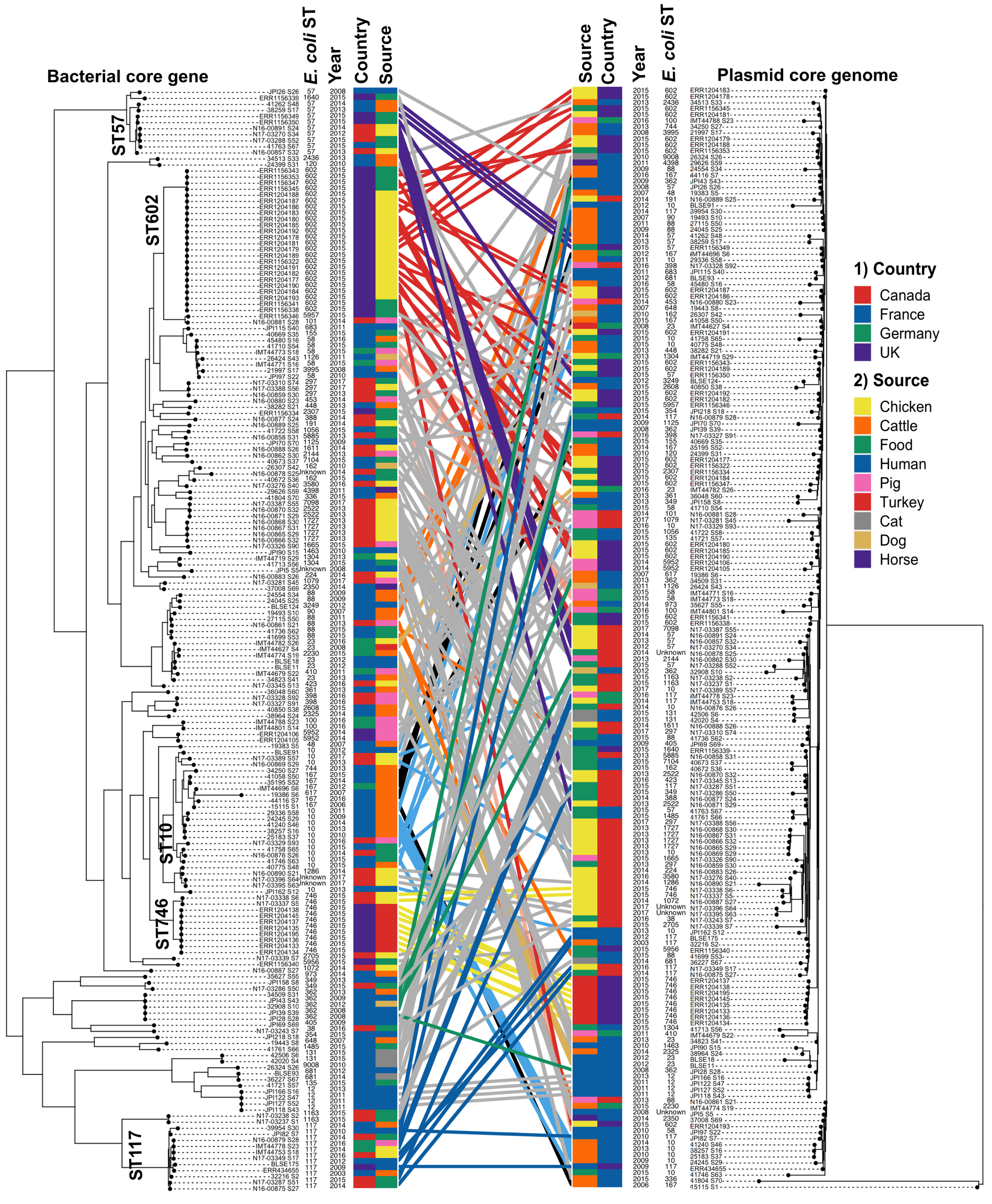


**Supplementary Figure S16. Tanglegram comparing phylogenetic trees using bacterial core genes and core genome of plasmids IncI1 pST3 *bla*_CTX-M-1_.** The maximum likelihood trees were constructed using the bacterial core gene (left) and the core genome for IncI1 pST3 *bla*_CTX-M-1_ (right) from 182 isolates. The same isolates in each tree are connected by lines between tips in the trees. The lines were coloured by the *E. coli* ST, where unique ST was coloured in grey while STs with more than one occurrence were coloured by different colours.


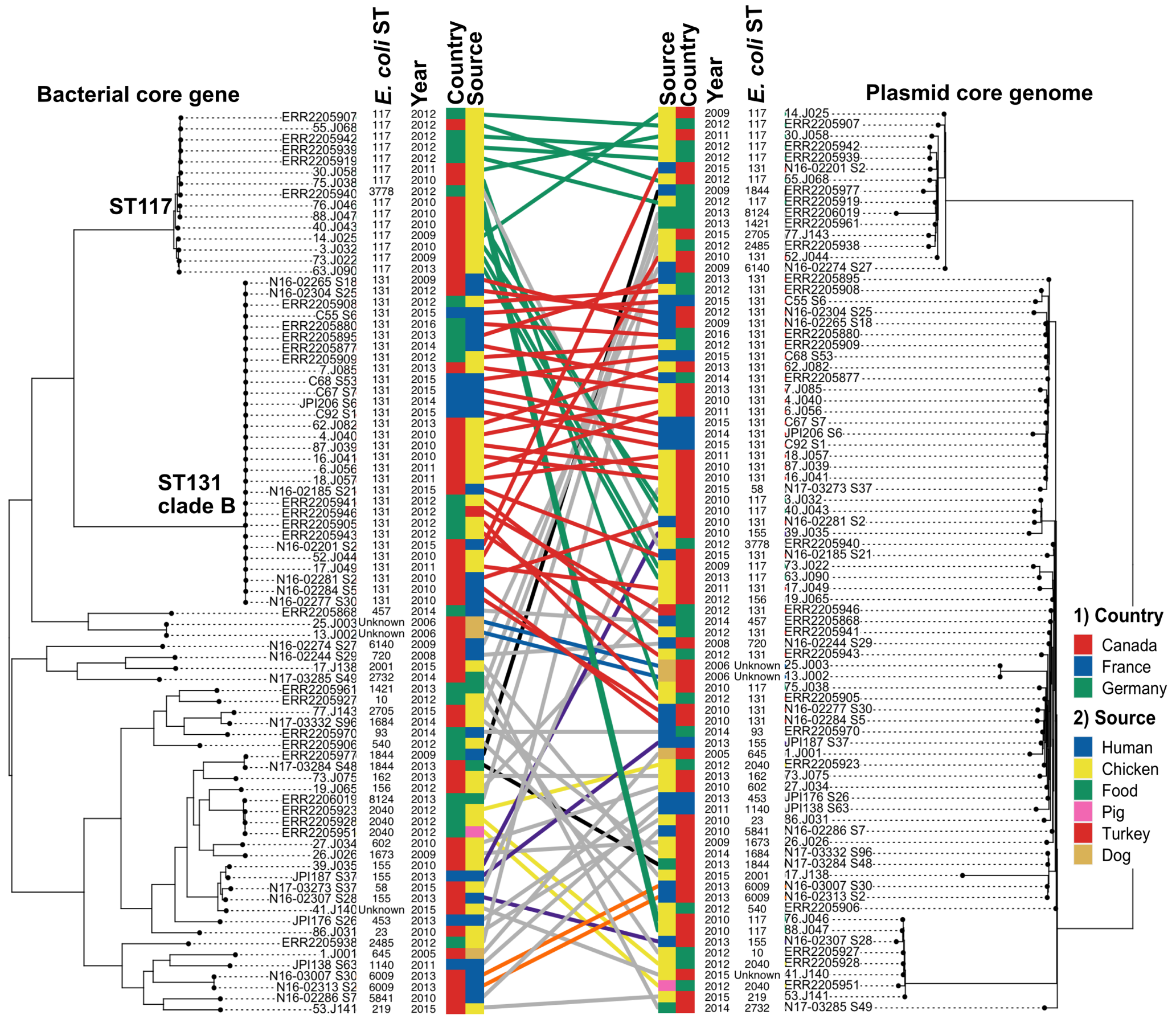


**Supplementary Figure S17. Tanglegram linking phylogenetic trees using bacterial core genes and core genome of plasmids IncI1 pST12 *bla*_CMY-2_.** The maximum likelihood trees were constructed using the bacterial core gene (left) and the core genome for IncI1 pST12 *bla*_CMY-2_ (right) from 82 isolates. The same isolates in each tree are connected by lines between tips in the trees. The lines were coloured by the *E. coli* ST, where unique ST was coloured in grey while STs with more than one occurrence were coloured by different colours.

**
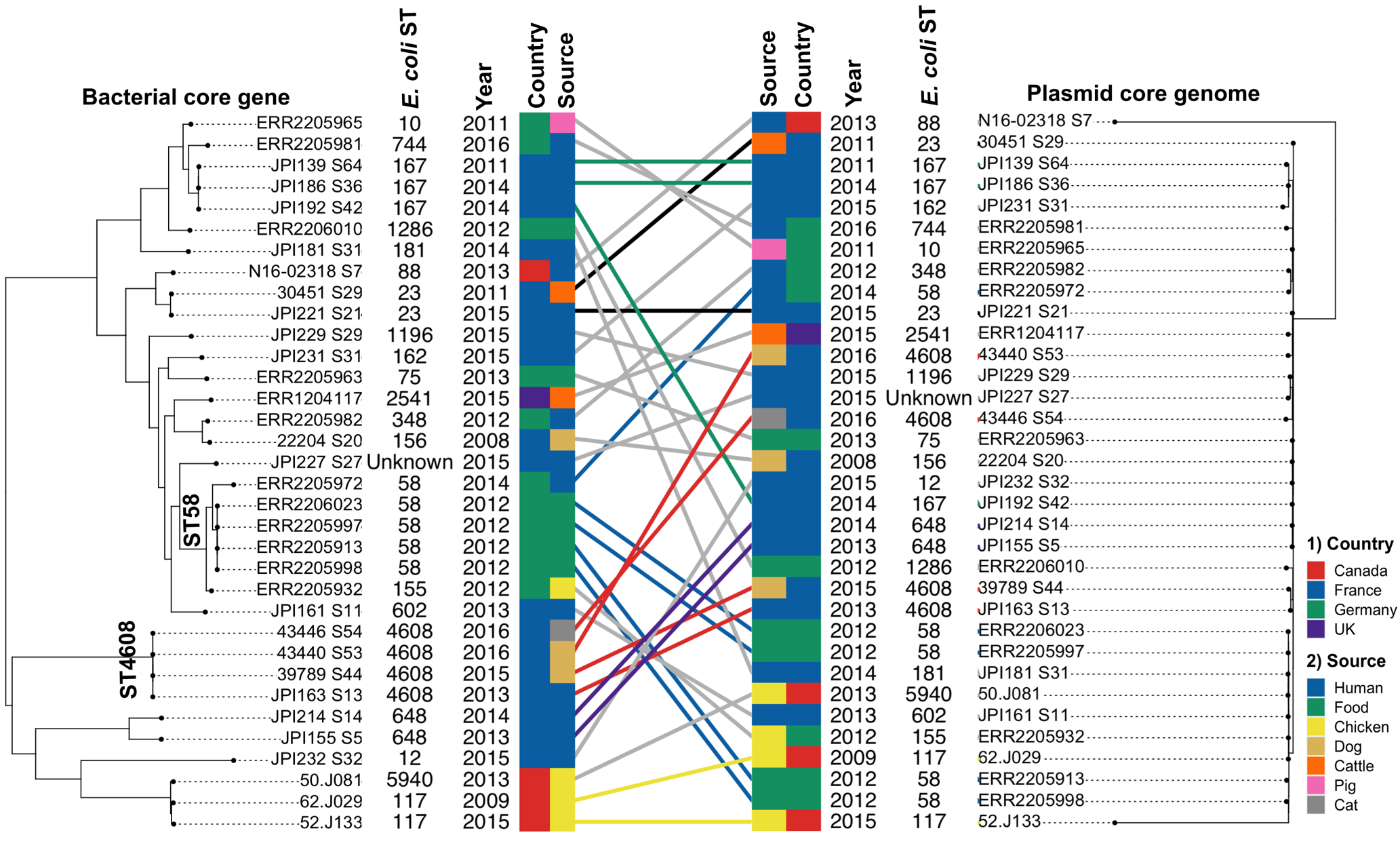
**

**Supplementary Figure S18. Tanglegram linking phylogenetic trees using core genes and core genome of plasmids IncI1 pST2 *bla*_CMY-2_.** The maximum likelihood trees were constructed using the bacterial core gene (left) and the core genome for IncI1 pST2 *bla*_CMY-2_ (right) from 34 isolates. The same isolates in each tree are connected by lines between tips in the trees. The lines were coloured by the *E. coli* ST, where unique ST was coloured in grey while STs with more than one occurrence were coloured by different colours.


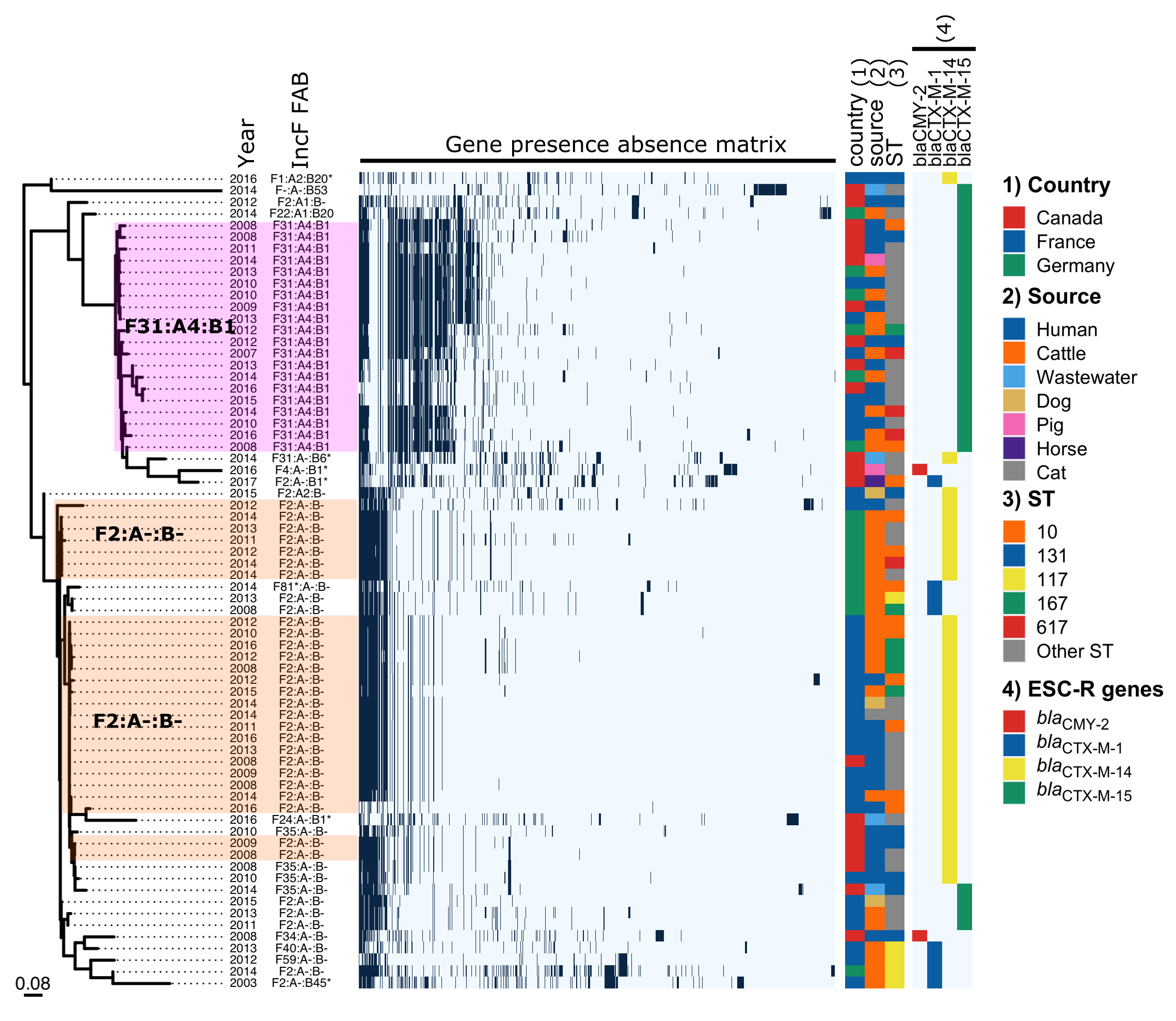


**Supplementary Figure S19. Gene presence absence tree and matrix for 70 IncF plasmid types.** Clusters of similar plasmids belonging to F31:A4:B1 *bla*_CTX-M-15_ and F2:A-:B- *bla*_CTX-M-14_ are highlighted. Year, IncF FAB formula, gene presence absence matrix (dark colour represents presence of the gene), country, source, bacterial ST and ESC-R genes are plotted against the tree. STs with <4 genomes were categorised as “Other ST”.


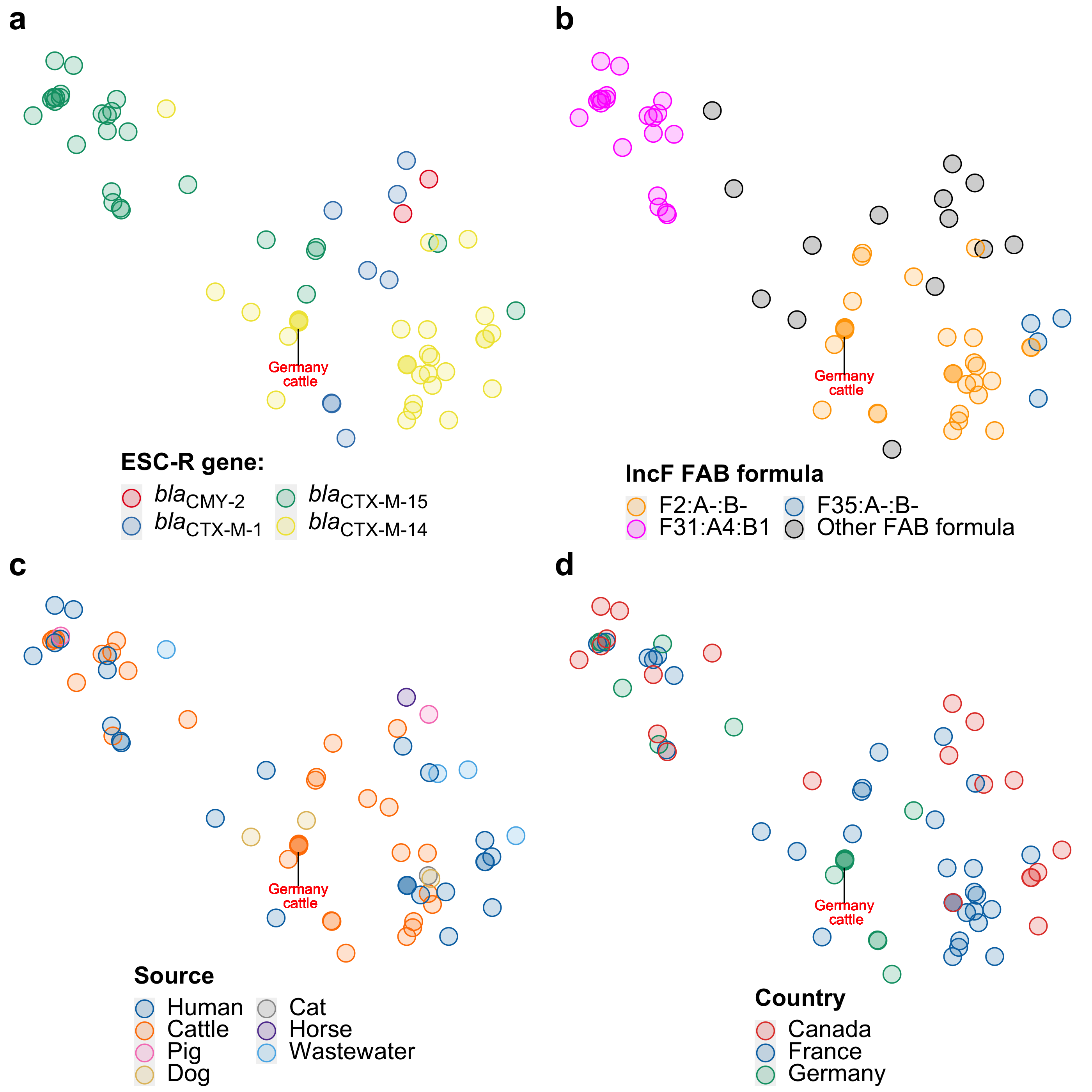


**Supplementary Figure S20. Gene content network estimated by the t-SNE algorithm using the gene presence/absence matrix from IncF plasmids (n=70). a)** Colouring according to the ESC-R gene, **b)** IncF FAB formula, **c)** source and **d)** country. In the category “Other FAB formula” are rare IncF plasmids with one occurrence (F-:A-:B53, F1:A2:B20*, F2:A-:B1*, F2:A-:B45*, F2:A1:B-, F2:A2:B-, F22:A1:B20, F24:A-:B1*, F31:A-:B6*, F34:A-:B-, F4:A-:B1*, F40:A-:B-, F59:A-:B-, and F81*:A-:B-). The text in red for the IncF F2:A-:B- *bla*_CTX-M-14_ plasmids highlight the cluster of similar plasmids in their gene content, all belonging to German cattle isolates.


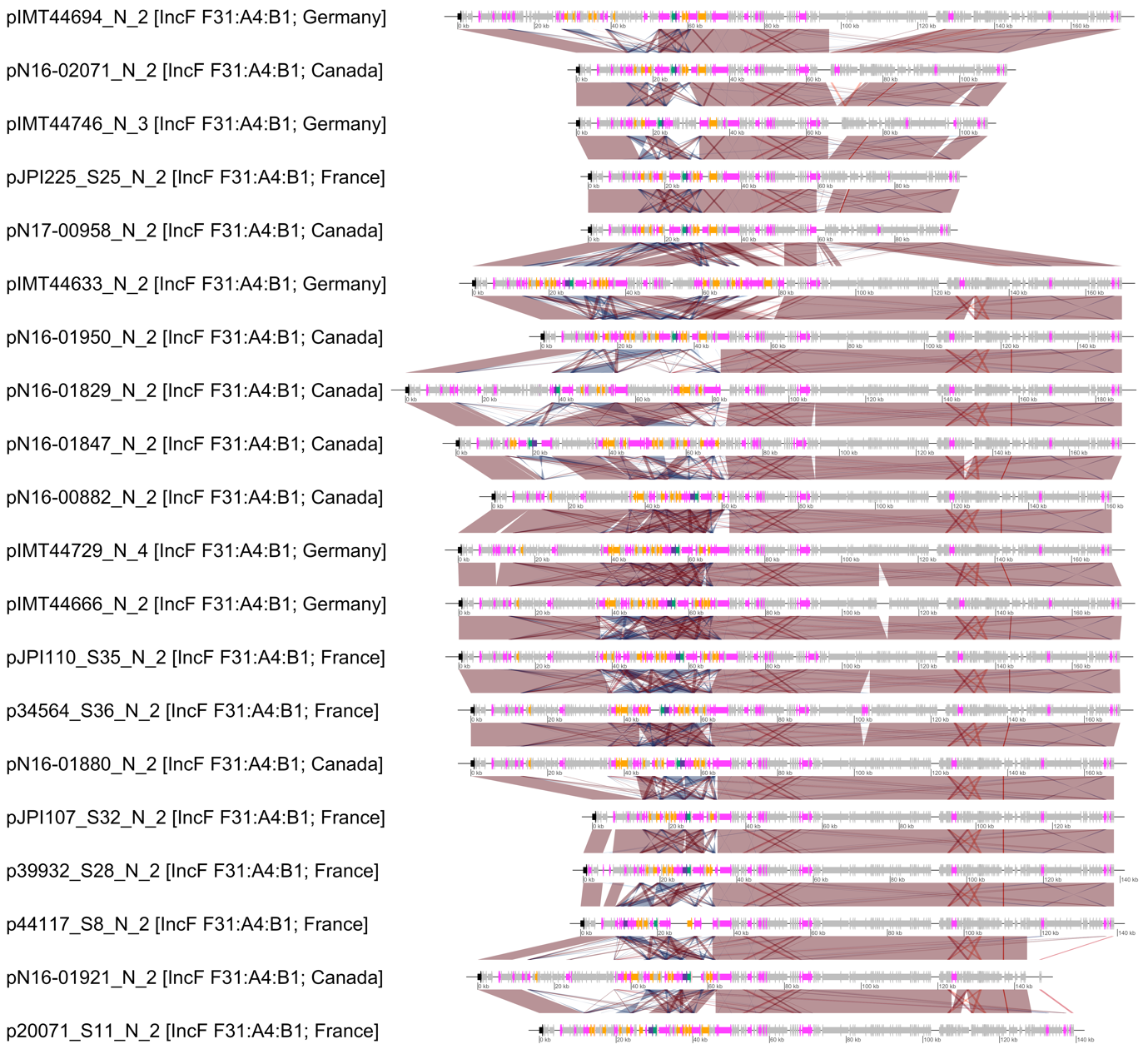


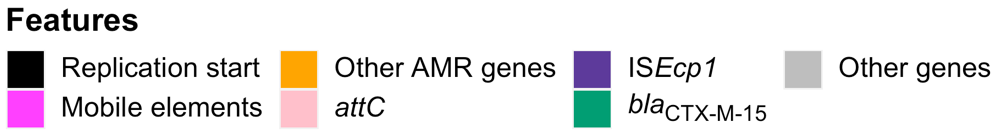


**Supplementary Figure S21.** Comparison of complete epidemic plasmid IncF F31:A4:B1 harbouring *bla*_CTX-M-15_. On the left side is the plasmid label and within brackets is the plasmid subtype and country of origin. On the right side is the representation of the complete plasmid sequences, where homologous regions are indicated in dark red (% identity between 82% to 100%). Genes are indicated by a square, with arrowheads showing the direction of transcription; target genes are coloured as indicated in the legend.


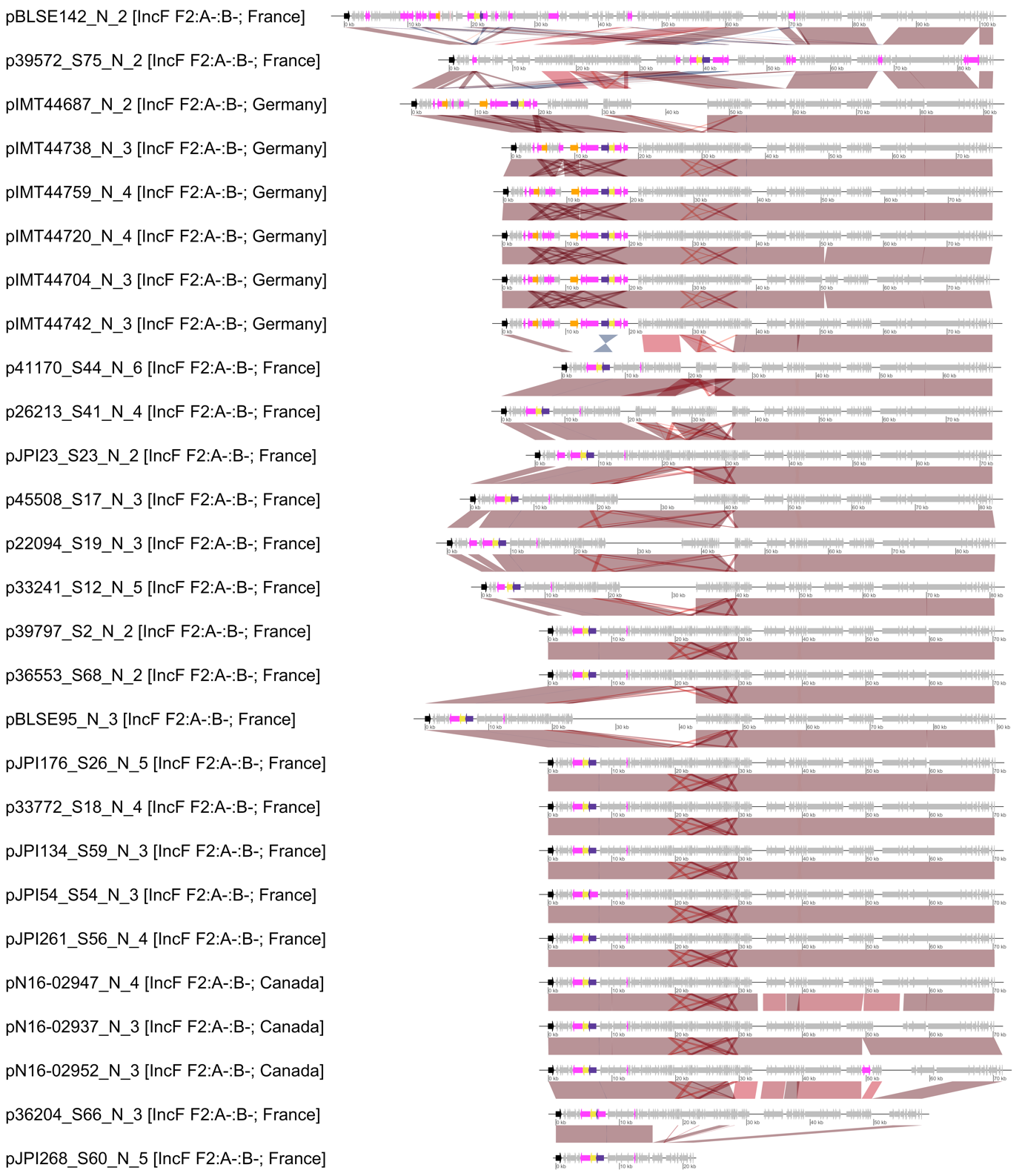


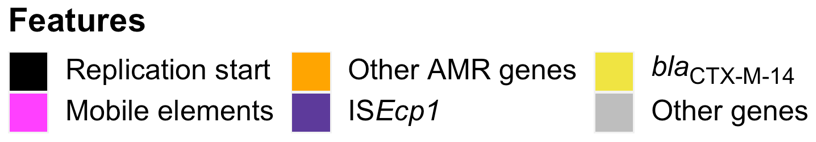


**Supplementary Figure S22.** Comparison of complete epidemic plasmid IncF F2:A-:B- harbouring *bla*_CTX-M-14_. On the left side is the plasmid label and within brackets is the plasmid subtype and country of origin. On the right side is the representation of the complete plasmid sequences, where homologous regions are indicated in dark red (% identity between 82% to 100%). Genes are indicated by a square, with arrowheads showing the direction of transcription; target genes are coloured as indicated in the legend.


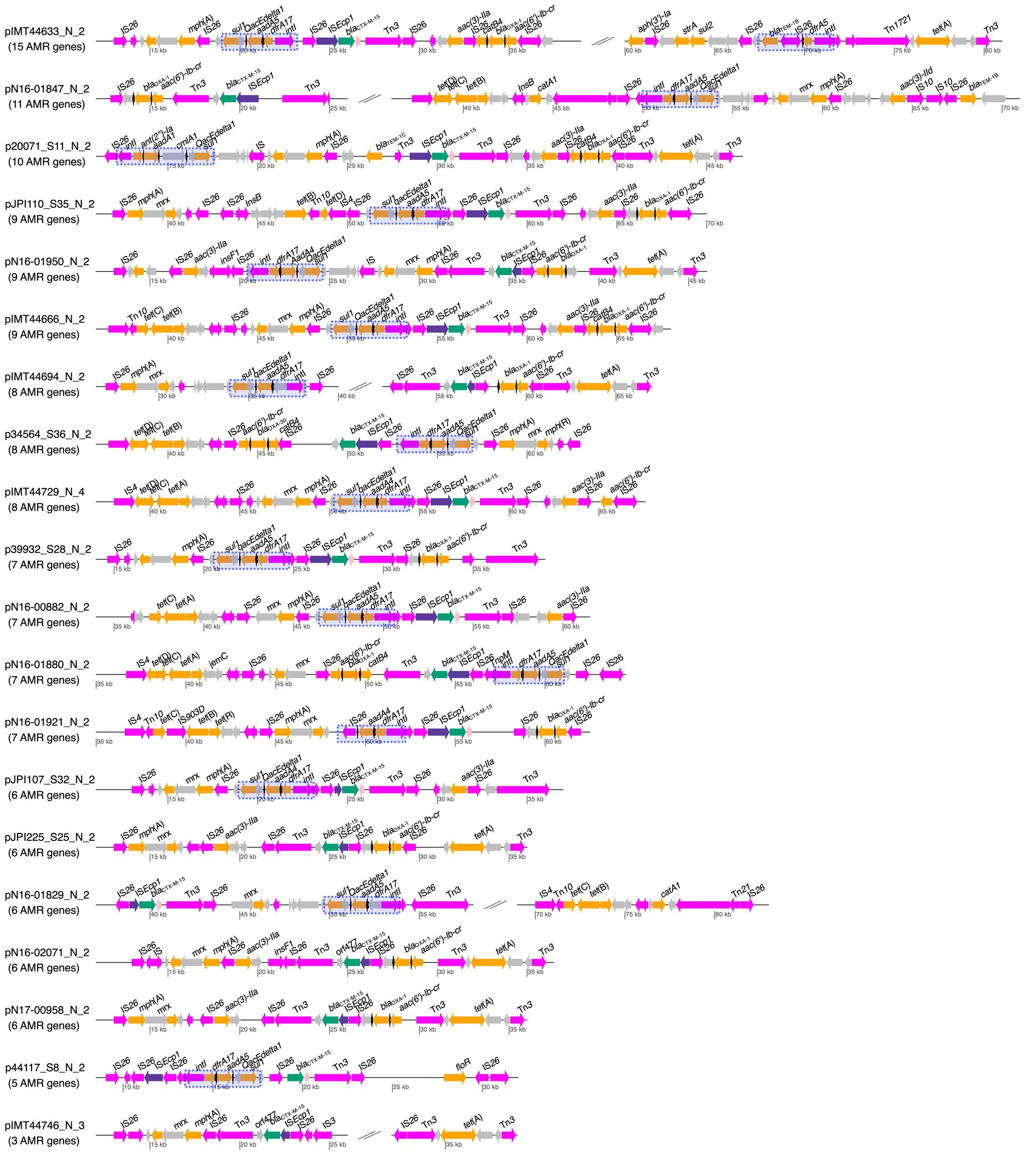


**Supplementary Figure S23. Structural features of multidrug-resistant region for 20 IncF F31:A4:B1 *bla*_CTX-M-15_ plasmid subtypes harbouring multiple AMR genes.** AMR genes are indicated by coloured boxes as follows: green, *bla*_CTX-M-15_; orange, other AMR genes. Mobile genetic elements are coloured with magenta and *attC* sites with black colour. Boxes are around the integron arrangements. The plasmids were ordered according to the number of AMR genes that they carry.

**a**


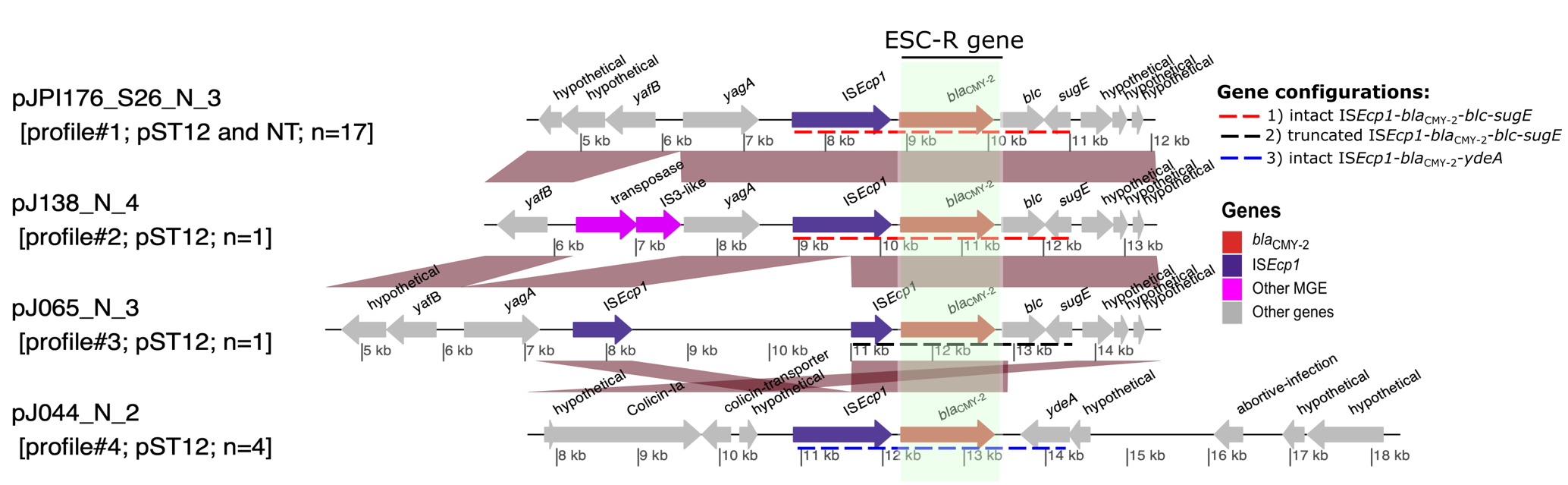


**b**


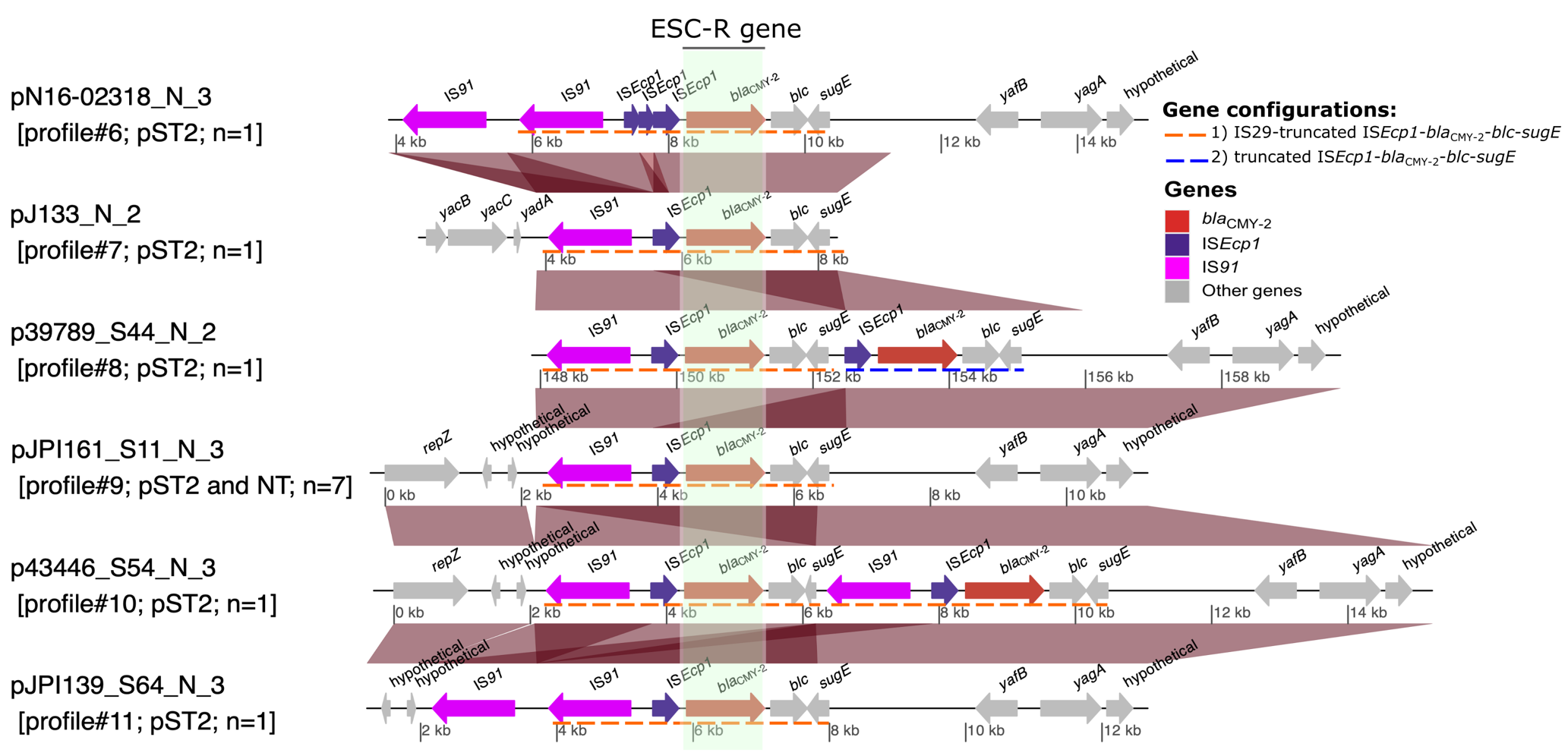


**Supplementary Figure S24. Comparison and representation of genetic environment for *bla*_CMY-2_ in plasmids belonging to the main IncI1 plasmid subtypes.** **a)** Four profiles for pST12 *bla*_CMY-2_, with three gene configurations (dashed line) **b)** Six profiles for pST2 *bla*_CMY-2_ with two gene configurations (dashed line). Gene configurations are highlighted by the dashed line with different colours. Homologous regions are shared by dark red (% identity between 82% to 100%). Genes are indicated by a square, with arrowheads showing the direction of transcription.


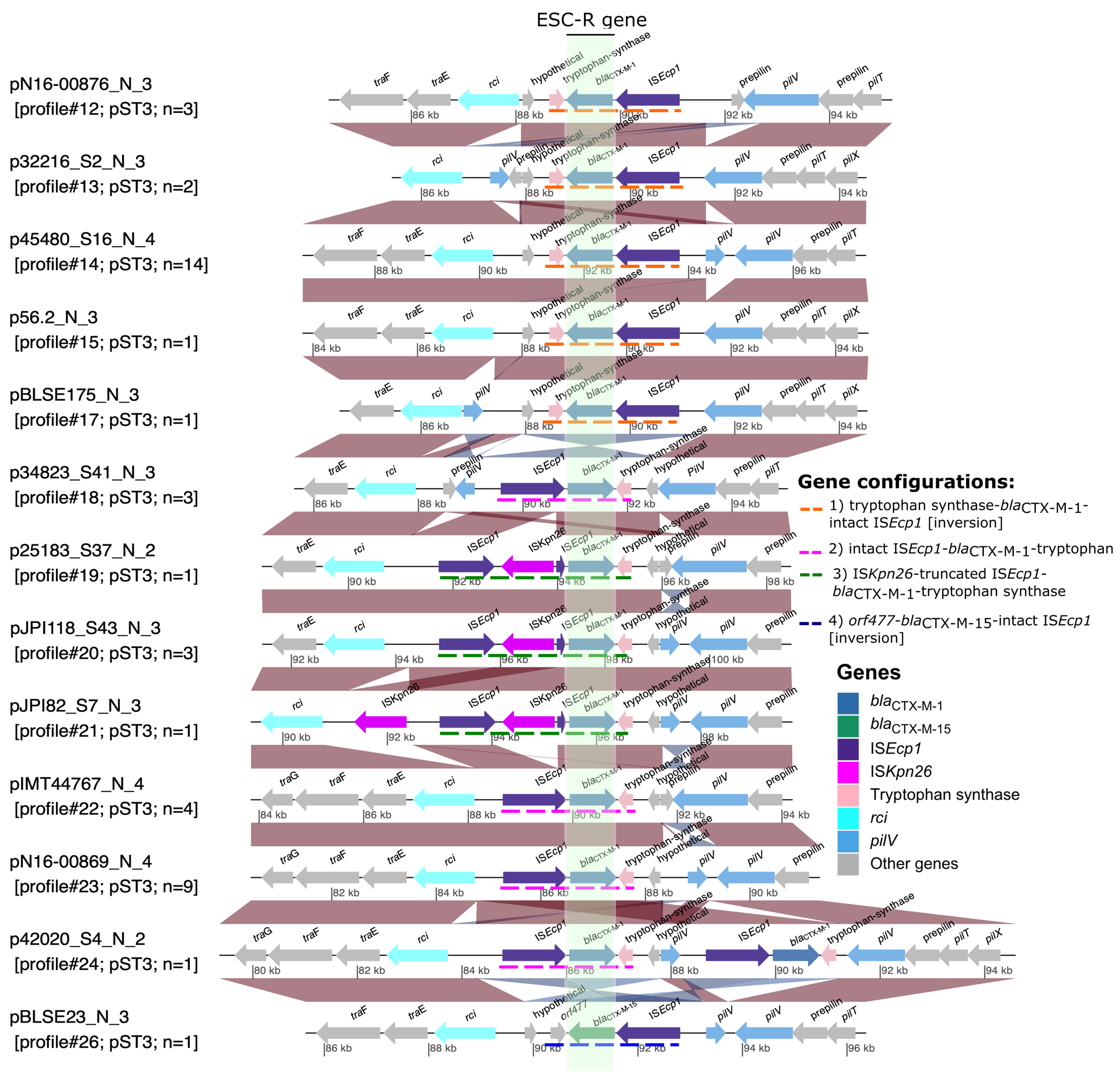


**Supplementary Figure S25. Comparison and representation of genetic environment for *bla*_CTX-M-1_ in plasmids belonging to the main IncI1 plasmid subtype.** 13 profiles for pST3 *bla*_CTX-M-1_, with three gene configurations (dashed line). Gene configurations are highlighted by the dashed line with different colours. Homologous regions are shared by dark red (% identity between 82% to 100%). Genes are indicated by a square, with arrowheads showing the direction of transcription.


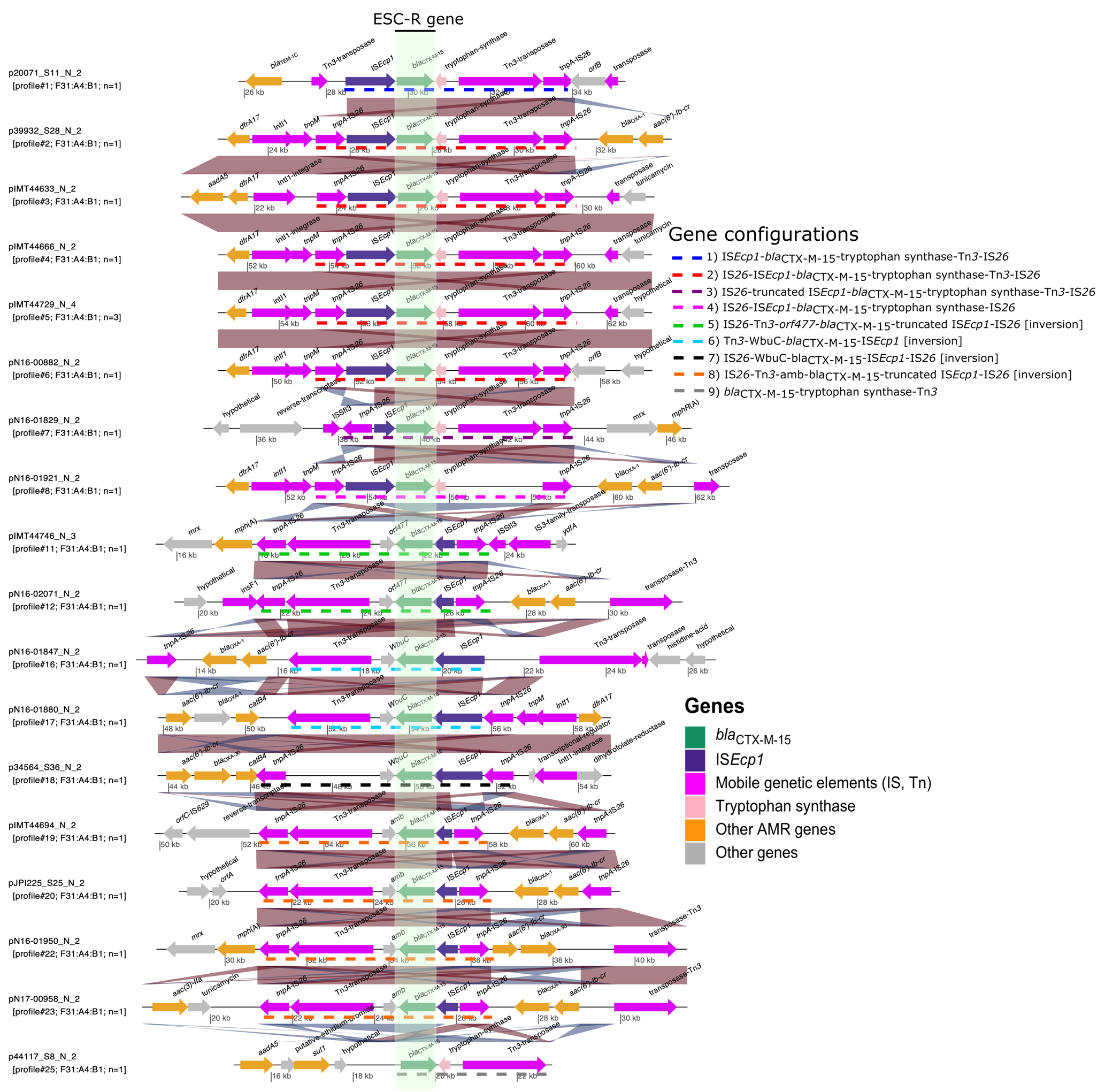


**Supplementary Figure S26. Comparison and representation of genetic environment for IncF F31:A4:B1 *bla*_CTX-M-15_ plasmid subtype (n=20).** 18 profiles for F31:A4:B1 *bla*_CTX-M-15_ with nine gene configurations (dashed line). Gene configurations are highlighted by the dashed line with different colours. Homologous regions are shared by dark red (% identity between 78% to 100%). Genes are indicated by a square, with arrowheads showing the direction of transcription.


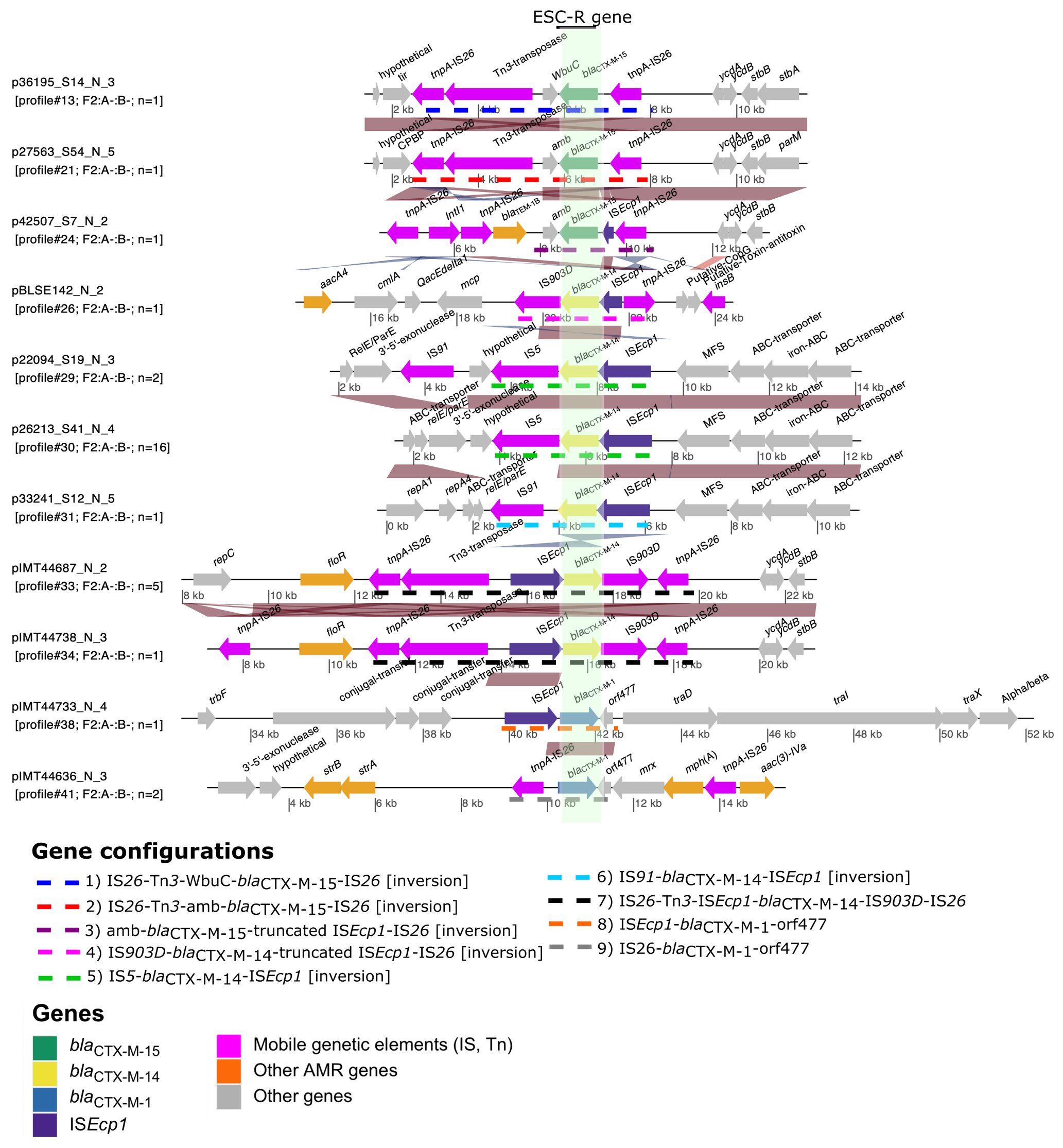


**Supplementary Figure S27. Comparison and representation of genetic environment for IncF F2:A-:B- *bla*_CTX-M-14_ plasmid subtype (n=26), F2:A-:B- *bla*_CTX-M-15_ (n=3) and F2:A-:B- *bla*_CTX-M-1_ (n=3).** 11 profiles for F2:A-:B- with nine gene configurations (dashed line). Gene configurations are highlighted by the dashed line with different colours. Homologous regions are shared by dark red (% identity between 78% to 100%). Genes are indicated by a square, with arrowheads showing the direction of transcription.


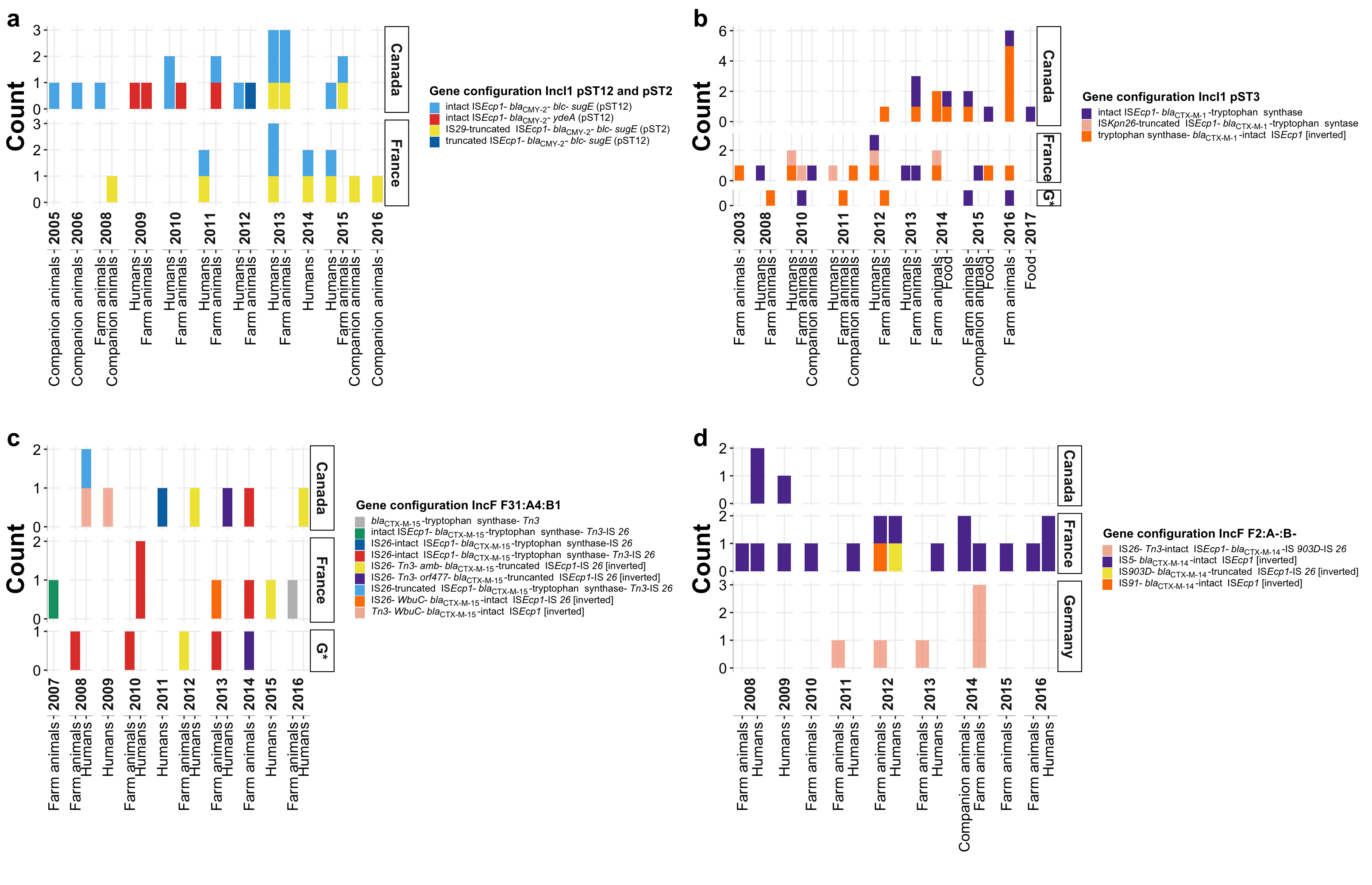


**Supplementary Figure S28. Distribution overtime of gene configurations for *bla*_CMY-2_ and *bla*_CTX-M-1_ in IncI1 and for *bla*_CTX-M-15_ and *bla*_CTX-M-14_ in IncF plasmid subtypes by country and source.** **a)** Distribution of three gene configurations for the *bla*_CMY-2_ in pST12 plasmid subtype, and one gene configuration for the *bla*_CMY-2_ in pST2. **b)** Three gene configurations for the *bla*_CTX-M-1_ in pST3 plasmid subtype. **c)** Nine gene configurations for the *bla*_CTX-M-15_ in F31:A4:B1 plasmid subtype. **d)** Four gene configurations for the *bla*_CTX-M-14_ in F2:A-:B-. G*: Germany.

# **SUPPLEMENTARY TABLES**

**Supplementary Table S1. Accession numbers of plasmids available at NCBI Nucleotide used for the annotation of contiguated plasmid sequences.**

| **NCBI accession number** | **Plasmid name** | **Isolate** | **Host bacteria** | **Reference** |
| --- | --- | --- | --- | --- |
| NC_005014.1^a^ | R64 | R64 | *Salmonella enterica* subsp. *enterica* serovar Typhimurium | Sampei *et al*. 2010^1^ |
| CP001121.1^a^ | pCVM29188_101 | CVM29188 | *Salmonella enterica* subsp. *enterica* serovar Kentucky | Fricke *et al*. 2009^2^ |
| CP012929.1^a^ | p12-4374_96 | 12-4374 | *Salmonella enterica* subsp. *enterica* Serovar Heidelberg | Labbé *et* al. 2016^3^ |
| NC_013122.1^b^ | pEK499 | EO499 | *Escherichia coli* | Woodford *et al*. 2009^4^ |
| AM886293.1^b^ | pIP1206 | 1540 | *Escherichia coli* | - |
| FJ876827.1^b^ | pKF3-140 | KF3 | *Klebsiella pneumoniae* | Zhao *et al*. 2010^5^ |
| CP009233.1^b^ | pCA08 | CA08 | *Escherichia coli* | Li *et al*. 2015^6^ |
| CP009231.1^b^ | pCA14 | CA14 | *Escherichia coli* | Li *et al*. 2015^6^ |
| CP009232.1^b^ | pCA28 | CA28 | *Escherichia coli* | Li *et al*. 2015^6^ |
| NC_019057.1^b^ | pHK01 | - | *Escherichia coli* | Ho *et al*. 2011^7^ |
| LT985271.1^b^ | RCS59_p | 661 | *Escherichia coli* | - |
| LT985213.1^b^ | RCS102_p | 708 | *Escherichia coli* | - |
| LT985277.1^b^ | RCS65_p | 522 | *Escherichia coli* | - |
| EU935738^b^ | pEK516 | D | *Escherichia coli* | Woodford *et al*. 2009^4^ |
| AP000342^b^ | R100 | 2b | *Shigella flexneri* | - |
| JX127248^b^ | pRSB225 | - | - | Wibberg *et al*. 2013^8^ |

^a^IncI1 plasmids; ^b^IncF plasmids.

**Supplementary Table S2. Number of isolates (n=19) with more than one copy of the same ESC-R gene.** 19 *E. coli* isolates had between two and three copies of the same ESC-R gene per isolate; some were located either in the plasmid or chromosome, while others were in both the plasmid and chromosome. The copy number of same ESC-R gene within the plasmid and/or chromosome per sample is shown within the brackets.

| **ESC-R gene** | **Copy number of same ESC-R and its localisation in the plasmid and/or chromosome** | **Isolate ID** | **Number of isolates** |
| --- | --- | --- | --- |
| *bla*_CMY-2_ | 2 copies; IncI1(1) and chromosome (1) | J003 | 1 |
|  | 2 copies; IncI1 (1) and non-typeable plasmid (1) | JPI186_S36 | 1 |
|  | 2 copies; IncI1(1) and IncX4 (1) | JPI229_S29 | 1 |
|  | 2 copies; IncI1 (2) | 39789_S44 and 43446_S54 | 2 |
| *bla*_CTX-M-1_ | 2 copies; IncI1 (1) and IncF (1) | 32216_S2 and IMT44733 | 2 |
|  | 2 copies; IncI1(2) | 42020_S4 | 1 |
|  | 2 copies; IncI1 (1) and chromosome (1) | 56.2 and JPI162_S12 | 2 |
|  | 3 copies; IncI1 (1) and chromosome (2) | JPI97_S22 | 1 |
| *bla*_CTX-M-14_ | 2 copies; IncF (1) and chromosome (1) | JPI176_S26*, JPI268_S60, 39572_S75 and N16-02952 | 4 |
|  | 3 copies; IncF (1) and chromosome (2) | JPI241_S41 | 1 |
|  | 2 copies; chromosome (2) | JPI29_S29 and N17-01029 | 2 |
| *bla*_CTX-M-15_ | 3 copies; chromosome (3) | JPI25_S25 | 1 |
|  | Total |  | 19 |

*The JPI176_S26 isolate also carry two different ESC-R genes: *bla*_CMY-2_ in IncI1 plasmid, and *bla*_CTX-M-14_ in IncF plasmid and in the chromosome. In addition, there are two isolates not in this table that carry two different ESC-R genes: the 247-1 isolate has a single copy of *bla*_CMY-2_ in IncF plasmid and *bla*_CTX-M-1_ in IncI1 plasmid; and JPI187_S37 has a single copy of *bla*_CMY-2_ in IncI1 plasmid and *bla*_CTX-M-2_ in IncHI2 plasmid.

**Supplementary Table S3. Number of plasmid types (n=177) and chromosome (n=30) harbouring ESC-R genes.**

| **ESC-R gene** | **Plasmids** | | **Chromosome** | |
| --- | --- | --- | --- | --- |
|  | **Plasmid type (n)** | **Total (n)** | **Bacterial ST** | **Total (n)** |
| *bla*_CMY-2_  (n=46) | IncI1 (n=37), IncA/C2 (n=4), IncF (n=2), IncX4 (n=1), non-typeable (n=1) | 45 | Unknown ST (n=1) | 1 |
| *bla*_CTX-M-1_  (n=69) | IncI1 (n=45), IncF (n=8), IncN (n=7), IncB/O/K/Z (n=1), non-typeable (n=1) | 62 | ST10 (n=3), ST117 (n=2), ST58 (n=1), ST681 (n=1) | 7 |
| *bla*_CTX-M-15_  (n=45) | IncF (n=27), IncI1 (n=3), non-typeable (n=2), IncY (n=1) | 33 | ST131 (n=8), ST10 (n=1), ST410 (n=1), ST617 (n=1), ST1485 (n=1) | 12 |
| *bla*_CTX-M-14_  (n=47) | IncF (n=33), IncB/O/K/Z (n=3), IncI1 (n=1) | 37 | ST131 (n=6), ST10 (n=1), ST38 (n=1), ST453 (n=1), ST6215 (n=1) | 10 |
| Total |  | 177 | Total | 30 |

“Non-typeable” category refers to those plasmid-identified contigs that do not carry any replicon sequence included in Plasmidfinder and MOB-suite.

**Supplementary Table S4. Number of plasmid types (n=177) and chromosome (n=30) harbouring main ESC-R genes.** Count and percentage (in brackets) of plasmid subtypes in the table was calculated by ESC-R gene and plasmid type from the *E. coli* genome collection with short and long-read data (n=192 genomes).

| **ESC-R gene** |  | **IncI1^#^** | **IncF** | **IncN** | **IncB/O/K/Z** | **IncA/C2^#^** | **IncY** | **IncX4** | **NT** | **Chr** |
| --- | --- | --- | --- | --- | --- | --- | --- | --- | --- | --- |
| ***bla*_CMY-2_ (n=46)** | **pST or FAB formula** | **n=37** | **n=2** | - | - | **n=4** | - | **n=1** | **n=1** | **n=1** |
|  | pST12^&^ | 23 (62%) | - | - | - | - | - | - | - | - |
|  | pST2^&^ | 10 (27%) | - | - | - | - | - | - | - | - |
|  | pST65 | 1 (3%) | - | - | - | - | - | - | - | - |
|  | pST-NT** | 3 (8%) | - | - | - | - | - | - | - | - |
|  | pST3 | - | - | - | - | 4 (100%) | - | - | - | - |
|  | F34:A-:B- | - | 1 (50%) | - | - | - | - | - | - | - |
|  | F4:A-:B1* | - | 1 (50%) | - | - | - | - | - | - | - |
|  |  | **IncI1^#^** | **IncF^#^** | **IncN^#^** | **IncB/O/K/Z** | **IncA/C2** | **IncY** | **IncX4** | **NT^#^** | **Chr^#^** |
| ***bla*_CTX-M-1_ (n=69)** | **pST or FAB formula** | **n=45** | **n=8** | **n=7** | **n=1** | - | - | - | **n=1** | **n=7** |
|  | pST3^&^ | 41 (91%) | - | - | - | - | - | - | - | - |
|  | pST3* | 1 (2%) | - | - | - | - | - | - | - | - |
|  | pST58 | 1 (2%) | - | - | - | - | - | - | - | - |
|  | pST63 | 1 (2%) | - | - | - | - | - | - | - | - |
|  | pST-NT** | 1 (2%) | - | - | - | - | - | - | - | - |
|  | F2:A-:B- | - | 3 (38%) | - | - | - | - | - | - | - |
|  | F2:A-:B1* | - | 1 (13%) | - | - | - | - | - | - | - |
|  | F2:A-:B45* | - | 1 (13%) |  |  |  |  |  |  |  |
|  | F40:A-:B- | - | 1 (13%) | - | - | - | - | - | - | - |
|  | F59:A-:B- | - | 1 (13%) | - | - | - | - | - | - | - |
|  | F81*:A-:B- | - | 1 (13%) | - | - | - | - | - | - | - |
|  | pST1^&^ | - | - | 7 (100%) | - | - | - | - | - | - |
|  |  | **IncI1^#^** | **IncF** | **IncN** | **IncB/O/K/Z** | **IncA/C2** | **IncY** | **IncX4** | **NT** | **Chr^#^** |
| ***bla*_CTX-M-15_ (n=45)** | **pST or FAB formula** | **n=3** | **n=27** | - | - | - | **n=1** | - | **n=2** | **n=12** |
|  | F31:A4:B1* | - | 20 (74%) | - | - | - | - | - | - | - |
|  | F2:A-:B- | - | 3 (11%) | - | - | - | - | - | - | - |
|  | F-:A-:B53 | - | 1 (4%) | - | - | - | - | - | - | - |
|  | F2:A1:B- | - | 1 (4%) | - | - | - | - | - | - | - |
|  | F22:A1:B20 | - | 1 (4%) | - | - | - | - | - | - | - |
|  | F35:A-:B- | - | 1 (4%) | - | - | - | - | - | - | - |
|  | pST3* | 1 (33%) | - | - | - | - | - | - | - | - |
|  | pST31^&^ | 2 (67%) | - | - | - | - | - | - | - | - |
|  |  | **IncI1** | **IncF** | **IncN** | **IncB/O/K/Z** | **IncA/C2** | **IncY** | **IncX4** | **NT** | **Chr^#^** |
| ***bla*_CTX-M-14_ (n=47)** | **pST or FAB formula** | **n=1** | **n=33** | - | **n=3** | - | - | - | - | **n=10** |
|  | F2:A-:B- | - | 26 (79%) | - | - | - | - | - | - | - |
|  | F35:A-:B- | - | 3 (9%) | - | - | - | - | - | - | - |
|  | F1:A2:B20* | - | 1 (3%) | - | - | - | - | - | - | - |
|  | F2:A2:B- | - | 1 (3%) | - | - | - | - | - | - | - |
|  | F24:A-:B1* | - | 1 (3%) | - | - | - | - | - | - | - |
|  | F31:A-:B6* | - | 1 (3%) | - | - | - | - | - | - | - |
|  | pST166 | 1 (100%) | - | - | - | - | - | - | - | - |
| **TOTAL** |  | **86** | **70** | **7** | **4** | **4** | **1** | **1** | **4** | **30** |

^#^No circular plasmids were obtained for eight IncI1 plasmids, and one for each IncF, IncN and IncA/C2. Also, one non-typeable plasmid was not circularisable. Five chromosome sequences were not circularisable.

^&^IncF replicons was found in six IncI1 plasmids: three in pST3 *bla*_CTX-M-1_ plasmids [F-:A-:B1, F18:A-:B1, F18:A5:B1], one in each pST2 *bla*_CMY-2_ [F24:A-:B1*], pST12 *bla*_CMY-2_ [F18:A-:B6], and pST31 *bla*_CTX-M-15_ [F46:A-:B20]. Also, IncF replicons were found in one IncN plasmid: pST1 *bla*_CTX-M-1_ [F46:A-:B23].

*One allele of the plasmid multilocus sequence typing (pMLST) has 100% identity and 92% coverage.

**pST-NT represent the IncI1 plasmids where some genes of the pMLST scheme were not detected.

“NT” category refers to the non-typeable plasmids, where was not possible to detect plasmid replicons genes through Plasmidfinder and Mob-suite on the plasmid-identified contig. “Chr” represents the chromosome sequence. “-” indicates not applicable, as no data were generated.

Main plasmid sequence type (pST) or IncF FAB formula are highlighted in the table.

ESC-R: extended-spectrum cephalosporin resistance.

**Supplementary Table S5. Percentage of six AMR profiles for the IncI1 pST3 *bla*_CTX-M-1_ plasmid subtype (n=42).**

| **AMR profile** | **# AMR genes** | **Total n (%)** | **All countries – source n (%)** |
| --- | --- | --- | --- |
| *bla*_CTX-M-1_, *aadA5*, *dfrA17*, *sul2** | 4 | 19 (45.2%) | France – Human: 5 (26.3%)  France, Germany – Cattle: 6 (31.6%)  Canada, Germany – Pig: 5 (26.3%)  France, Germany – Other: 3 (15.8%) |
| *bla*_CTX-M-1_, *sul2*, *tet*(A)* | 3 | 19 (45.2%) | France – Human: 3 (15.8%)  France – Cattle: 2 (10.5%)  Canada, Germany – Chicken: 6 (31.6%)  Canada – Pig: 2 (10.5%)  Canada, France – Other: 6 (31.6%) |
| *bla*_CTX-M-1_, *aac(3)-IIa* | 2 | 1 (2.4%) | France – Other: 1 (100%) |
| *bla*_CTX-M-1_, *aadA5*, *dfrA17*, *mef*(C), *mph*(G), *sul2* | 6 | 1 (2.4%) | Germany – Pig: 1 (100%) |
| *bla*_CTX-M-1_, *bla*_TEM-1B_, *sul2*, *tet*(A) | 4 | 1 (2.4%) | Canada – Pig: 1 (100%) |
| *bla*_CTX-M-1_, *sul2* | 2 | 1 (2.4%) | Canada – Pig: 1 (100%) |

Co-occurrence of AMR genes belonging to the antimicrobial classes: ESBL (*bla*_CTX-M-1_), aminoglycosides (*aadA5* and *aac(3)-IIa*), beta-lactams (*bla*_TEM-1B_), diaminopyrimidines (*dfrA17*), sulphonamides (*sul2*), tetracyclines (*tet*(A)), and macrolides (*mef*(C), and *mph*(G)).

^&^The “other” category consists of other sources such as food (n=5), turkey (n=2), cat (n=1), dog (n=1) and horse (n=1).

*For the two most frequent AMR profiles, the genetic elements associated with these AMR genes were investigated. The *aadA5*-*dfrA17* is a cassette array which was found in class 1 integrons and in a cluster of *attC* sites lacking integron-integrases (CALIN), and the *sul2* and *tet*(A) were found next to IS*26* and IS*91*, respectively (see details supplementary Figure 7).

**Supplementary Table S6. Diversity of sources and *E. coli* STs among the main plasmid subtypes: observed diversity, estimated asymptotic diversity with 95% confidence intervals.**

| **Epidemic plasmid** | **Diversity** | **Observed** | **Estimator** | **s.e.** | **LCL** | **UCL** | **Category** | **Dataset** |
| --- | --- | --- | --- | --- | --- | --- | --- | --- |
| IncI1 pST12 *bla*_CMY-2_ | Species richness | 4.0 | 4.5 | 1.4 | 4.0 | 7.3 | source | A |
|  | Shannon diversity | 2.9 | 3.1 | 0.6 | 2.0 | 4.3 | source | A |
|  | Simpson diversity | 2.5 | 2.7 | 0.5 | 1.8 | 3.6 | source | A |
| IncI1 pST2 *bla*_CMY-2_ | Species richness | 4.0 | 4.2 | 1.8 | 4.0 | 7.7 | source | A |
|  | Shannon diversity | 3.4 | 4.1 | 1.2 | 1.8 | 6.4 | source | A |
|  | Simpson diversity | 2.9 | 3.8 | 1.4 | 0.9 | 6.6 | source | A |
| IncI1 pST3  *bla*_CTX-M-1_ | Species richness | 9.0 | 13.4 | 3.6 | 9.0 | 20.5 | source | A |
|  | Shannon diversity | 6.8 | 8.0 | 1.1 | 5.7 | 10.2 | source | A |
|  | Simpson diversity | 6.0 | 6.8 | 0.9 | 4.9 | 8.6 | source | A |
| IncF F2:A-:B-  *bla*_CTX-M-14_ | Species richness | 4.0 | 5.0 | 0.6 | 4.0 | 6.1 | source | A |
|  | Shannon diversity | 2.6 | 2.9 | 0.3 | 2.2 | 3.5 | source | A |
|  | Simpson diversity | 2.3 | 2.4 | 0.3 | 1.9 | 3.0 | source | A |
| IncF F31:A4:B1 *bla*_CTX-M-15_ | Species richness | 3.0 | 3.0 | 0.5 | 3.0 | 4.0 | source | A |
|  | Shannon diversity | 2.4 | 2.5 | 0.3 | 1.9 | 3.1 | source | A |
|  | Simpson diversity | 2.2 | 2.3 | 0.3 | 1.8 | 2.9 | source | A |
| IncI1 pST12 *bla*_CMY-2_ | Species richness | 14.0 | 42.9 | 24.7 | 14.0 | 91.3 | *E. coli* ST | A |
|  | Shannon diversity | 9.9 | 23.0 | 11.3 | 0.8 | 45.2 | *E. coli* ST | A |
|  | Simpson diversity | 6.4 | 8.4 | 6.2 | 0.0 | 20.5 | *E. coli* ST | A |
| IncI1 pST2 *bla*_CMY-2_ | Species richness | 8.0 | 16.1 | 8.4 | 8.0 | 32.6 | *E. coli* ST | A |
|  | Shannon diversity | 7.6 | 17.7 | 8.4 | 1.2 | 34.2 | *E. coli* ST | A |
|  | Simpson diversity | 7.1 | 22.5 | 10.2 | 2.5 | 42.5 | *E. coli* ST | A |
| IncI1 pST3  *bla*_CTX-M-1_ | Species richness | 19.0 | 101.5 | 41.2 | 20.7 | 182.2 | *E. coli* ST | A |
|  | Shannon diversity | 13.0 | 27.7 | 9.7 | 8.8 | 46.7 | *E. coli* ST | A |
|  | Simpson diversity | 9.4 | 11.8 | 3.1 | 5.7 | 17.9 | *E. coli* ST | A |
| IncF F2:A-:B-  *bla*_CTX-M-14_ | Species richness | 16.0 | 103.5 | 33.6 | 37.7 | 169.3 | *E. coli* ST | A |
|  | Shannon diversity | 11.1 | 37.8 | 14.4 | 9.6 | 66.1 | *E. coli* ST | A |
|  | Simpson diversity | 7.2 | 9.6 | 4.3 | 1.1 | 18.0 | *E. coli* ST | A |
| IncF F31:A4:B1 *bla*_CTX-M-15_ | Species richness | 13.0 | 23.1 | 11.6 | 13.0 | 46.0 | *E. coli* ST | A |
|  | Shannon diversity | 11.7 | 21.7 | 6.2 | 9.5 | 34.0 | *E. coli* ST | A |
|  | Simpson diversity | 10.5 | 21.1 | 6.0 | 9.3 | 32.9 | *E. coli* ST | A |
| IncI1 pST12 *bla*_CMY-2_ | Species richness | 6.0 | 7.0 | 1.3 | 6.0 | 9.6 | source | B |
|  | Shannon diversity | 2.9 | 3.0 | 0.3 | 2.4 | 3.6 | source | B |
|  | Simpson diversity | 2.3 | 2.4 | 0.2 | 1.9 | 2.8 | source | B |
| IncI1 pST2 *bla*_CMY-2_ | Species richness | 7.0 | 8.9 | 2.7 | 7.0 | 14.2 | source | B |
|  | Shannon diversity | 4.4 | 5.1 | 0.8 | 3.5 | 6.7 | source | B |
|  | Simpson diversity | 3.2 | 3.5 | 0.8 | 1.9 | 5.1 | source | B |
| IncI1 pST3  *bla*_CTX-M-1_ | Species richness | 9.0 | 9.0 | 0.5 | 9.0 | 10.0 | source | B |
|  | Shannon diversity | 6.3 | 6.5 | 0.3 | 5.9 | 7.1 | source | B |
|  | Simpson diversity | 5.5 | 5.6 | 0.3 | 5.0 | 6.2 | source | B |
| IncI1 pST12 *bla*_CMY-2_ | Species richness | 32.0 | 186.3 | 89.9 | 32.0 | 362.5 | *E. coli* ST | B |
|  | Shannon diversity | 12.9 | 26.4 | 7.8 | 11.2 | 41.7 | *E. coli* ST | B |
|  | Simpson diversity | 5.8 | 6.2 | 1.5 | 3.2 | 9.2 | *E. coli* ST | B |
| IncI1 pST2 *bla*_CMY-2_ | Species richness | 22.0 | 63.4 | 34.3 | 22.0 | 130.6 | *E. coli* ST | B |
|  | Shannon diversity | 18.3 | 43.1 | 14.0 | 15.6 | 70.5 | *E. coli* ST | B |
|  | Simpson diversity | 14.8 | 25.5 | 8.4 | 9.0 | 42.0 | *E. coli* ST | B |
| IncI1 pST3  *bla*_CTX-M-1_ | Species richness | 76.0 | 210.5 | 57.4 | 98.0 | 322.9 | *E. coli* ST | B |
|  | Shannon diversity | 43.0 | 73.6 | 10.8 | 52.5 | 94.8 | *E. coli* ST | B |
|  | Simpson diversity | 24.0 | 27.5 | 4.4 | 18.9 | 36.2 | *E. coli* ST | B |

s.e: standard error of the asymptotic estimator; LCL: lower confidence limits; UCL: upper confidence limits; A: contiguous plasmid dataset (n=121); B: plasmid dataset (n=298) recovered from larger collection.

**Supplementary Table S7. Number and proportion of *E. coli* STs carrying the main IncI1 plasmid subtypes.**

| **IncI1 plasmid subtypes** | ***E. coli* ST** | **n** | **percentage** |
| --- | --- | --- | --- |
| **pST3 *bla*_CTX-M-1_**  present in 76 *E. coli* STs | ST602 | 24 | 13% |
|  | ST10 | 14 | 8% |
|  | ST117 | 11 | 6% |
|  | ST57 | 10 | 5% |
|  | ST746 | 10 | 5% |
|  | ST88 | 6 | 3% |
|  | ST167 | 5 | 3% |
|  | ST23 | 5 | 3% |
|  | ST362 | 5 | 3% |
|  | ST58 | 5 | 3% |
|  | ST12 | 4 | 2% |
|  | ST1727 | 4 | 2% |
|  | Unknown ST | 4 | 2% |
|  | ST297 | 3 | 2% |
|  | Doubletons | 20 | 11% |
|  | Singletons | 52 | 29% |
|  | TOTAL | 182 | 100% |
| **pST12 *bla*_CMY-2_** present in 32 *E. coli* STs | ST131* | 30 | 36% |
|  | ST117 | 14 | 17% |
|  | ST155 | 3 | 4% |
|  | ST2040 | 3 | 4% |
|  | Unknown ST | 3 | 4% |
|  | Doubletons | 4 | 5% |
|  | Singletons | 25 | 30% |
|  | TOTAL | 82 | 100% |
| **pST2 *bla*_CMY-2_** present in 22 *E. coli* STs | ST58 | 5 | 15% |
|  | ST4608 | 4 | 12% |
|  | ST167 | 3 | 9% |
|  | Doubletons | 6 | 17% |
|  | Singletons | 16 | 47% |
|  | TOTAL | 34 | 100% |

Singletons refer to a single occurrence of an ST, and doubletons to two occurrences of an ST.

*All ST131 belong to the clade B (*fimH22*).

Frequent STs are highlighted in the table.

**Supplementary Table S8. Results of the Fisher’s Exact Tests for the comparison of the proportion of IncI1 pST12 *bla*_CYM-2_ and pST2 *bla*_CMY-2_ between four main compartments (country + source).**

| **Plasmid subtype** | **Compartment 1 (n; %)^1^** | **Compartment 2 (n; %)^1^** | **sample size** | **p-value** | **Adjusted p -value^2^** |
| --- | --- | --- | --- | --- | --- |
| pST12 *bla*_CMY-2_ | Canada – Chicken  (31/34; 91%) | Canada – Human  (13/14; 93%) | 48 | 1 | 1 |
|  | Canada – Chicken  (31/34; 91%) | France – Human  (8/21; 38%) | 55 | 4.32x10^-5^ | 2.59x10^-4^ |
|  | Canada – Chicken  (31/34; 91%) | Germany – Chicken  (15/16; 94%) | 50 | 1 | 1 |
|  | Canada – Human  (13/14; 93%) | France – Human  (8/21; 38%) | 35 | 0.0015 | 0.009 |
|  | Canada – Human  (13/14; 93%) | Germany – Chicken  (15/16; 94%) | 30 | 1 | 1 |
|  | France – Human  (8/21; 38%) | Germany – Chicken  (15/16; 94%) | 37 | 0.001 | 0.004 |

^1^The number and percentage in the table is in relation to the pST12 *bla*_CMY-2_, while the complementary percentage refers to the pST2 *bla*_CMY-2_, for example the percentage of pST12 *bla*_CMY-2_ in human isolates from France is 38% (8/21) while it is 62% (13/21) for pST2 *bla*_CMY-2_

^2^Adjusted p-value by Bonferroni method.

**Supplementary Table S9. Percentage of 17 AMR profiles for the IncF F31:A4:B1 *bla*_CTX-M-15_ plasmid subtype.**

| **AMR profiles** | **# AMR genes** | **Country – Source n (%)** |
| --- | --- | --- |
| *aac(3)-IIa*, *aac(6')-Ib-cr*, *aadA5*, *aph(3')-Ia*, *bla*_CTX-M-15_, *bla*_OXA-1_, *bla*_TEM-1B_, *dfrA17*, *dfrA5*, *mph*(A), *strA*, *strB*, *sul1*, *sul2*, *tet*(A) | 15 | Germany – Cattle: 1 (5%) |
| *aac(3)-IIa*, *aac(6')-Ib-cr*, *aadA5*, *bla*_CTX-M-15_, *bla*_OXA-1_, *dfrA17*, *mph*(A), *sul1*, *tet*(A) | 9 | Canada – Human: 1 (5%) |
| *aac(3)-IIa*, *aac(6')-Ib-cr*, *aadA5*, *bla*_CTX-M-15_, *bla*_OXA-1_, *dfrA17*, *mph*(A), *sul1*, *tet*(B) | 9 | France, Germany – Human, Cattle: 2 (10%) |
| *aac(3)-IIa*, *aac(6')-Ib-cr*, *aadA5*, *bla*_CTX-M-15_, *dfrA17*, mph(A), *sul1*, *tet*(B) | 8 | Germany – Cattle: 1 (5%) |
| *aac(3)-IIa*, *aac(6')-Ib-cr*, *ant(2'')-Ia*, *bla*_CTX-M-15_, *bla*_OXA-1_, *bla*_TEM-1C_, *cmlA1*, *mph*(A), *sul1*, *tet*(A) | 10 | France – Cattle: 1 (5%) |
| *aac(3)-IIa*, *aac(6')-Ib-cr*, *bla*_CTX-M-15_, *bla*_OXA-1_, *mph*(A), *tet*(A) | 6 | Canada, France – Human: 3 (15%) |
| *aac(3)-IIa*, *aadA5*, *bla*_CTX-M-15_, *dfrA17*, *mph*(A), *sul1* | 6 | France – Human: 1 (5%) |
| *aac(3)-IIa*, *aadA5*, *bla*_CTX-M-15_, *dfrA17*, *mph*(A), *sul1*, *tet*(B) | 7 | Canada – Pig: 1 (5%) |
| *aac(3)-IId*, *aac(6')-Ib-cr*, *aadA5*, *bla*_CTX-M-15_, *bla*_OXA-1_, *bla*_TEM-1B_, *catA1*, *dfrA17*, *mph*(A), *sul1*, *tet*(B) | 11 | Canada – Human: 1 (5%) |
| *aac(6')-Ib-cr*, *aadA5*, *bla*_CTX-M-15_, *bla*_OXA-1_, *dfrA17*, *mph*(A), *sul1*, *tet*(A) | 8 | Germany – Cattle: 1 (5%) |
| *aac(6')-Ib-cr*, *aadA5*, *bla*_CTX-M-15_, *bla*_OXA-1_, *dfrA17*, *mph*(A), *sul1*, *tet*(B) | 8 | France – Cattle: 1 (5%) |
| *aac(6')-Ib-cr*, *aadA5*, *bla*_CTX-M-15_, *bla*_OXA-1_, *dfrA17*, *mph*(A), *sul1* | 7 | France – Cattle: 1 (5%) |
| *aac(6')-Ib-cr*, *aadA5*, *bla*_CTX-M-15_, *bla*_OXA-1_, *dfrA17*, *mph*(A), *tet*(B) | 7 | Canada – Human: 1 (5%) |
| *aac(6')-Ib-cr*, *aadA5*, *bla*_CTX-M-15_, *bla*_OXA-1_, *dfrA17*, *sul1*, *tet*(B) | 7 | Canada – Human: 1 (5%) |
| *aadA5*, *bla*_CTX-M-15_, *catA1*, *dfrA17*, *sul1*, *tet*(B) | 6 | Canada – Human: 1 (5%) |
| *aadA5*, *bla*_CTX-M-15_, *dfrA17*, *floR*, *sul1* | 5 | France – Cattle: 1 (5%) |
| *bla*_CTX-M-15_, *mph*(A), *tet*(A) | 3 | Germany – Cattle: 1 (5%) |
| TOTAL |  | 20 (100%) |

Co-occurrence of *bla*_CTX-M-15_ with other AMR genes belonging to the antimicrobial classes: macrolides (*mph*(A)), aminoglycosides-fluoroquinolones (*aac(6')-Ib-cr*), aminoglycosides (*aadA5*, *aac(3)-IIa*, *aac(3)-IId*, *ant(2'')-Ia*, *aph(3')-Ia*, *strA*, and *strB*), diaminopyrimidines (*dfrA17* and *dfrA5*), sulphonamides (*sul1* and *sul2*), beta-lactams (*bla*_OXA-1_, *bla*_TEM-1B_ and *bla*_TEM-1C_), tetracyclines (*tet*(A) and *tet*(B)), and phenicols (*catA1*, *cmlA1* and *floR*).

The ESC-R gene is included in the number of AMR genes within each profile.

**Supplementary Table S10. Profiles and gene configurations for ESC-R genes in IncI1 plasmids (n=86).** Profiles are constituted by the order of five genes upstream, ESC-R genes and five genes downstream, where gene configuration is the immediately surrounding genes (text highlighted) of the ESC-R gene.

| **# of profile (n)** | **IncI1 pST** | **Five genes to the left of the ESC-R gene** | **ESC-R gene** | **Five genes to the right of the ESC-R gene** | **Each gene configuration** | **Unique gene configuration** |
| --- | --- | --- | --- | --- | --- | --- |
| #1 (n=18) | pST12, NT | hypothetical, hypothetical, *yafB*, *yagA*, IS*Ecp1* | *bla*_CMY-2_ | *blc*, *sugE*, hypothetical, hypothetical, hypothetical | intact IS*Ecp1*- *bla*_CMY-2_-*blc*-*sugE* | **configuration #1 – pST12 *bla*_CMY-2_** |
| #2 (n=1) | pST12 | *yafB*, transposase, IS*3*-like, *yagA*, IS*Ecp1* | *bla*_CMY-2_ | *blc*, *sugE*, hypothetical, hypothetical, hypothetical | intact ISEcp1- *bla*_CMY-2_-*blc*-*sugE* | **configuration #1 – pST12 *bla*_CMY-2_** |
| #3 (n=1) | pST12 | hypothetical, *yafB*, *yagA*, IS*Ecp1*, IS*Ecp1* | *bla*_CMY-2_ | *blc*, *sugE*, hypothetical, hypothetical, hypothetical | truncated IS*Ecp1*- *bla*_CMY-2_-*blc*-*sugE* | configuration #2 – pST12 *bla*_CMY-2_ |
| #4 (n=4) | pST12 | hypothetical, Colicin-Ia, colicin transporter, hypothetical, IS*Ecp1* | *bla*_CMY-2_ | *ydeA*, hypothetical, abortive infection, hypothetical, hypothetical | intact IS*Ecp1*- *bla*_CMY-2_-*ydeA* | **configuration #3 – pST12 *bla*_CMY-2_** |
| #5 (n=1) | pST65 | hypothetical, hypothetical, Colicin-Ia, hypothetical, IS*Ecp1* | *bla*_CMY-2_ | *blc*, *sugE*, *ydeA*, hypothetical, hypothetical | intact IS*Ecp1*- *bla*_CMY-2_-*blc*-*sugE* | **configuration #1 – pST2 *bla*_CMY-2_** |
| #6 (n=1) | pST2 | IS*91*, IS*91*, IS*Ecp1*, IS*Ecp1*, IS*Ecp1* | *bla*_CMY-2_ | *blc*, *sugE*, *yafB*, *yagA*, hypothetical | IS*91*-truncated IS*Ecp1*- *bla*_CMY-2_-*blc*-*sugE* | **configuration #1 – pST2 *bla*_CMY-2_** |
| #7 (n=1) | pST2 | *yacB*, *yacC*, *yadA*, IS*91*, IS*Ecp1* | *bla*_CMY-2_ | *blc*, *sugE*, *sul1*, QACEdelta1, *aac(3)-Via* | IS*91*-truncated IS*Ecp1*- *bla*_CMY-2_-*blc*-*sugE* | **configuration #1 – pST2 *bla*_CMY-2_** |
| #8 (n=1) | pST2 | IS*3*-like, IS*3*-like, recombinase, IS*91*, IS*Ecp1* | *bla*_CMY-2_ | *blc*, *sugE*, IS*Ecp1*, *bla*_CMY-2_, *blc*, *sugE*, *yafB*, *yagA*, hypothetical | IS*91*-truncated IS*Ecp1*- *bla*_CMY-2_-*blc*-*sugE* | **configuration #1 – pST2 *bla*_CMY-2_** |
| #9 (n=7) | pST2, NT | *repZ*, hypothetical, hypothetical, IS*91*, IS*Ecp1* | *bla*_CMY-2_ | *blc*, *sugE*, *yafB*, *yagA*, hypothetical | IS*91*-truncated IS*Ecp1*- *bla*_CMY-2_-*blc*-*sugE* | **configuration #1 – pST2 *bla*_CMY-2_** |
| #10 (n=1) | pST2 | *repZ*, hypothetical, hypothetical, IS*91*, IS*Ecp1* | *bla*_CMY-2_ | *blc*, *sugE*, IS*91*, IS*Ecp1*, *bla*_CMY-2_, *blc*, *sugE*, *yafB*, *yagA*, hypothetical | IS*91*-truncated IS*E*cp1- *bla*_CMY-2_-*blc*-*sugE* | **configuration #1 – pST2 *bla*_CMY-2_** |
| #11 (n=1) | pST2 | hypothetical, hypothetical, IS*91*, IS*91*, IS*Ecp1* | *bla*_CMY-2_ | *blc*, *sugE*, *yafB*, *yagA*, hypothetical | IS*91*-truncated IS*E*cp1- *bla*_CMY-2_-*blc*-*sugE* | **configuration #1 – pST2 *bla*_CMY-2_** |
| #12 (n=3) | pST3 | *tra*F, *tra*E, *rci*, hypothetical, tryptophan synthase | *bla*_CTX-M-1_ | IS*Ecp1*, prepilin, *pilV*, prepilin, *pilT* | tryptophan synthase- *bla*_CTX-M-1_-intact IS*Ecp1* [inverted] | **configuration #1 – pST3 *bla*_CTX-M-1_** |
| #13 (n=2) | pST3 | *rci*, *pilV*, prepilin, hypothetical, tryptophan synthase | *bla*_CTX-M-1_ | IS*Ecp1*, *pilV*, prepilin, *pilT*, *pilX* | tryptophan synthase- *bla*_CTX-M-1_-intact IS*Ecp1* [inverted] | **configuration #1 – pST3 *bla*_CTX-M-1_** |
| #14 (n=14) | pST3 | *tra*F, *tra*E, *rci*, hypothetical, tryptophan synthase | *bla*_CTX-M-1_ | IS*Ecp1*, *pilV*, *pilV*, prepilin, *pilT* | tryptophan synthase- *bla*_CTX-M-1_-intact IS*Ecp1* [inverted] | **configuration #1 – pST3 *bla*_CTX-M-1_** |
| #15 (n=1) | pST3 | *tra*F, *tra*E, *rci*, hypothetical, tryptophan synthase | *bla*_CTX-M-1_ | IS*Ecp1*, *pilV*, prepilin, *pilT*, *pilX* | tryptophan synthase- *bla*_CTX-M-1_-intact IS*Ecp1* [inverted] | **configuration #1 – pST3 *bla*_CTX-M-1_** |
| #16 (n=2) | pST63, pST58 | hypothetical, hypothetical, Colicin-Ia, hypothetical, tryptophan synthase | *bla*_CTX-M-1_ | IS*Ecp1*, hypothetical, hypothetical, hypothetical, site-specific integrase | tryptophan synthase- *bla*_CTX-M-1_-intact IS*Ecp1* [inverted] | **configuration #1 – pST3 *bla*_CTX-M-1_*** |
| #17 (n=1) | pST3 | *tra*E, *rci*, *pilV*, hypothetical, tryptophan synthase | *bla*_CTX-M-1_ | IS*Ecp1*, *pilV*, prepilin, *pilT*, *pilX* | tryptophan synthase- *bla*_CTX-M-1_-intact IS*Ecp1* [inverted] | **configuration #1 – pST3 *bla*_CTX-M-1_** |
| #18 (n=3) | pST3, NT | *tra*E, *rci*, prepilin, *pilV*, IS*Ecp1* | *bla*_CTX-M-1_ | tryptophan synthase, hypothetical, *pilV*, prepilin, *pilT* | intact IS*Ecp1*- *bla*_CTX-M-1_-tryptophan synthase | **configuration #2 – pST3 *bla*_CTX-M-1_** |
| #19 (n=1) | pST3 | *tra*E, *rci*, IS*Ecp1*, IS*Kpn26*, IS*Ecp1* | *bla*_CTX-M-1_ | tryptophan synthase, hypothetical, prepilin, *pilV*, prepilin | IS*Kpn26*-truncated IS*Ecp1*- *bla*_CTX-M-1_-tryptophan synthase | **configuration #3 – pST3 *bla*_CTX-M-1_** |
| #20 (n=3) | pST3 | *tra*E, *rci*, IS*Ecp1*, IS*Kpn26*, IS*Ecp1* | *bla*_CTX-M-1_ | tryptophan synthase, hypothetical, *pilV*, *pilV*, prepilin | IS*Kpn26*-truncated IS*Ecp1*- *bla*_CTX-M-1_-tryptophan synthase | **configuration #3 – pST3 *bla*_CTX-M-1_** |
| #21 (n=1) | pST3 | *rci*, IS*Kpn26*, IS*Ecp1*, IS*Kpn26*, IS*Ecp1* | *bla*_CTX-M-1_ | tryptophan synthase, hypothetical, *pilV*, *pilV*, prepilin | IS*Kpn26*-truncated IS*Ecp1*- *bla*_CTX-M-1_-tryptophan synthase | **configuration #3 – pST3 *bla*_CTX-M-1_** |
| #22 (n=4) | pST3 | *tra*G, *tra*F, *tra*E, *rci*, IS*Ecp1* | *bla*_CTX-M-1_ | tryptophan synthase, hypothetical, prepilin, *pilV*, prepilin | intact IS*Ecp1*- *bla*_CTX-M-1_-tryptophan synthase | **configuration #2 – pST3 *bla*_CTX-M-1_** |
| #23 (n=9) | pST3 | *tra*G, *tra*F, *tra*E, *rci*, IS*Ecp1* | *bla*_CTX-M-1_ | tryptophan synthase, hypothetical, *pilV*, *pilV*, prepilin | intact IS*Ecp1*- *bla*_CTX-M-1_-tryptophan synthase | **configuration #2 – pST3 *bla*_CTX-M-1_** |
| #24 (n=1) | pST3 | *tra*G, *tra*F, *tra*E, *rci*, IS*Ecp1* | *bla*_CTX-M-1_ | tryptophan synthase, hypothetical, *pilV*, IS*Ecp1*, *bla*_CTX-M-1_, tryptophan synthase, *pilV*, prepilin, *pilT*, *pilX* | intact IS*Ecp1*- *bla*_CTX-M-1_-tryptophan synthase | **configuration #2 – pST3 *bla*_CTX-M-1_** |
| #25 (n=2) | pST31 | *yafB*, *yagA*, hypothetical, hypothetical, *orf477* | *bla*_CTX-M-15_ | IS*Ecp1*, *bla*_TEM-1B_, *ImpB*, *ImpA*, *ImpC* | *orf477*- *bla*_CTX-M-15_-intact IS*Ecp1* [inverted] | configuration #4 – pST3 *bla*_CTX-M-15_* |
| #26 (n=1) | pST3 | *tra*F, *tra*E, *rci*, hypothetical, *orf477* | *bla*_CTX-M-15_ | IS*Ecp1*, *pilV*, *pilV*, prepilin, *pilT* | *orf477*- *bla*_CTX-M-15_-intact IS*Ecp1* [inverted] | configuration #4 – pST3 *bla*_CTX-M-15_ |
| #27 (n=1) | pST166 | hypothetical, hypothetical, hypothetical, Colicin-Ia, IS*Ecp1* | *bla*_CTX-M-14_ | IS*903D*, IS*5*, hypothetical, *ydeA*, hypothetical | intact IS*Ecp1*- *bla*_CTX-M-14_-IS*903D* | other configuration – *bla*_CTX-M-14_ |

*The gene configuration #1 that was found in pST3 was also observed in other plasmid subtypes such as pST63 and pST58. The gene configuration #4 that was found in pST3 was also found in pST31.
Text in bold represents the frequent gene configurations.

**Supplementary Table S11. Profiles and gene configurations for ESC-R genes in IncF plasmids (n=70).** Profiles are constituted by the order of five genes upstream, ESC-R genes and five genes downstream, where gene configuration is the immediately surrounding genes (text highlighted) of the ESC-R gene.

| **# of profile (n)** | **IncF FAB formula** | **Five genes to the left of the ESC-R gene** | **ESC-R gene** | **Five genes to the right of the ESC-R gene** | **Each gene configuration** | **Unique gene configuration** |
| --- | --- | --- | --- | --- | --- | --- |
| #1 (n=1) | F31:A4:B1 | *ycdA*, D2, Putative_Toxin-antitoxin, *bla*_TEM-1C_, Tn*3*_transposase, IS*Ecp1* | *bla*_CTX-M-15_ | tryptophan synthase, Tn*3* transposase, *tnpA*_IS*26*, *orfB*, transposase | intact IS*Ecp1*-*bla*_CTX-M-15_-tryptophan synthase-Tn*3*-IS*26* | configuration #1 - F31:A4:B1 |
| #2 (n=1) | F31:A4:B1 | *dfrA17*, D2, *intI1*, t*npM*, *tnpA* IS*26*, IS*Ecp1* | *bla*_CTX-M-15_ | tryptophan synthase, Tn*3* transposase, *tnpA*_IS*26*, *bla*_OXA-1_, *aac(6')-Ib-cr* | IS*26*-intact IS*Ecp1*-*bla*_CTX-M-15_-tryptophan synthase-Tn*3*-IS*26* | configuration #2 - F31:A4:B1 |
| #3 (n=1) | F31:A4:B1 | *aadA5*, D2, *dfrA17*, *intI1*_integrase, tnpA IS*26*, IS*Ecp1* | *bla*_CTX-M-15_ | tryptophan synthase, Tn*3* transposase, *tnpA*_IS*26*, transposase, tunicamycin | IS*26*-intact IS*Ecp1*-*bla*_CTX-M-15_-tryptophan synthase-Tn*3*-IS*26* | configuration #2 - F31:A4:B1 |
| #4 (n=1) | F31:A4:B1 | *dfrA17*, D2, i*ntI1*_integrase, *tnpM*, tnpA IS*26*, IS*Ecp1* | *bla*_CTX-M-15_ | tryptophan synthase, Tn*3* transposase, *tnpA*_IS*26*, transposase, tunicamycin | IS*26*-intact IS*Ecp1*-*bla*_CTX-M-15_-tryptophan synthase-Tn*3*-IS*26* | configuration #2 - F31:A4:B1 |
| #5 (n=3) | F31:A4:B1 | *dfrA17*, D2, *intI1*, TnpM, tnpA IS*26*, IS*Ecp1* | *bla*_CTX-M-15_ | tryptophan synthase, Tn*3* transposase, *tnpA*_IS*26*, transposase, hypothetical | IS*26*-intact IS*Ecp1*-*bla*_CTX-M-15_-tryptophan synthase-Tn*3*-IS*26* | configuration #2 - F31:A4:B1 |
| #6 (n=1) | F31:A4:B1 | *dfrA17*, D2, *intl1*, TnpM, tnpA IS*26*, IS*Ecp1* | *bla*_CTX-M-15_ | tryptophan synthase, Tn*3* transposase, *tnpA*_IS*26*, *orfB*, hypothetical | IS*26*-intact IS*Ecp1*-*bla*_CTX-M-15_-tryptophan synthase-Tn*3*-IS*26* | configuration #2 - F31:A4:B1 |
| #7 (n=1) | F31:A4:B1 | hypothetical, D2, reverse transcriptase, IS*Sfl3*, tnp*A* IS*26*, IS*Ecp1* | *bla*_CTX-M-15_ | tryptophan synthase, Tn*3* transposase, *tnpA*_IS*26*, *mrx*, *mphR*(A) | IS*26*-truncated IS*Ecp1*-*bla*_CTX-M-15_-tryptophan synthase-Tn3-IS*26* | configuration #3 - F31:A4:B1 |
| #8 (n=1) | F31:A4:B1 | *dfrA17*, D2, *intI1*, *tnpM*, *tnpA*_IS*26*, IS*Ecp1* | *bla*_CTX-M-15_ | tryptophan synthase, *tnpA*_IS*26*, *bla*_OXA-1_, *aac(6')-Ib-cr*, transposase | IS*26*-intact IS*Ecp1*-*bla*_CTX-M-15_-tryptophan synthase-IS*26* | configuration #4 - F31:A4:B1 |
| #9 (n=1) | F-:A-:B53 | *tnpR*, D2, DUF4158, IS*1*-*insB*, IS*1*-*insA*, IS*Ecp1* | *bla*_CTX-M-15_ | tryptophan synthase, *tnpA_*Tn*2*, *qnrS1*, *tnpA*_IS*26*, *tnpR* | intact IS*Ecp1*-*bla*_CTX-M-15_-tryptophan synthase-Tn2 | other configuration |
| #10 (n=1) | F22:A1:B20 | tunicamycin, D2, transposase, *tnpA*_IS*26*, Tn*3* transposase, *orf477* | *bla*_CTX-M-15_ | IS*Ecp1*, *tnpA*_IS*26*, *bla*_OXA-1_, *aac(6')-Ib-cr*, *tnpA*_IS*26* | IS*26*-Tn*3*-*orf477*-*bla*_CTX-M-15_-truncanted IS*Ecp1*-IS*26* [inverted] | configuration #5 - F31:A4:B1** |
| #11 (n=1) | F31:A4:B1 | *mrx*, D2, *mph*(A), *tnpA*_IS*26*, Tn*3* transposase, *orf477* | *bla*_CTX-M-15_ | IS*Ecp1*, *tnpA*_IS*26*, IS*Sfl3*, IS*3* family transposase, *ydfA* | IS*26*-Tn*3*-*orf477*-*bla*_CTX-M-15_-truncanted IS*Ecp1*-IS*26* [inverted] | configuration #5 - F31:A4:B1 |
| #12 (n=1) | F31:A4:B1 | hypothetical, D2, *insF1*, , *tnpA*_IS*26*, Tn*3* transposase, *orf477* | *bla*_CTX-M-15_ | IS*Ecp1*, *tnpA*_IS*26*, *bla*_OXA-1_, *aac(6')-Ib-cr*, transposase Tn*3* | IS*26*-Tn*3*-*orf477*-*bla*_CTX-M-15_-truncanted IS*Ecp1*-IS*26* [inverted] | configuration #5 - F31:A4:B1 |
| #13 (n=1) | F2:A-:B- | hypothetical, D2, Tir, *tnpA*_IS*26*, Tn*3* transposase, *wbuC* | *bla*_CTX-M-15_ | *tnpA*_IS*26*, *ycdA*, *ycdB*, *stbB*, *stbA* | IS*26*-Tn*3*-*wbuC*-*bla*_CTX-M-15_-IS*26* [inverted] | configuration #1 - F2:A-:B- |
| #14 (n=1) | F2:A1:B- | *bla*_OXA-1_, D2*, aac(6')-Ib-cr*, , *tnpA*_IS*26*, Tn*3* transposase, *wbuC* | *bla*_CTX-M-15_ | *tnpA*_IS*26*, recombinase, *tnpM*, transcriptional regulator, *tnpM* | IS*26*-Tn*3*-*wbuC*-*bla*_CTX-M-15_-IS*26* [inverted] | configuration #1 - F2:A-:B- |
| #15 (n=1) | F35:A-:B- | *repA*, D2, *repA4*, hypothetical, transposase, *wbuC* | *bla*_CTX-M-15_ | IS*Ecp1*, tnpA, *tnpA*_IS*26*, *erm*(B), *insB* | *wbuC*-*bla*_CTX-M-15_-intact ISEcp1-IS*26* [inverted] | other configuration |
| #16 (n=1) | F31:A4:B1 | *tnpA*_IS*26*, D2, *bla*_OXA-1_, *aac(6')-Ib-cr*, Tn*3* transposase, *wbuC* | *bla*_CTX-M-15_ | IS*Ecp1*, Tn*3*_transposase, transposase, histidine acid, hypothetical | Tn*3-wbuC*-*bla*_CTX-M-15_-intact IS*Ecp1* [inverted] | configuration #6 - F31:A4:B1 |
| #17 (n=1) | F31:A4:B1 | aac(6')-Ib-cr, D2, *bla*_OXA-1_, *catB4*, Tn*3* transposase, *wbuC* | *bla*_CTX-M-15_ | IS*Ecp1*, *tnpA*_IS*26*, *tnpM*, *intI1*, *dfrA17* | Tn*3-wbuC*-*bla*_CTX-M-15_-intact IS*Ecp1* [inverted] | configuration #6 - F31:A4:B1 |
| #18 (n=1) | F31:A4:B1 | *aac(6')-Ib-cr*, D2, *bla*_OXA-30_, *catB4*, *tnpA*_IS*26*, *wbuC* | *bla*_CTX-M-15_ | IS*Ecp1*, *tnpA*_IS*26*, transcriptional regulator, dihydrofolate reductase, *intI1*_integrase | IS26-WbuC- *bla*_CTX-M-15_-intact ISEcp1-IS26 [inverted] | configuration #7 - F31:A4:B1 |
| #19 (n=1) | F31:A4:B1 | orfC_IS*629*, D2, reverse_transcriptase, *tnpA*_IS*26*, Tn*3* transposase, *amb* | *bla*_CTX-M-15_ | IS*Ecp1*, *tnpA*_IS*26*, *bla*_OXA-1_, *aac(6')-Ib-cr*, *tnpA*_IS*26* | IS*26*-Tn*3*-*amb*-*bla*_CTX-M-15_-truncated IS*Ecp1*-IS*26* [inverted] | configuration #8 - F31:A4:B1 |
| #20 (n=1) | F31:A4:B1 | hypothetical, D2, *orfA*, *tnpA*_IS*26*, Tn*3* transposase, *amb* | *bla*_CTX-M-15_ | IS*Ecp1*, *tnpA*_IS*26*, *bla*_OXA-1_, *aac(6')-Ib-cr*, *tnpA*_IS*26* | IS*26*-Tn*3*-*amb*-*bla*_CTX-M-15_-truncated IS*Ecp1*-IS*26* [inverted] | configuration #8 - F31:A4:B1 |
| #21 (n=1) | F2:A-:B- | hypothetical, D2, CPBP, *tnpA*_IS*26*, Tn*3* transposase, *amb* | *bla*_CTX-M-15_ | *tnpA*_IS*26*, *ycdA*, *ycdB*, *stbB*, *parM* | IS*26*-Tn*3*-*amb*-*bla*_CTX-M-15_-IS*26* [inverted] | configuration #2 - F2:A-:B- |
| #22 (n=1) | F31:A4:B1 | *mrx*, D2, *mph*(A), *tnpA*_IS*26*, Tn*3* transposase, *amb* | *bla*_CTX-M-15_ | IS*Ecp1*, *tnpA*_IS*26*, *aac(6')-Ib-cr*, *bla*_OXA-30_, transposase Tn*3* | IS*26*-Tn*3*-*amb*-*bla*_CTX-M-15_-truncated IS*Ecp1*-IS*26* [inverted] | configuration #8 - F31:A4:B1 |
| #23 (n=1) | F31:A4:B1 | *aac(3)-IIa*, D2, tunicamycin, *tnpA*_IS*26*, Tn*3* transposase, *amb* | *bla*_CTX-M-15_ | IS*Ecp1*, *tnpA*_IS*26*, *bla*_OXA-1_, *aac(6')-Ib-cr*, transposase Tn*3* | IS*26*-Tn*3*-*amb*-*bla*_CTX-M-15_-truncated IS*Ecp1*-IS*26* [inverted] | configuration #8 - F31:A4:B1 |
| #24 (n=1) | F2:A-:B- | *tnpA*_IS*26*, D2, *intI1*, *tnpA*_IS*26*, *bla*_TEM-1B_, *amb* | *bla*_CTX-M-15_ | IS*Ecp1*, *tnpA*_IS*26*, *ycdA*, *ycdB*, *stbB* | *amb*-*bla*_CTX-M-15_-truncated IS*Ecp1*-IS*26* [inverted] | configuration #3 - F2:A-:B- |
| #25 (n=1) | F31:A4:B1 | *aadA5*, D2, putative ethidium bromide, *sul1*, hypothetical, *tnpA*_IS*26* | *bla*_CTX-M-15_ | tryptophan synthase, Tn*3* transposase, *tnpA*_IS*26*, *floR*, *tnpA*_IS*26* | *bla*_CTX-M-15_-tryptophan synthase-Tn*3* | configuration #9 - F31:A4:B1 |
| #26 (n=1) | F2:A-:B- | *aacA4*, D2, *cmlA*, *qacEΔ1*, *mcp*, IS*903D* | *bla*_CTX-M-14_ | IS*Ecp1*, *tnpA*_IS*26*, Putative *copG*, Putative_Toxin-antitoxin, *insB* | IS*903D*-*bla*_CTX-M-14_-truncated IS*Ecp1*-IS*26* [inverted] | configuration #4 - F2:A-:B- |
| #27 (n=1) | F2:A2:B- | *traK*, D2, *traB*, *tnpA*_IS*26*, *iroN*, IS*903D* | *bla*_CTX-M-14_ | IS*Ecp1*, Tn*3* transposase, *tnpA*_IS*26*, *pdcB*, *pdcA* | IS*903D*-*bla*_CTX-M-14_-intact IS*Ecp1*-truncated Tn*3*-IS*26* [inverted] | other configuration |
| #28 (n=1) | F31:A-:B6* | *repA*, D2, *tnpA*_IS*26*, *iroN*, transposase, IS*903D* | *bla*_CTX-M-14_ | IS*Ecp1*, *mcp*, *qacEΔ1*, *aadA5*, *dfrA17* | IS*903D*-*bla*_CTX-M-14_-intact *ISEcp1* [inverted] | other configuration |
| #29 (n=2) | F2:A-:B- | *relE*/*parE*, D2, 3'-5'_exonuclease, IS*91*, hypothetical, IS*5* | *bla*_CTX-M-14_ | ISEcp1, MFS, ABC_transporter, iron ABC, ABC_transporter | IS*5*-*bla*_CTX-M-14_-intact IS*Ecp1* [inverted] | **configuration #5 - F2:A-:B-** |
| #30 (n=19) | F2:A-:B- | ABC_transporter, D2, *relE*/*parE*, 3'-5'_exonuclease, hypothetical, IS*5* | *bla*_CTX-M-14_ | IS*Ecp1*, MFS, ABC_transporter, iron ABC, ABC_transporter | IS*5*-*bla*_CTX-M-14_-intact IS*Ecp1* [inverted] | **configuration #5 - F2:A-:B-** |
| #31 (n=1) | F2:A-:B- | *repA1*, D2, *repA4*, ABC_transporter, *relE*/*parE*, IS91 | *bla*_CTX-M-14_ | IS*Ecp1*, MFS, ABC_transporter, iron ABC, ABC_transporter | IS*91*-*bla*_CTX-M-14_-intact IS*Ecp1* [inverted] | configuration #6 - F2:A-:B- |
| #32 (n=1) | F24:A-:B1* | IS*2*, D2, iron_ABC, ABC_transporter, MFS, IS*Ecp1* | *bla*_CTX-M-14_ | IS5, hypothetical, 3'-5'_exonuclease, *relE*/*parE*, ABC_transporter | intact IS*Ecp1*- *bla*_CTX-M-14_-IS*5* | other configuration |
| #33 (n=5) | F2:A-:B- | *repC*, D2, *floR*, *tnpA*_IS*26*, Tn*3* transposase, IS*Ecp1* | *bla*_CTX-M-14_ | IS903D, *tnpA*_IS*26*, *ycdA*, *ycdB*, *stbB* | IS*26*-Tn*3*-intact IS*Ecp1*-*bla*_CTX-M-14_-IS*903D*-IS*26* | **configuration #7 - F2:A-:B-** |
| #34 (n=1) | F2:A-:B- | tnpA_IS26, D2, floR, *tnpA*_IS*26*, Tn*3* transposase, IS*Ecp1* | *bla*_CTX-M-14_ | IS903D, *tnpA*_IS*26*, *ycdA*, *ycdB*, *stbB* | IS*26*-Tn*3*-intact IS*Ecp1*-*bla*_CTX-M-14_-IS*903D*-IS*26* | **configuration #7 - F2:A-:B-** |
| #35 (n=1) | F1:A2:B20* | *repA4*, D2, *repA1*, *repA2*, *srnB*, IS*66*-like | *bla*_CTX-M-14_ | IS*Ecp1*, *tnpA*_IS*26*, *psiA*, *psiB*, *yefA* | IS*66*-*bla*_CTX-M-14_-truncated IS*Ecp1*-IS*26* [inverted] | other configuration |
| #36 (n=1) | F2:A-:B45* | Transposase_*orfC*_of_IS*Cro1*/IS*66*_family, D2, Transposase_*orfB*_of_IS*Cro1*/IS*66*_family, IS*1*-*insA*, *insB2*, IS*Ecp1* | *bla*_CTX-M-1_ | *orf477*, hypothetical, Transposase *orfC* of IS*Cro1*/IS*66* family, transposase, transposase | intact IS*Ecp1*-*bla*_CTX-M-1_-*orf477* | configuration #8 - F2:A-:B-** |
| #37 (n=1) | F59:A-:B- | D2, *repA1*, *repA4*, IS*Ecp1* | *bla*_CTX-M-1_ | *orf477*, phage related protein, Addiction module antidote protein, hypothetical, putative DnaJ-class | intact IS*Ecp1*-*bla*_CTX-M-1_-*orf477* | configuration #8 - F2:A-:B-** |
| #38 (n=1) | F2:A-:B- | *trbF*, D2, conjugal transfer protein, putative conjugal transfer protein, conjugal transfer protein, IS*Ecp1* | *bla*_CTX-M-1_ | *orf477*, *traD*, *traI*, *traX*, Alpha/beta | intact IS*Ecp1*-*bla*_CTX-M-1_-*orf477* | configuration #8 - F2:A-:B- |
| #39 (n=1) | F2:A-:B1* | hypothetical, D2, *tnpA*_IS*26*, *mph*(A), *mrx*, *orf477* | *bla*_CTX-M-1_ | *tnpA*_IS*26*, *chrA*, hypothetical, *sul1*, *qacEΔ1* | orf*477*-*bla*_CTX-M-1_-IS*26* [inverted] | other configuration |
| #40 (n=1) | F40:A-:B- | *trbG*, D2, *traV*, *traR*, type IV secretory pathway, *orf477* | *bla*_CTX-M-1_ | IS*Ecp1*, trbI, *traW*, *traU*, *trbC* | *orf477*-*bla*_CTX-M-1_-intact IS*Ecp1* [inverted] | other configuration |
| #41 (n=2) | F2:A-:B- | 3'-5'_exonuclease, D2, hypothetical, *strB*, *strA*, *tnpA*_IS*26* | *bla*_CTX-M-1_ | *orf477*, *mrx*, *mph*(A), *tnpA*_IS*26*, *aac(3)-IVa* | IS*26*-*bla*_CTX-M-1_-*orf477* | configuration #9 - F2:A-:B- |
| #42 (n=1) | F81*:A-:B- | D2, *repA1*, *repA4*, *tnpA* Tn*3*, *tnpA*_IS*26* | *bla*_CTX-M-1_ | *orf477*, IS*91*, *sul2*, *strA*, *strB* | Tn*3*-IS*26*-*bla*_CTX-M-1_-*orf477*-IS*91* | other configuration |
| #43 (n=1) | F34:A-:B- | *ccdA*, D2, *ccdB*, *resD*, *cmi*, IS*Ecp1* | *bla*_CMY-2_ | *blc*, plasmid partitioning/stability, *parM*, hypothetical, *ycgA* | intact IS*Ecp1*-*bla*_CMY-2_-*blc* | other configuration |
| #44 (n=1) | F4:A-:B1* | *insB*, D2, *ycdB*, *cmi*, phage integrase family protein, IS*Ecp1* | *bla*_CMY-2_ | hypothetical, DNA repair protein, *tnpA*_IS*5*, Tn*3* transposase, transposase | intact IS*Ecp1*-*bla*_CMY-2_-hypothetical | other configuration |

**Same gene configuration that was found in diverse plasmid subtypes.

Text in blue is representing to the gene configurations for the IncF F31:A4:B1 *bla*_CTX-M-15_, while in red represent the gene configuration for the IncF F2:A-:B- *bla*_CTX-M-14_.

Text in bold represents the frequent gene configuration.

# **SUPPLEMENTARY REFERENCES**

1. Sampei G, Furuya N, Tachibana K, Saitou Y, Suzuki T, Mizobuchi K, et al. Complete genome sequence of the incompatibility group I1 plasmid R64. Plasmid. 2010 Sep;64(2):92–103.

2. Fricke WF, McDermott PF, Mammel MK, Zhao S, Johnson TJ, Rasko DA, et al. Antimicrobial resistance-conferring plasmids with similarity to virulence plasmids from avian pathogenic Escherichia coli strains in *Salmonella enterica* serovar Kentucky isolates from poultry. Appl Environ Microbiol. 2009 Sep 15;75(18):5963–71.

3. Labbé G, Edirmanasinghe R, Ziebell K, Nash JHE, Bekal S, Parmley EJ, et al. Complete genome and plasmid sequences of three Canadian isolates of *Salmonella enterica* subsp. *enterica* serovar Heidelberg from human and food sources. Genome Announc. 2016 Feb 25;4(1).

4. Woodford N, Carattoli A, Karisik E, Underwood A, Ellington MJ, Livermore DM. Complete nucleotide sequences of plasmids pEK204, pEK499, and pEK516, encoding CTX-M enzymes in three major *Escherichia coli* lineages from the United Kingdom, all belonging to the international O25:H4-ST131 clone. Antimicrob Agents Chemother. 2009 Oct;53(10):4472–82.

5. Zhao F, Bai J, Wu J, Liu J, Zhou M, Xia S, et al. Sequencing and genetic variation of multidrug resistance plasmids in *Klebsiella pneumoniae*. DeLeo FR, editor. PLoS One. 2010 Apr 12;5(4):e10141.

6. Li J-J, Spychala CN, Hu F, Sheng J-F, Doi Y. Complete nucleotide sequences of *bla*_CTX-M-_harboring IncF plasmids from community-associated *Escherichia coli* strains in the United States. Antimicrob Agents Chemother. 2015 Jun;59(6):3002–7.

7. Ho PL, Lo WU, Wong RCW, Yeung MK, Chow KH, Que TL, et al. Complete sequencing of the FII plasmid pHK01, encoding CTX-M-14, and molecular analysis of its variants among *Escherichia coli* from Hong Kong. J Antimicrob Chemother. 2011 Apr 1;66(4):752–6.

8. Wibberg D, Szczepanowski R, Eikmeyer F, Pühler A, Schlüter A. The IncF plasmid pRSB225 isolated from a municipal wastewater treatment plant’s on-site preflooder combining antibiotic resistance and putative virulence functions is highly related to virulence plasmids identified in pathogenic *E. coli* isolates. Plasmid. 2013 Mar;69(2):127–37.
